# Re-boost immunizations with the peptide-based therapeutic HIV vaccine, Vacc-4x, restores geometric mean viral load set-point during treatment interruption

**DOI:** 10.1371/journal.pone.0210965

**Published:** 2019-01-30

**Authors:** Jürgen K. Rockstroh, David Asmuth, Giuseppe Pantaleo, Bonaventura Clotet, Daniel Podzamczer, Jan van Lunzen, Keikawus Arastéh, Ronald Mitsuyasu, Barry Peters, Nozza Silvia, Darren Jolliffe, Mats Ökvist, Kim Krogsgaard, Maja A. Sommerfelt

**Affiliations:** 1 Bonn University Hospital, HIV Outpatient Clinic, Bonn, Germany; 2 University of California Davis Medical Center, Division of Infectious Diseases, Sacramento, California, United States of America; 3 Lausanne University Hospital, Division of Immunology and Allergy, Lausanne, Switzerland; 4 Hospital Universitari Germans Trias i Pujol, Department of Infectious Diseases, Badalona, Spain; 5 University de Bellvitge, The HIV Unit, Barcelona, Spain; 6 University Medical Center Hamburg-Eppendorf, Department of Medicine, Hamburg, Germany; 7 EPIMED c/o Vivantes Auguste-Viktoria Hospital, Berlin, Germany; 8 UCLA CARE Center, Department of Medicine, Los Angeles, California, United States of America; 9 Guys and St. Thomas’ Hospital Trust, Guys Hospital, Harrison Wing, London, United Kingdom; 10 Kings College London, Guys Hospital, Department of Infectious Diseases, Harrison Wing, London, United Kingdom; 11 San Raffaele Hospital, Department of Infectious Diseases, Milan, Italy; 12 S-Cubed Biometrics Ltd. Oxfordshire, United Kingdom; 13 Bionor Immuno AS, Oslo, Norway; 14 KLIFO, Glostrup, Denmark; Rush University, UNITED STATES

## Abstract

**Background:**

Vacc-4x, a therapeutic HIV vaccine candidate has previously induced a significant reduction in viral load (VL) set-point compared to placebo upon interruption of combination anti-retroviral therapy (ART) (2007/1 study). This study, (2012/1), explored the potential to maintain Vacc-4x effect by re-boosting eligible 2007/1 study participants.

**Methods:**

Participant inclusion required 2007/1 participants to have completed all Vacc-4x immunizations and interrupted ART for up to 26 weeks. At weeks (wk)0 and 2, participants received intradermal (i.d.) Vacc-4x booster immunizations (1.2mg) on ART with GM-CSF (60μg) i.d. as a local adjuvant. ART was interrupted for up to 16 weeks (wk12-wk28). Participants were then followed on ART until wk36. VL set-point, total proviral DNA (pvDNA) and immunogenicity assessed by IFN-γ ELISPOT, T-cell proliferation and delayed type hypersensitivity (DTH) reactions were compared to participants’ values in the 2007/1 study where available.

**Results:**

This open, multicenter, clinical study enrolled 33 participants from 9 clinical trial sites in the US and Europe. In the per-protocol (PP) population, the VL set-point geometric mean (GM) 18162 copies/mL was not significantly changed compared to the 2007/1 study (GM VL 22035 copies/mL), (p = 0.453, n = 18). For participants with available preART VL values, the VL set-point (GM 26279 copies/mL) remained significantly lower than the preART VL set-point (GM 74048 copies/mL, p = 0.021, n = 13). A statistically significant reduction in pvDNA (49%) from baseline to wk4 was observed (p = 0.03, n = 26). DTH responses (wk4) increased significantly from baseline (p = 0.006, n = 30) and compared to the 2007/1 study (p = 0.022, n = 29) whilst the proportion of participants with ELISPOT and T-cell proliferation responses was similar between the two studies.

**Conclusions:**

Vacc-4x booster immunizations safely maintained the mean VL set-point at that established following primary Vacc-4x therapeutic immunization. The reduction in pvDNA during ART supports the potential for Vacc-4x immunization to reduce HIV reservoirs and thereby contribute to combination HIV cure strategies.

## Introduction

Therapeutic HIV vaccination is being investigated as a prospective component of future combination strategies aimed at inducing remission of HIV infection (functional cure). Since the duration of immunity to therapeutic HIV vaccine antigens may wane over time, this study addressed the concept that periodic boosting may be required to maintain therapeutic vaccine effect and thereby contribute to sustained HIV remission.

During remission, HIV is not eradicated but viral burden is reduced to below detection levels allowing for durable and safe interruption of combination antiretroviral therapy (ART) [1]. To date, clinical studies using therapeutic HIV vaccines as monotherapy have not resulted in sufficient reductions of viral burden to prevent viral rebound on treatment interruption [2–5]. Nevertheless, by combining therapeutic vaccines with other interventions having complementary mechanisms of action such as latency reversing agents, broadly neutralizing antibodies and/or cytokines, the effects on viral load (VL) following ART interruption may become substantially improved [6, 7].

Innovative approaches towards HIV functional cure are needed because current ART regimens are unable to eradicate the infection despite effectively controlling VL to below detection levels and reducing transmission risk [8–11]. Consequently, when ART is stopped, and in the absence of any other intervention, the VL set-point and proviral DNA (pvDNA) levels in peripheral blood usually return to preART values [12–14].

The potential for including an immune component in combination functional cure strategies is highlighted by observations that pvDNA levels have been reduced during ART following therapeutic vaccination [15]. Furthermore, since ART intensification strategies have not yet made a substantial impact on HIV burden [16–18]. Efforts are underway to determine whether therapeutic vaccination, as part of an ART intensification strategy, may promote immune-mediated removal of infected cells and ultimately contribute to HIV remission over time [19].

Barriers to achieving a future functional HIV cure lie mainly in observations that HIV persists in reservoirs of infection despite ART. ‘Latent reservoirs’ refers to latently infected cells such as resting (quiescent) CD4 T-cells and long-lived immune memory CD4 T-cells [20] whilst the ‘active reservoir’ refers to infected cells producing cell-associated viral RNA in anatomical sanctuary sites poorly accessed by ART such as lymphatic tissue [21].

Latently infected cells lack viral gene expression and therefore remain unaffected by ART and immune targeting. The latent reservoir is envisaged to be largely maintained through clonal proliferation of resting HIV-infected CD4 T-cells [22–23].

It is considered likely that virus gene expression in active reservoirs may contribute to the elevated immune activation associated with non-AIDS adverse events during ART compared to the general population. Eradicating active reservoirs may therefore impact on the elevated immune activation observed in individuals on ART, which is generally lower in uninfected individuals [24]. Early ART initiation may serve to limit the size of HIV reservoirs facilitating eradication strategies and allowing for prolonged ART-free periods [25–27].

Vacc-4x is a peptide-based therapeutic HIV vaccine candidate that aims to induce cell-mediated immune responses to regions on HIV p24^Gag^ conserved between HIV within the major group M. The rationale for sustaining immune responses to HIV p24^Gag^ comes from earlier studies showing that effective anti-Gag responses, including cell-mediated immune responses, are associated with improved virus control in the absence ART [28]. Vacc-4x has been shown to reduce VL set-point [2] and maintain CD4 counts [29] compared to placebo in a clinical study enrolling 137 participants and which included an ART interruption of up to 6 months (CT-BI Vacc-4x 2007/1, referred to here as the 2007/1 study). The VL set-point was also significantly reduced compared to preART levels in participants that received Vacc-4x but not in the placebo group where the VL set-point returned to preART levels in agreement with other treatment interruption studies [12]. Vacc-4x has also been tested in combination with the latency reversing agent (LRA) romidepsin and showed significant reductions in HIV proviral DNA (pvDNA) both before and after latency reversal, but the reduction was insufficient to delay viral rebound on treatment interruption.[15, 30]. The findings nevertheless support the concept that an immune component can play a role in strategies towards functional HIV cure.

Clinical studies investigating re-boosting of study participants after primary therapeutic HIV vaccination are scarce [31, 32]. Two booster immunizations with Vacc-4x during ART given at least seven years post primary immunization showed the presence of memory responses at baseline [33] and increased responses post-boosting [32]. However, the effect on VL set-point was not determined since participants remained on ART throughout the study. In this study, CT-BI Vacc-4x 2012/1 referred to as 2012/1, participants from the earlier large multi-center 2007/1 study were given two Vacc-4x booster immunizations on ART followed by a second treatment interruption to determine the impact on VL set-point and immune responses measured using interferon (IFN)-γ ELISPOT, T-cell proliferation and delayed type hypersensitivity (DTH). Re-boosting with Vacc-4x was found to be safe and well tolerated. The VL set-point in the 2012/1 study was not significantly different from the set-point achieved in the 2007/1 study but remained significantly reduced compared to preART levels suggesting that reboosting represented a viable means to maintain therapeutic HIV vaccine effect attained following primary immunization.

## Materials and methods

### Study participants and immunization schedule

This prospective open label multi-center follow-up clinical study was carried out from December 2012 to January 2014 and is registered at www.clinicaltrials.gov under the identifier NCT01712256. Participants from the 2007/1 study were eligible if they had completed all six Vacc-4x immunizations over 18 weeks, undergone follow-up on ART for 10 weeks and then stopped ART at week 28 for up to 6 months (until week 52). To enroll in the 2012/1 study, no re-start of ART after the 2007/1 study was required. For inclusion participants had to have documented pre-study CD4 cell count ≥400x10^6^/L and pre-study VL < 300,000 copies/mL. Participants were excluded if they had a reported AIDS-defining illness within the previous year, or malignant disease, or had unacceptable values of hematologic and clinical chemistry patterns as judged by the investigator. Full inclusion/exclusion criteria can be found on www.clinicaltrials.gov.

This 2012/1 study was approved by the ethical committees of all participating institutions respectively and carried out in accordance with the International Conference on Harmonization Guidelines for Good Clinical Practice (GCP) and the ethical principles originating from the Declaration of Helsinki. All participants provided written informed consent. The study protocol is included in S1 File. The CONSORT checklist is included as S1 Table, and the names of the approving institutional review boards can be found in S2 Table. The protocol underwent four amendments. The first amendment included information on the extended shelf-life of the investigational product and clarified the text. The second amendment included proviral DNA measurements without the need for further blood sampling. The fourth amendment was specific for Germany and allowed a meningococcal vaccine as permitted concomitant medication.

The 2012/1 study was divided into five phases. A screening phase (up to 4 weeks), a re-boost phase (4 weeks, with Vacc-4x immunizations at week (wk) 0 and wk2), a follow-up phase on ART until Wk12 (8 weeks), and an ART-free follow-up of 16 weeks. At Week 28, re-initiation of ART was recommended and participants were followed for an additional 8 weeks on ART until Week 36.

As for primary immunizations in the 2007/1 study, the two ‘booster’ Vacc-4x immunizations were administered intradermally (i.d.) using recombinant human granulocyte macrophage colony stimulating factor (rhuGM-CSF) as a local adjuvant [2]. Briefly, Vacc-4x (1.2mg) manufactured by Bachem (Bubendorf, Switzerland) reconstituted in 0.1mL water was given i.d. approximately 10 minutes after i.d. administration of rhuGM-CSF (0.1mL) containing 60μg Leukine manufactured by Bayer HealthCare Pharmaceuticals LLC (Seattle, WA, USA) at the same site.

The criteria for permitting ART interruption at Week 12 were the same as for the 2007/1 study, namely, a CD4 count of ≥350 x 10^6^ cells/L and VL below the level of detection (<50 copies/mL) over the last three months whilst on a stable ART regimen. ART was resumed if CD4 counts fell below 350 x 10^6^ cells/L, or to a level that was ≤50% of the value at week 12, and/or if the VL reached >350 000 copies/mL on two consecutive tests within a two week period [2].

### Laboratory and immunological analyses

Adverse events were monitored throughout the study. Viral load, CD4 and CD8 T-cell counts (Roche Amplipre/COBAS TaqMan HIV-1), clinical chemistry and hematology were carried out by Covance Laboratory Services in Europe (Geneva, Switzerland). Proviral DNA (pvDNA) determinations were carried out at the Division of Immunology and Allergy, Lausanne University Hospital (Lausanne, Switzerland) using a real time PCR (Taqman) validated assay targeting the *gag* gene.

Ex vivo analysis of HIV-specific T-cell responses by IFN-γ ELISPOT and T-cell proliferation were also carried out on cryopreserved peripheral blood mononuclear cells (PBMC) at the Division of Immunology and Allergy, Lausanne University Hospital (Lausanne, Switzerland) as described in Pollard et al. 2014 [2]. All sites preparing T-cells for analysis were first accredited before the study start to ensure preparation of T-cells with recovery >70% and viability of >80% on thawing.

Overlapping 15-mer peptides (off-set by 2 amino acids) corresponding to only the conserved region on p24 targeted by Vacc-4x peptides were synthesized at Schafer (Copenhagen, Denmark) to >95% purity. Participants were defined as ‘Vacc-4x ELISPOT Responders’ and ‘Vacc-4x T-cell Proliferation Responders’ respectively if they had a negative assay response at baseline and a positive assay response at a later time point, or if they had a positive assay response at baseline, and that the assay response at a later time point showed at least a 2-fold increase from baseline.

Delayed type hypersensitivity (DTH) was used as a means to monitor cellular immune responses to Vacc-4x in vivo. DTH testing was carried out before immunization at wk0 and 2 weeks after the last re-boost immunization (wk4). DTH assays involved i.d. injection of Vacc-4x (0.1mL) at a concentration of 4.0 mg/mL in the absence of rhuGM-CSF. As for previous studies, the results were evaluated 48 hours later where a positive DTH test was defined as an area of induration ≥ 10mm^2^ (length x height) [34,35].

### Study outcomes

The primary efficacy outcome of the study was to compare the VL set-point established in this 2012/1 study with the VL set-point for these participants recorded in the 2007/1 study. In addition, a co-primary efficacy end point was to compare the frequency of T-cell responses (IFN-γ ELISPOT and T-cell proliferation) between the 2012/1 and 2007/1 studies.

The secondary efficacy outcomes were to compare the VL set-point in this study with preART VL values (if available), and to assess the effect of re-boost immunizations and treatment interruption on CD4 and CD8 T-cell counts, pvDNA and DTH responses. The DTH responses recorded at wk4 (2 weeks post reboosting) were compared to DTH response reported at Week 18 in the 2007/1 study, which was at the time of the last immunization. Determination of CD4/CD8 ratio was added as an exploratory endpoint summarized over time based on the CD4 and CD8 absolute T-cell counts.

### Statistical analyses

The VL set-point in the 2012/1 study was defined as the mean of VL at wk24 and wk28 in participants that discontinued ART according to the protocol and did not resume until wk28. If participants resumed ART before wk28, the wk24 value alone was used as the set-point. Wk24 and wk28 time points corresponded to a duration of 12 and 16 weeks off ART respectively. The VL set-point in the 2007/1 study was defined as the mean of the VL values at wk48 and wk52 or alternatively, the last two values before restarting ART as long as this occurred on or after wk44. Wk48 and wk52 time points corresponded to a duration of 20 and 24 weeks off ART respectively. The preART VL set-point was defined as the mean of two VL values taken within 6 months of ART initiation for the first time. If a second value was not available in that time-frame, a single preART VL value was used. All VL set-points were log-transformed and the logged set-point used within the analysis. VL set-points were presented as geometric means which were calculated as the antilog of the (mean log_10_ VL set-point + 1) -1. Statistical significance of effects was determined by 2-sided tests with p-value <0.05.

Categorical data were presented as number (n) and percentages. The difference in T-cell responses (ELISPOT and T-cell proliferation) between the 2012/1 and 2007/1 studies was determined using McNemar’s test.

## Results

### Study population and baseline characteristics

**Fig 1:**
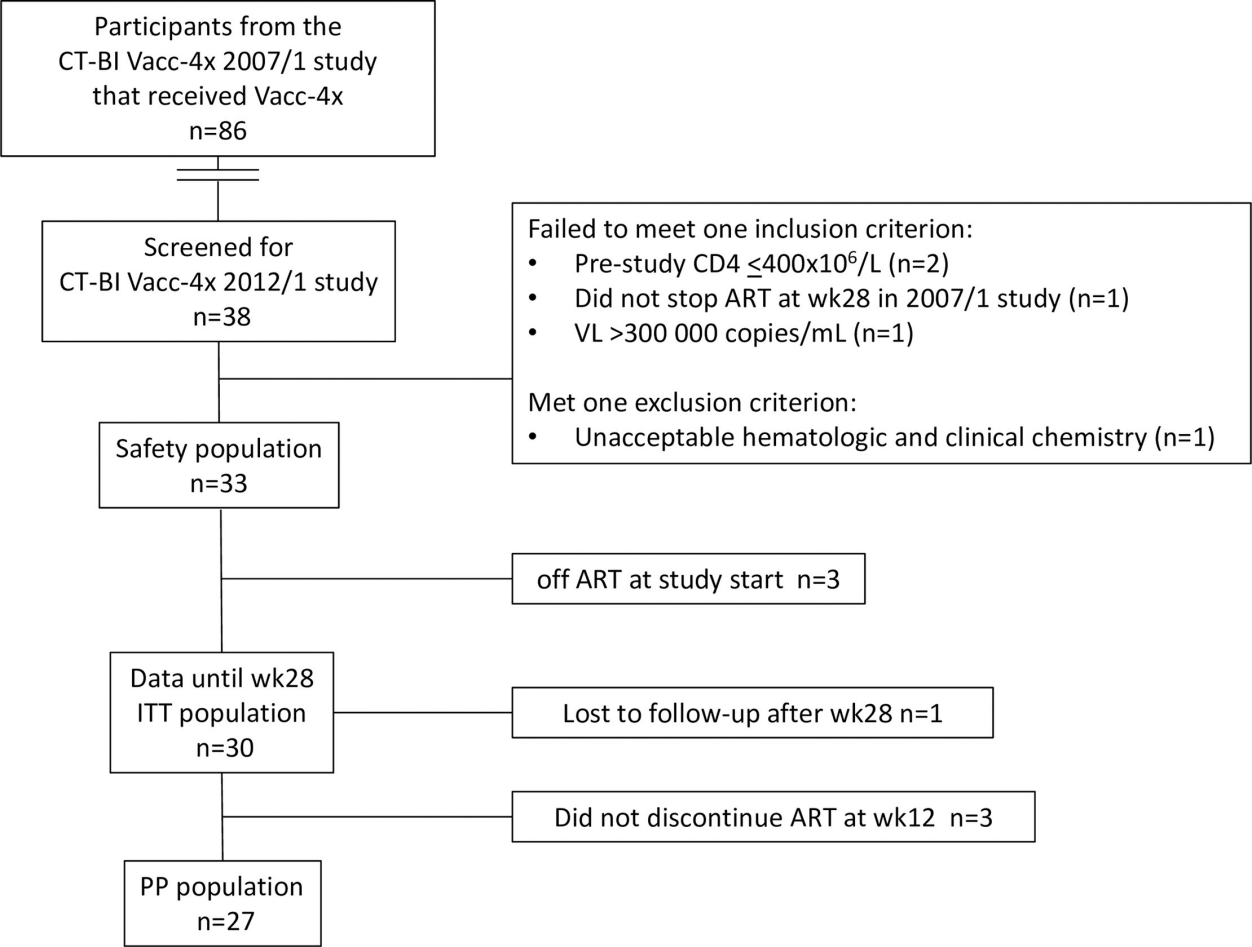
CONSORT diagram for the 2012/1 clinical study. ITT: Intention to treat population. PP: per protocol. One participant was lost to follow-up after completing the week 28 visit, but was included in outcomes analysis in the ITT population. ART: antiretroviral therapy.

All potentially eligible participants from the 2007/1 study that had received the full Vacc-4x immunization regimen and stopped ART at wk28 according to the protocol (n = 86) [2] were invited to participate in the 2012/1 study. A total of 38 responded and were available for screening of which 33 were enrolled from 9 clinical trial sites in five countries (Germany, Italy, Spain, United Kingdom and United States) (Fig 1).

The 2007/1 and 2012/1 studies were separate clinical trials. Since the 2012/1 study took place years after the 2007/1 study, a number of participants (n = 48) did not respond to the invitation to participate and were presumably participating in other clinical studies or lost to follow-up.

The safety population comprised all study participants who had received at least one Vacc-4x re-booster immunization (n = 33). The intention to treat (ITT) population was to include all participants that had received at least one dose of Vacc-4x and had post-baseline efficacy data. However, since the study inclusion did not specify whether participants needed to be on ART at inclusion, three participants were enrolled that had remained off ART since completing the 2007/1 study. These participants remained off ART for the duration of the 2012/1 study and were excluded from the ITT group because their ART-free status would affect the outcomes analysis. One participant was lost to follow-up shortly after week 28, but nevertheless had sufficient data available for inclusion in the ITT group (n = 30).

**Fig 2:**
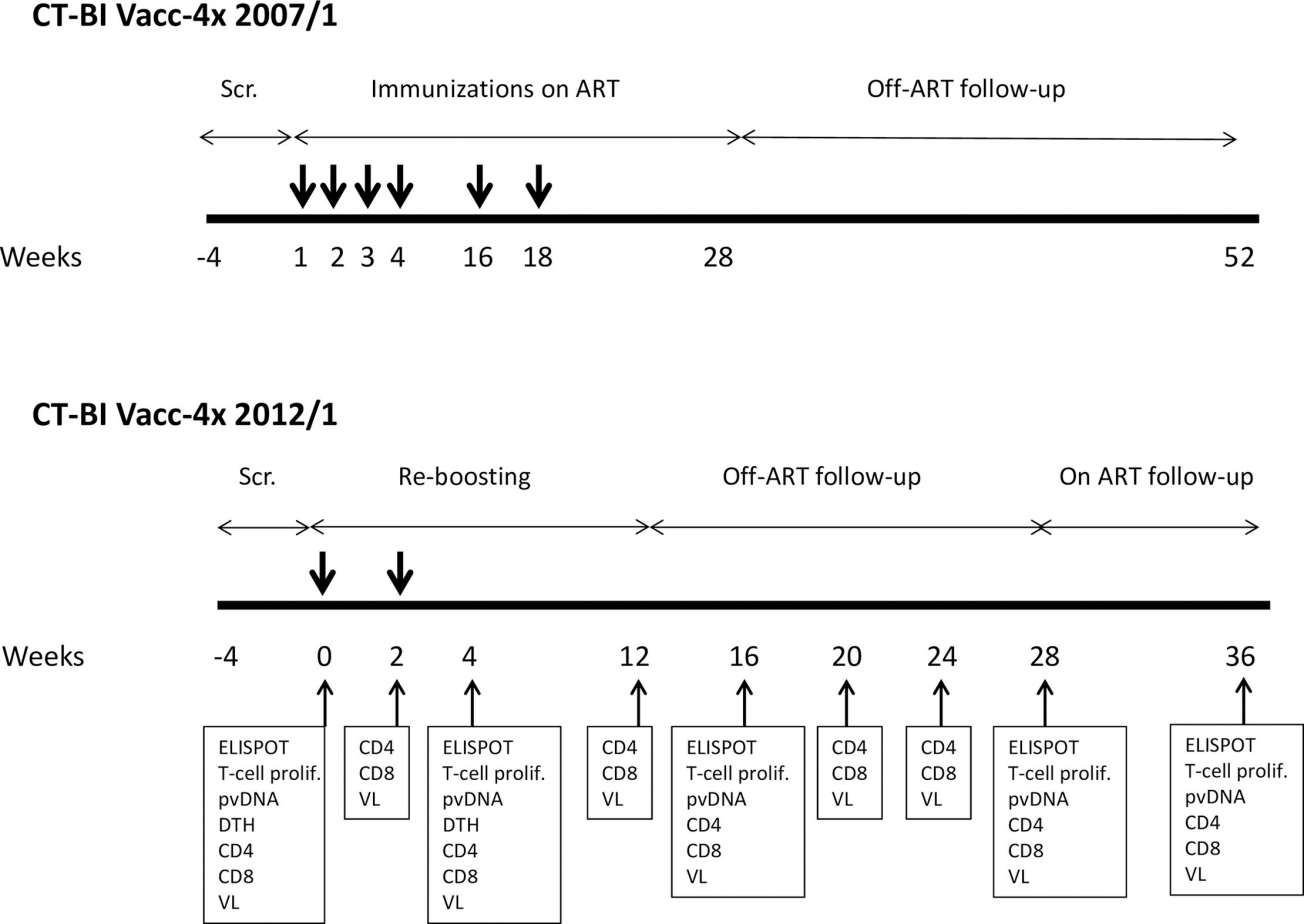
Study schedule for CT-BI Vacc-4x 2007/1 and CT-BI Vacc-4x 2012/1 clinical studies. Thick arrows indicate Vacc-4x immunizations. Analyses carried out during the 2012/1 clinical study are shown. Scr.: Screening; ELISPOT: Enzyme-linked Immunosorbent Assay; T-cell prolif.: T-cell proliferation; pvDNA: proviral DNA; DTH: Delayed type hypersensitivity; CD4 and CD8 T-cell counts; VL: viral load.

The per protocol (PP) group included all participants that had followed the study protocol without any deviations that could affect the validity of the results (n = 27). In addition to the three participants that had not resumed ART since the 2007/1 study, three additional participants were excluded from the PP population, one of which remained on ART throughout the study and two participants that did not stop ART at week 12 according to the protocol (Fig 2). Due to the influence of ART status on VL, and the heterogeneity of ART status amongst the participants throughout the study, the PP population is mainly used to present the outcomes analysis.

Baseline characteristics for the safety population are shown in Table 1. The safety population overall had a longstanding history of HIV infection where the time from HIV diagnosis to inclusion in the study was a median 6148 days (approximately median 16.8 years (range 5.8 o 24.7 years).

**Table 1:** Baseline characteristics for the 2012/1 study safety population (n = 33).

Gender Male n (%) Female n (%)	Current 27 (81.8%)6 (18.2%)	At birth 26 (78.8%) 7 (21.2%)
**Age at screening (years)**		
Median (min to max) n (%)	48 (32–59) 33 (100%)	
**Time since HIV diagnosis (days)**		
Median (min to max) n (%)	6148 (2130–9018) 33 (100%)	
**Total time on ART (months)**		
Median (min to max) n (%)	145 (35–242) 33 (100%)	
**Nadir CD4, cells per μL**^**a**^		
Median (min to max) n (%)	296 (201–454) 33 (100%)	
**PreART CD4, cells per μL**^**a**^		
Median (min to max) n (%)	307 (201–548) 28 (84.8%)	
**PreART HIV-1 VL, copies per mL**^**a**^		
Median (min to max) n (%)	110300 (14185–900000) 25 (75.7%)	
On ART at start of study Off ART at start of study^b^	30 (90.9%) 3 (10%)	
**Baseline CD4, cells per μL (PP)**		
Median (min to max) n (%)	710.5 (488–1883) 27 (100%)	
**Baseline CD8, cells/μL (PP)**		
Median (min to max) n (%)	937 (464–2067.5) 27 (100%)	

Data are n(%) or median (range). PP: per protocol population.

^a^Participant data collected from the 2007/1 study.

^b^Participants who were off ART at the start of the study remained off ART throughout the study period.

All participants on ART at the start of the study (n = 30) were well controlled with VL values <50copies/mL at baseline except for one participant that had a VL of 99 copies/mL at this time point, but was undetectable VL at screening. The ART regimens for these participants is shown in S3 Table.

### Effect of Vacc-4x booster immunizations on viral load set-point

The VL set-point for the 2012/1 study was based on the mean VL values at wks 24 and 28 (n = 16) and from a single value at wk24 (n = 2) for 2 participants. The VL set-point for the 2007/1 study comprised the mean VL from wks 48 and 52 (n = 17) and at wks 40 and 44 (n = 1). This means that the majority of participants had experienced ART-free periods of either 26 weeks (2007/1) study or 16 weeks (2012/1) according to the respective protocols. Participants regained virus control once they resumed ART.

Participants from the PP population that had VL set-points from both the 2007/1 and 2012/1 studies (n = 18) were compared as shown in Table 2.

**Table 2:** Viral load set-point values for the per protocol population that had values for the 2007/1 and 2012/1 studies.

**CT-BI Vacc-4x 2012/1 VL set-point (GM) (n = 18)** GM 95% CI SD	18161.7 8716.59, 37840.14 48923.00
**CT-BI Vacc-4x 2007/1 VL set-point (GM) (n = 18)** GM 95% CI SD	22035.3 11724.98, 41411.36 60759.40
**Geometric mean ratio (2012/1 VL set-point/2007/1 VL set-point**^**a**^ **(n = 18)** GM 95% CI Paired t-test p-value^b^	0.82 0.484, 1.402 0.453

VL: Viral load; PP: per protocol; GM: geometric mean; CI: confidence interval; SD: standard deviation. Geometric means were calculated as the antilog of the mean (mean log_10_ VL set-point +1) -1.

^a^Calculated as the antilog{mean [(2012/1 log10 VL set-point +1)]}.

^b^Paired t-test on the difference in logged set-point values.

The VL set-point in the 2012/1 study in the PP population had a geometric mean (GM) of 18161.70 which was not significantly different from that recorded in the 2007/1 study (GM 22035.30), (p = 0.453).

**Fig 3:**
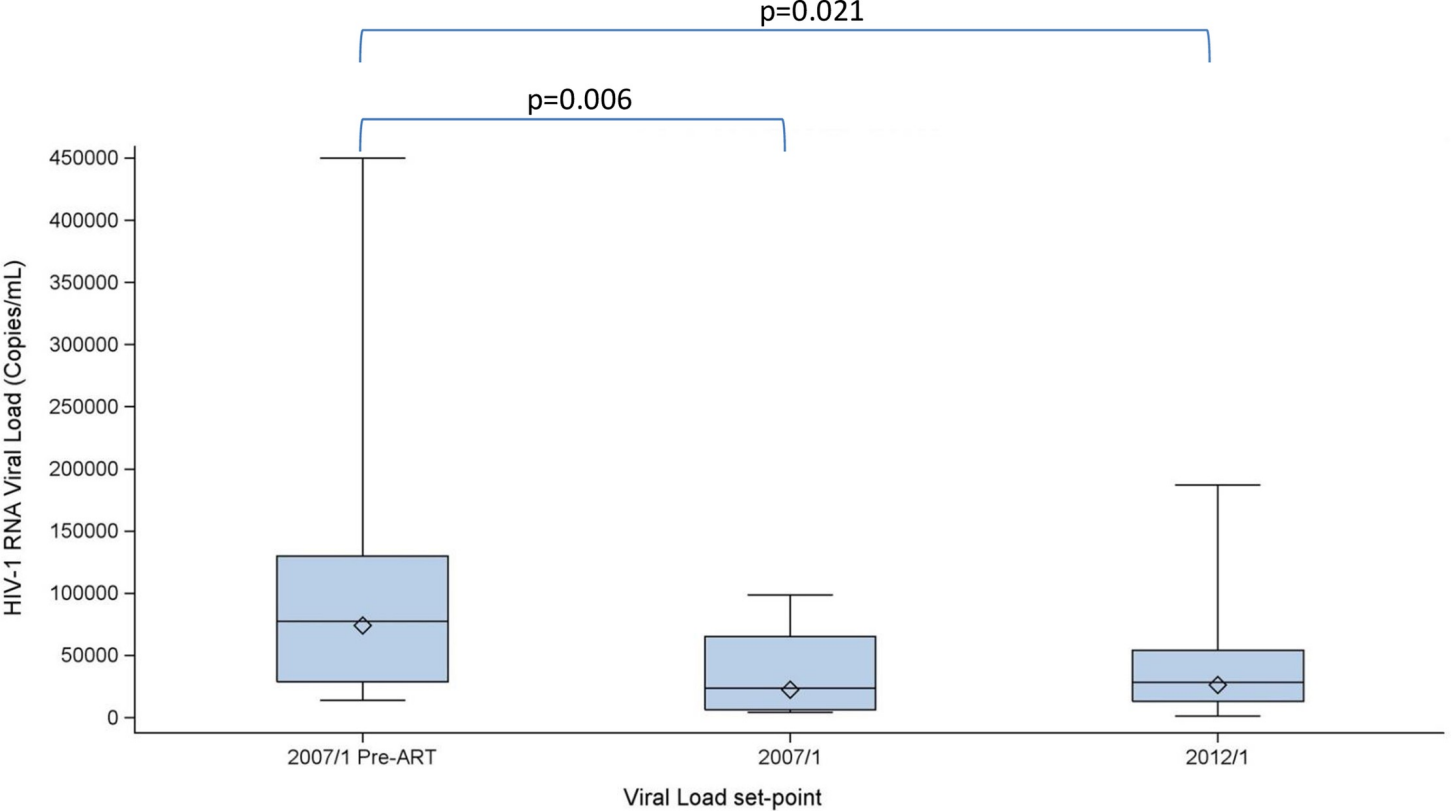
Viral load set-points in the per protocol population for participants with all three time points (n = 13). The box plots show the minimum, first quartile (Q1), median, third quartile (Q3) and maximum values for each set-point. Diamonds correspond to GM values. The maximum VL value for preART VL is 750 000 copies/mL. PreART GM VL set-point: 74048 copies/mL; 2007/1: 22475 copies/mL; 2012/1: 26279 copies/mL. The p-values are derived from a paired t-test on logged set-point values.

The VL set-points were also compared for participants that had values available for all three set-points (preART, 2007/1 and 2012/1) (Fig 3). The VL set-point established in the 2012/1 study (GM 26279) was lower than the preART set-point (GM 74048) and the reduction was statistically significant (p = 0.021, n = 13). The set-point values for these participant’s 2007/1 study set-point (GM 22475) also remained significantly lower than their preART values (p = 0.006, n = 13).

Three participants in the PP group achieved VL set-point values (mean of weeks 24 and 28) below 2000 copies/mL (1640 copies/mL, 1570 copies/mL and 1144 copies/mL). Their corresponding VL set-points (mean of weeks 48 and 52) in the 2007/1 study were 23500 copies/mL, 3830 copies/mL, and 6175 copies/mL respectively showing that their VL set-point was reduced further compared to the 2007/1 study.

**Fig 4:**
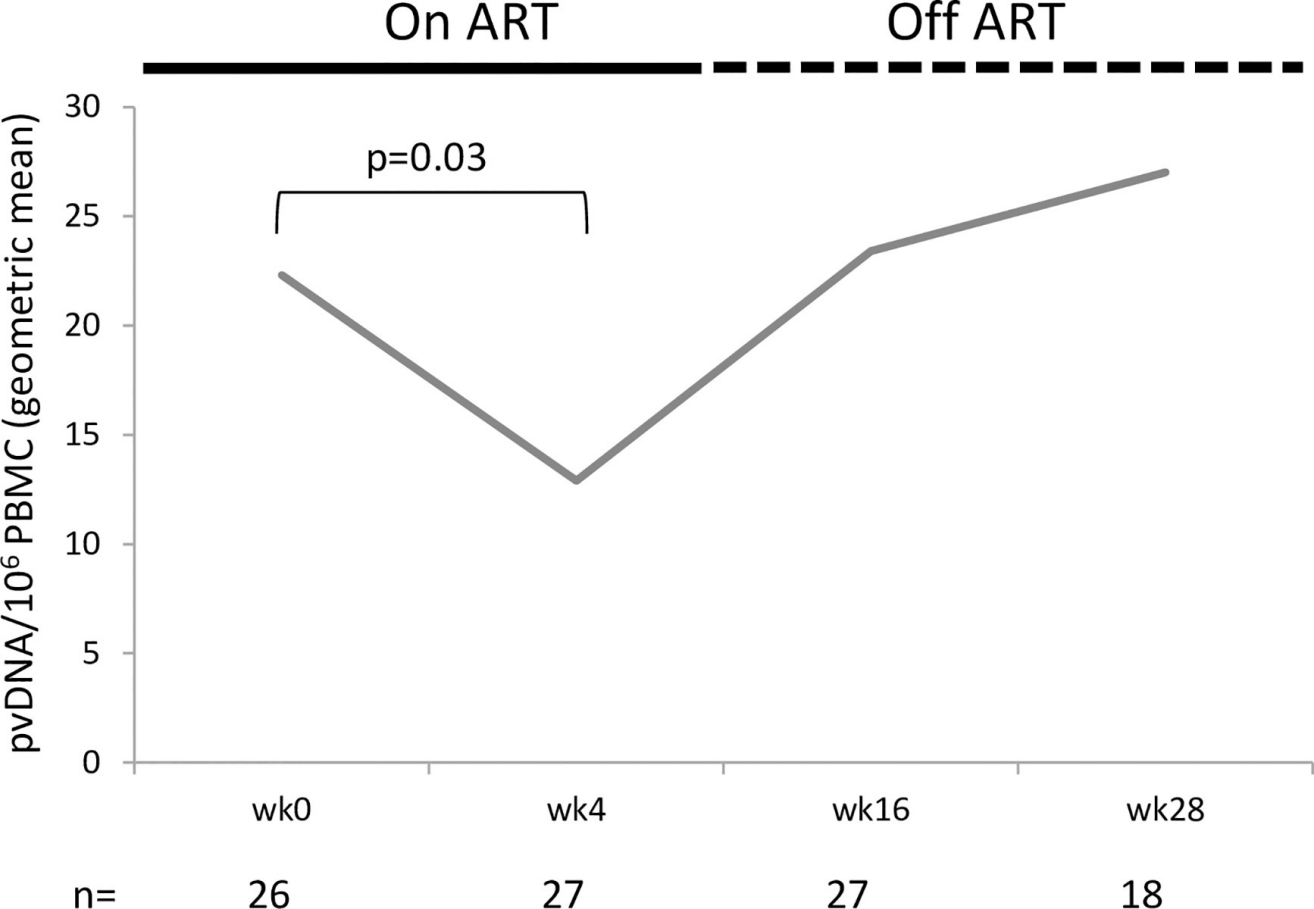
Geometric mean of proviral DNA (pvDNA) levels per million peripheral blood mononuclear cells (PBMC) in the per protocol population. One participant lacked pvDNA data from week 1. p-value was derived from a non-parametric Wilcoxon signed-rank test.

As shown in Fig 4, a statistically significant reduction in total proviral DNA (pvDNA) (49%) was observed between Wk0 and Wk4 in the PP population (p = 0.03, n = 26). However, in all subsequent pvDNA measurements, the levels returned close to the level recorded at baseline as participants were off ART at these time points.

The VL set-points for the three participants that remained off ART throughout the study were also assessed as shown in Table 3. For each of these participants, the single VL value measured at screening was higher than their respective 2007/1 and 2012/1 VL set-points. Nevertheless, their 2007/1 and 2012/1 VL set-points remained lower than their corresponding preART VL set-points. It is not clear whether the preART VL set-point of 900 000 copies/mL actually reflects part of the peak VL associated with primary HIV infection.

**Table 3:** Viral load in participants that had been off ART since the 2007/1 study and remained off ART throughout the 2012/1 study.

Participant	PreART VL (copies/mL)	2007 Mean VL set-point (copies/mL)wks 48 and 52	2012/1 VL at screening(copies/mL)	2012/1 Mean VL (copies/mL) wks 24 and 28
	PreART CD4 (cells per μL)	2007/1 mean CD4 (cells per μL)	2012/1 CD4 screening (cells per μL)	2012/1 mean CD4 (cells per μL)
**R-001-01**	70 000* (VL)	285 (VL)	2 590 (VL)	5 820 (VL)
	410 (CD4)	1019 (CD4)	670 (CD4)	626 (CD4)
**R-005-05**	190 000* (VL)	12 515 (VL)	40 900 (VL)	75 150 (VL)
	400 (CD4)	611 (CD4)	588 (CD4)	609 (CD4)
**R-006-02**	900 000* (VL)	184 (VL)	17 400 (VL)	26 700 (VL)
	375 (CD4)	1074 (CD4)	636 (CD4)	746 (CD4)

VL set-point for preART values in the 2007/1 study was based on the mean of the last two VL measurements within 6 months before ART initiation for the first time. However, for participants in Table 3, the preART values were based on a single measurement (*) either because ART was initiated shortly thereafter (R-001-01, R-006-02) or the second value was >6-months prior to ART initiation (R-005-05). Mean VL and CD4 values are given for weeks 48 and 52 for the 2007/1 study and weeks 24 and 28 for the 2012/1 study for each participant.

### Immunogenicity

#### Interferon (IFN)-γ ELISPOT

**Fig 5:**
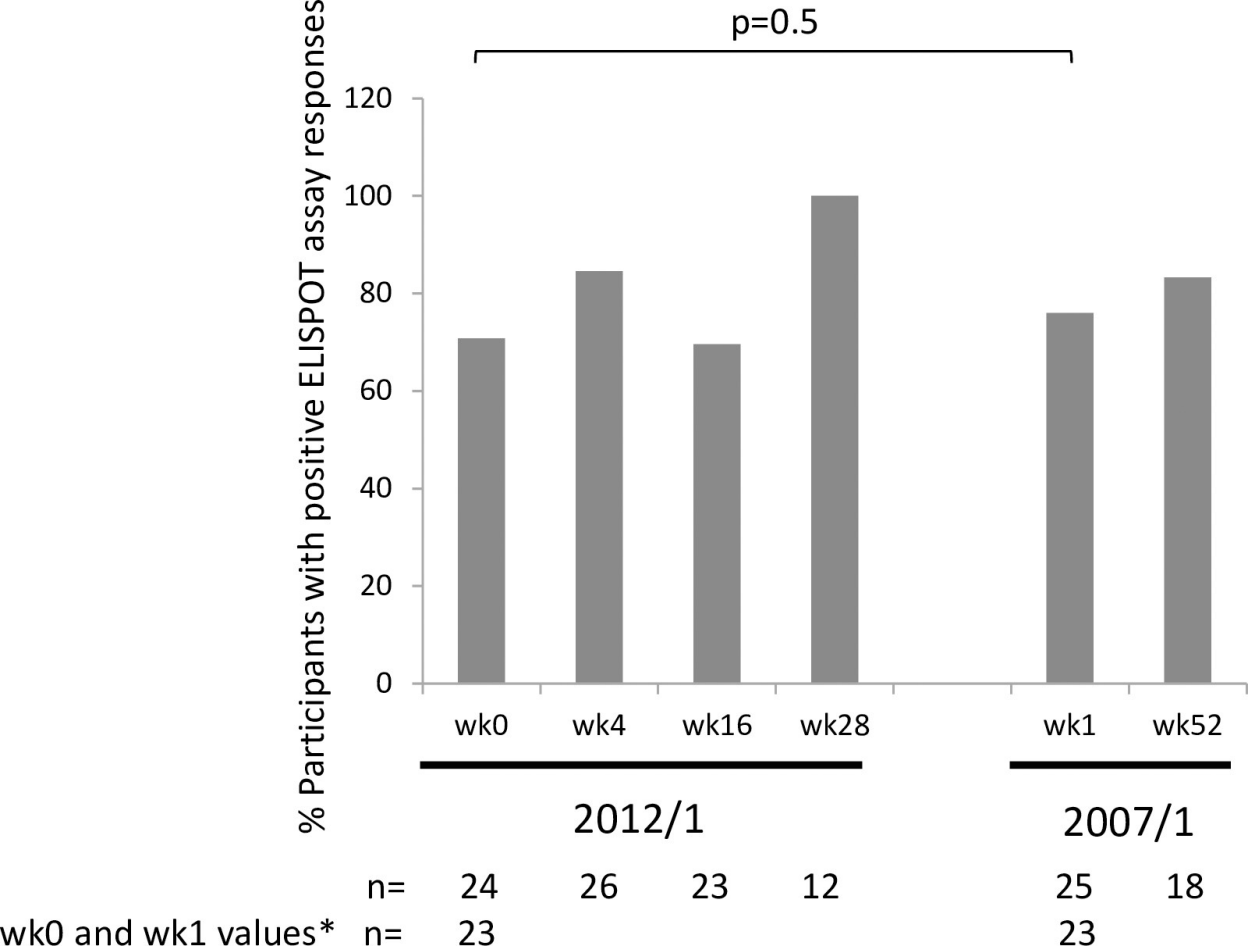
The proportion of per protocol participants showing positive ELISPOT assay responses during the 2012/1 and the 2007/1 studies. For participants with values at week (wk) 0 in the 2012/1 and wk1 in the 2007/1 study (n = 23), the paired difference in proportions of participants with positive assay response was not statistically significant (-0.87; CI: -0.202, 0.028; p = 0.5). The two-sided p-value was derived from exact version of McNemar’s test. The 95% CI may not be valid as analysis is based on a small sample of data. The sample size was too small for a comparison of ELISPOT assay responses between wk28 in the 2012/1 study and wk52 in the 2007/1 study in participants that had data for both time points (n = 11). *Participants contributing data to the p-value.

As for the 2007/1 study, a high proportion of participants in the 2012/1 study had positive IFN-γ ELISPOT assay responses at baseline (Fig 5). Furthermore, there was no difference between the proportion of positive assay responses in the 2012/1 at wk0 compared to the 2007/1 study wk1 (p = 0.5).

**Fig 6:**
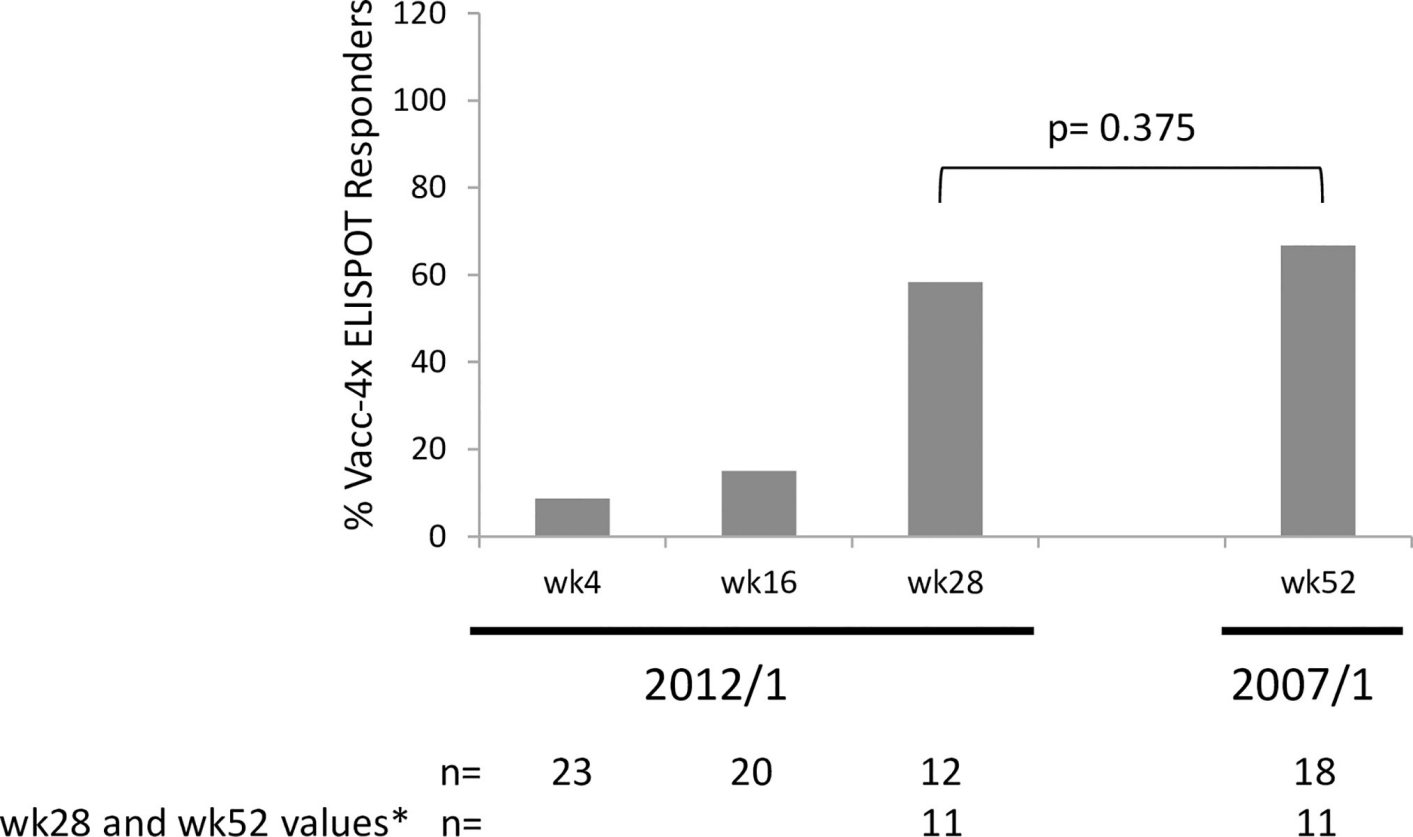
The proportion of per protocol participants showing ‘Vacc-4x ELISPOT Responder’ profile during the 2012/1 study and at week (wk) 52 for the 2007/1 study. The difference in the proportion of participants with ‘Vacc-4x ELISPOT Responder’ profile at wk0 in the 2012/1 and wk1 in the 2007/1 study (n = 11) was not statistically significant (-0.429, CI:-0.637, 0.092, p = 0.375). Two-sided p-value was derived from exact version of McNemar’s test. The 95% CI may not be valid as analysis is based on a small sample of data. *Participants contributing data to the p-value.

The proportion of participants that showed ‘Vacc-4x ELISPOT Responder’ profile in the 2012/1 study (i.e. showing a ≥ 2-fold increase in assay responses compared to baseline) was low but increased over time. The proportion of ‘Vacc-4x ELISPOT Responders’ at wk28 was not significantly different compared to wk52 in the 2007/1 study (p = 0.375) (Fig 6). The low proportion of participants with a ‘Vacc-4x ELISPOT Responder’ profile may be due to the high baseline levels of ELISPOT assay responses, limiting the potential to enhance these responses ≥2-fold at later time points. High baseline ELISPOT assay positivity was also observed in the 2007/1 study [29].

#### T-cell proliferation in CD4+ and CD8+ T-cell subsets

**Fig 7:**
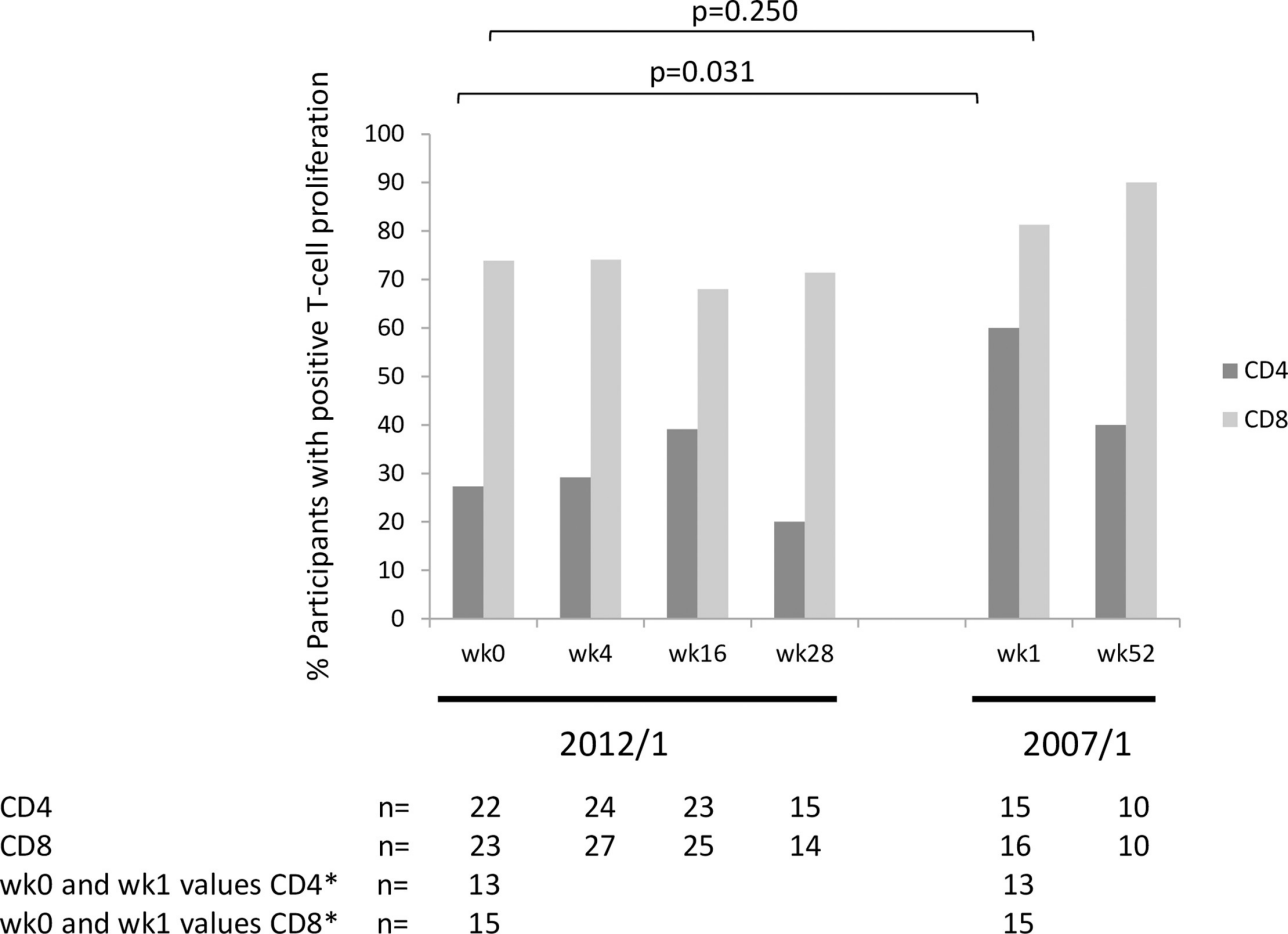
The proportion of per protocol participants showing positive T-cell proliferation assay responses during the 2012/1 study at week (wk)1 and wk52 in the 2007/1 study. For participants with values at wk0 in the 2012/1 and wk1 in the 2007/1 study, the difference in the proportion of participants with positive assay responses for the CD4 T-cell population was statistically significant (-0.462; CI: -0.733, -0.191, p = 0.031 n = 13), but not for the proportion of participants with CD8 T-cell proliferation responses (-0.200, CI: -0.420, 0.002, p = 0.250, n = 15). The two-sided p-value was derived from exact version of McNemar’s test. The 95% CI may not be valid as analysis is based on a small sample of data. The sample size was too small for a comparison of T-cell proliferation assay responses between wk28 in the 2012/1 study and wk52 in the 2007/1 study in participants that had data for both time points (CD4 n = 7; CD8 n = 7). *Participants contributing data to the p-value.

T-cell proliferative responses in CD4 and CD8 T-cell populations are shown in Fig 7. There were higher levels of T-cell proliferation in the CD8 T-cell subpopulation compared to CD4 T-cell subpopulation which was also observed in the 2007/1 study.

There was a significant difference in the proportion of participants with CD4 T-cell proliferation at wk0 in the 2012/1 study and wk1 in the 2007/1 study (p = 0.03). However, this was based on data from 13 participants. The low number of data points makes it difficult to draw firm conclusions.

**Fig 8:**
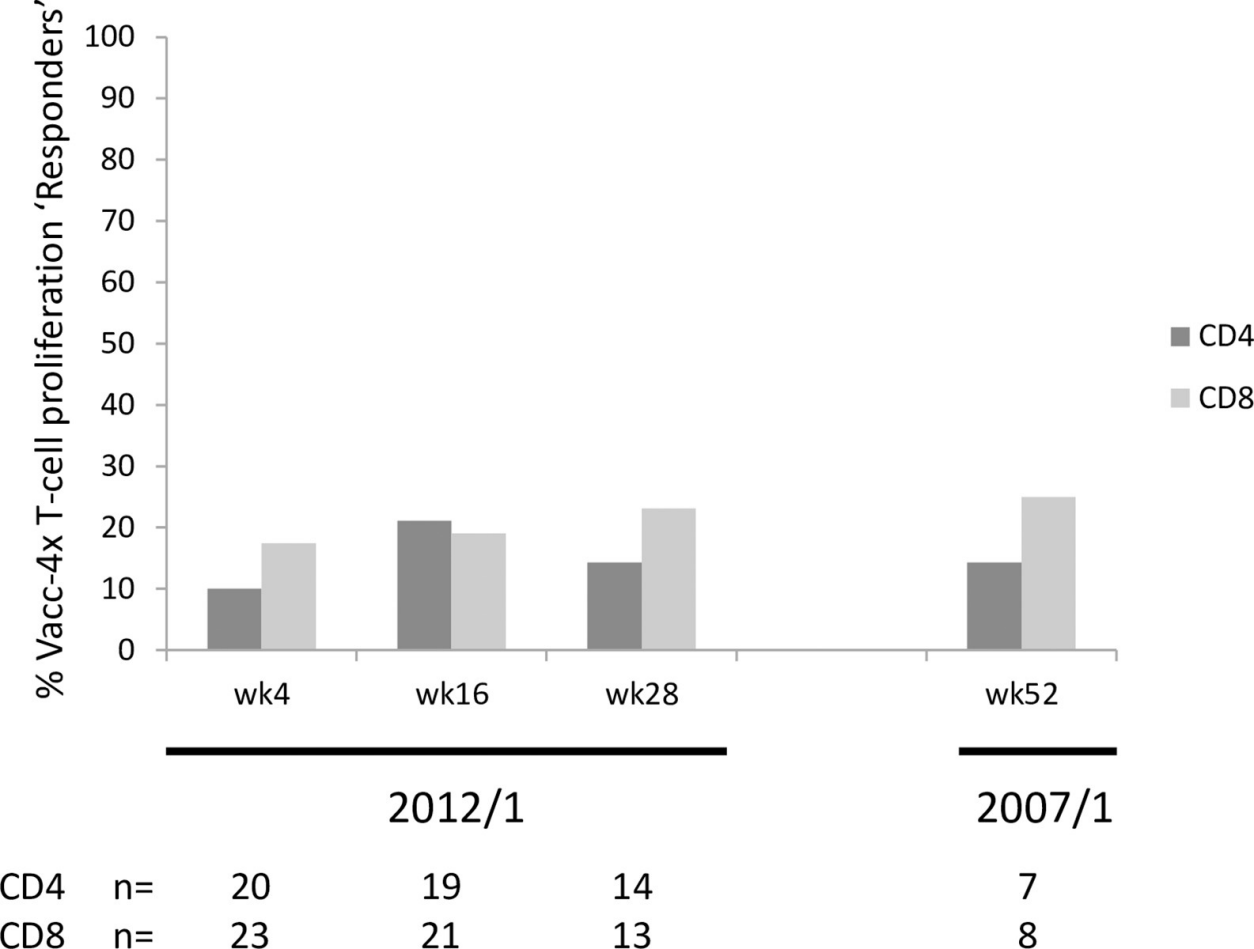
The proportion of per protocol participants showing ‘Vacc-4x T-cell proliferation Responder’ profiles in CD4 and CD8 T-cell subpopulations for the 2012/1 study and wk52 of the 2007/1 study. The sample size was too small for a comparison of ‘Vacc-4x T-cell proliferation Responders’ between wk28 in the 2012/1 study and wk52 in the 2007/1 study in participants that had data for both time points (CD4 n = 6; CD8 n = 7).

The proportion of participants showing ‘Vacc-4x T-cell proliferation Responder’ profile (i.e. increased assay responses from baseline) was low for both CD4 and CD8 T-cell subpopulations, potentially due to the challenge of increasing responses above that achieved at baseline Fig 8 [29].

#### DTH responses

There was a statistically significant increase in the proportion of positive DTH responses (induration) from Wk0 to wk4 in the 2012/1 study (p = 0.006) (Table 4). For the PP population, the corresponding treatment difference was 0.370 (CI: 0.188, 0.553, p = 0.002). Also, the proportion of participants with a positive DTH induration at Week 4 in the 2012/1 study (after re-boosting) was significantly greater than at Week 18 in the 2007/1 study (the day of the last immunization) (p = 0.022) (Table 4). The corresponding treatment difference for the PP population was also statistically significant (0.308, CI: 0.075, 0.541, p = 0.039).

**Table 4:** DTH Induration for ITT population.

**Participants in 2012/1 study (ITT)**	**n (%)**	**Treatment Diff Estimate (CI) p-value**
Week 0	10/30 (33.3%)	0.333 (0.141, 0.526) p = 0.006
Week 4	20/30 (66.7%)	
**Participants 2012/1 & 2007/1 (ITT)**	**n (%)**	**Treatment Diff Estimate (CI) p-value**
Week 4 (2012/1)	19/29 (65.5%)	0.310 (0.094, 0.526) p = 0.022
Week 18 (2007/1)	10/29 (34.5%)	

Treatment difference estimate corresponds to the paired difference in proportions achieving a positive DTH induration at the time points indicated in the 2012/1 study and comparing between the 2012/1 and 2007/1 studies. A two-sided CI is provided for the paired difference in proportions. A two-sided p-value is provided from exact version of McNemar’s test. The 95% CI may not be valid as analysis is based on a small sample of data.

#### CD4 and CD8 T-cell counts over time

CD4 T-cell counts in peripheral blood decreased from when ART was stopped (wk12) and reached the lowest level at wk28 (PP median 521 cells/**μ**L, n = 18, reduction of median 231 cells/**μ**L from baseline). The change in CD4 T-cell count from wk12 to wk28 in the 2012/1 study was not significantly different from wk28 to wk52 in the 2007 study (PP n = 18, p = 0.253).

There was a corresponding increase in CD8 T-cell count from wk12 to wk28 (PP median 1146 cells/**μ**L, n = 18, gain of 201 cells/**μ**L from baseline) while participants were off ART, however, this increase was not statistically different from the increase in CD8 count observed in the 2007/1 study (PP, p = 0.252, n = 18).

**Fig 9:**
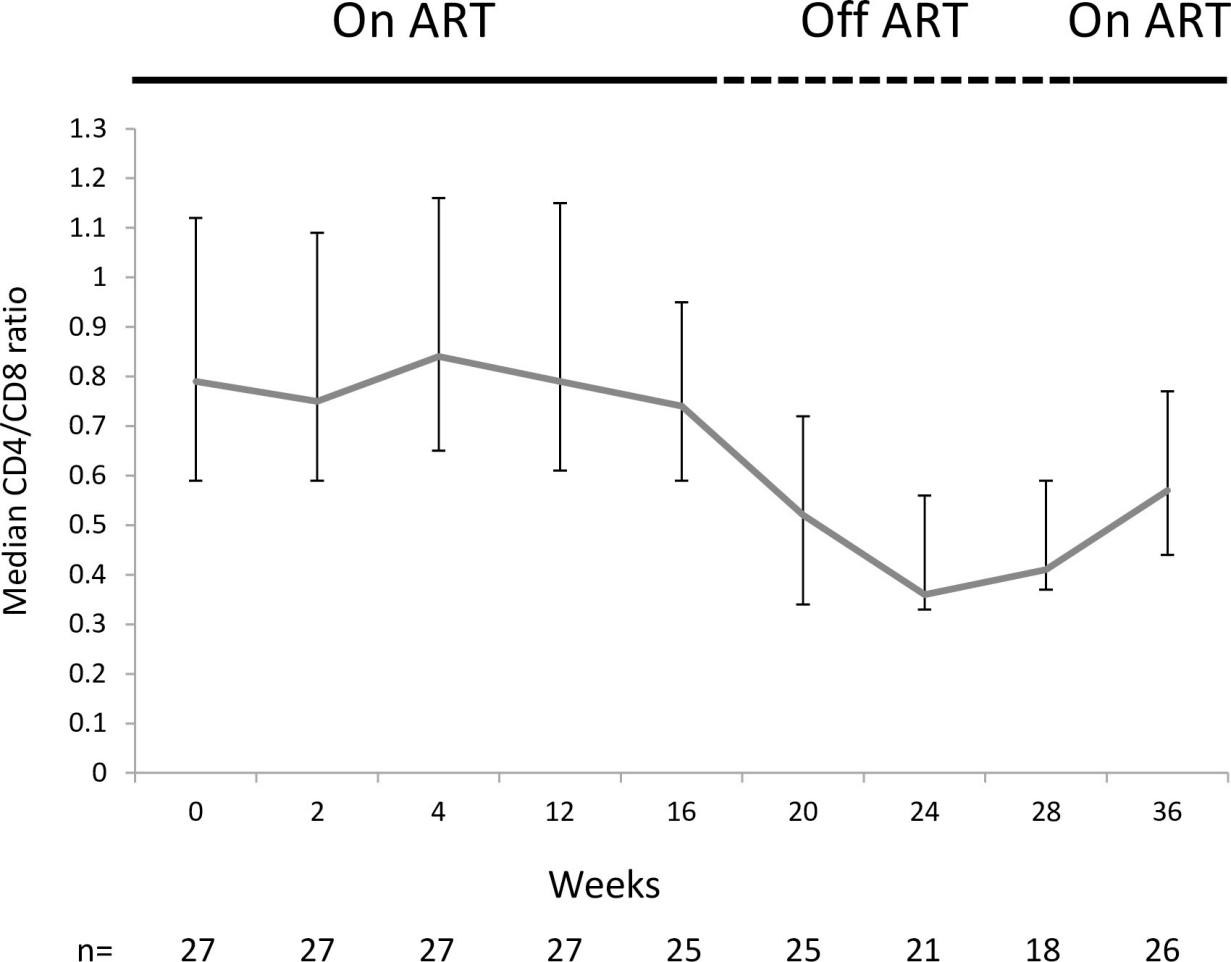
Median CD4/CD8 ratio over time for the per protocol population. ART was stopped at week 12 and resumed at week 28. Bars indicate interquartile range.

The CD4/CD8 ratio was also monitored over time. Consistent with the increase in CD8 count and the decrease in CD4 count while off ART, the CD4/CD8 ratio was seen to decrease from wk12 to wk24 (Fig 9). At wk28 (corresponding to 16 weeks off ART), participants were encouraged to resume ART and were followed for eight weeks until wk36. The CD4/CD8 ratio was shown to increase at wk36 for participants that had resumed ART.

Furthermore, there was median increase in CD4 T-cell counts of 87 cells/**μ**L from the time of ART resumption until wk36 (n = 26) in the PP population. Three participants continued off ART at wk36.

#### Adverse events

There were no deaths or adverse events resulting in withdrawal from treatment or from the study. Few adverse events were related to Vacc-4x/GM-CSF with the exception of injection site reactions. These were mainly reported as pruritus and rated as mild or moderate in intensity. An overview of treatment-related treatment-emergent adverse events (TEAE) in the safety population is shown in Table 5.

**Table 5:** Treatment–related treatment-emergent adverse events (TEAE) in the safety population.

System organ class Number (%) of participants	Vacc-4x Safety population (n = 33)
**General disorders and administration site conditions** Application site pruritus Injection site pruritus Injection site erythema Influenza-like illness Asthenia Application site reaction Injection site vesicles Fatigue Application site erythema Application site induration Chest discomfort Chills Injection site pain Injection site reaction	21 (63.6%) **16 (48.5%)** **13 (39.4%)** **4 (12.1%)** 2 (6.1%) 2 (6.1%) 2 (6.1%) 2 (6.1%) 1 (3.0%) 1 (3.0%) 1 (3.0%) 1 (3.0%) 1 (3.0%) 1 (3.0%) 1 (3.0%)
**Musculoskeletal and connective tissue disorders** Pain in extremity	1 (3.0%) 1 (3.0%)
**Nervous system** Headache Paraesthesia Aphasia Memory impairment	2 (6.1%) 1 (3.0%) 1 (3.0%) 1 (3.0%) 1 (3.0%)
**Skin and subcutaneous disorders** Hyperhidrosis Night sweats Erythema Seborrhoeic dermatitis	4 (12.1%) 2 (6.1%) 1 (3.0%) 1 (3.0%) 1 (3.0%)
**Gastrointestinal disorders** Nausea Abdominal pain	1 (3.0%) 1 (3.0%) 1 (3.0%)
**Investigations** Aspartate aminotransferase increased	1 (3.0%) 1 (3.0%)

All treatment-related TEAEs are presented. Those listed in bold correspond to >10% of participants.

## Discussion

The GM VL set-point in the PP population was not significantly lower than that obtained in the 2007/1 study (p = 0.453 n = 18). This suggests that re-boost immunizations maintained the GM VL set-point close to that obtained following primary immunization in the 2007/1 study. However, it is important to note that the treatment interruption in the 2007/1 study was 10 weeks longer than the treatment interruption in this 2012/1 study. Recently, ART-free periods in clinical studies have not generally exceeded 4 months, a margin of safety identified in the SMART study [36]. It is not known whether the VL set-point in the 2012/1 study might have been lower or similar had the ART interruption been of the same duration as for the 2007/1 study.

For PP participants that also had available preART values (n = 13), the GM VL set-point was significantly lower than their mean pre-ART VL (p = 0.021). This showed that the reduction in VL set-point compared to preART levels observed in the 2007/1 study was maintained. In the absence of any intervention, the VL set-point usually returns to preART levels [12,13]. Although the 2012/1 study was not placebo-controlled, the possibility that the observed reduction in VL set-point could be due to the phenomenon of post-treatment viral control is considered unlikely since the participant VL values were maintained at the levels observed in the prior 2007/1 clinical study, which was placebo-controlled. Interestingly, three participants achieved mean VL set-points ≤ 2000 copies/mL, also lower than their meanVL set-points in the 2007/1 study. One of these participants had a single HLA class I allele, (HLA-B57:01) linked to long-term non-progression. Earlier studies have associated VL ≤2000 copies/mL with a lower risk of transmission [37] although current objectives for functional HIV cure are that VL remains undetectable on ART interruption following therapeutic HIV immunization.

Determining viral load set-point were endpoints in both the 2007/1 and the 2012/1 studies. However, neither study was designed to evaluate the kinetics of viral rebound, and a comparison of viral load rebound is considered inappropriate because the approaches to ART interruption were different between the two studies. In the 2007/1 study, all participants were to be ART-free at week 28. As such, ART components with long half-lives were stopped early to avoid a period of ‘monotherapy’. In the 2012/1 study, ART was stopped at week 12. Furthermore, in order to assess viral recrudescence, VL measurements would have needed to be more frequent and would have included measurements of plasma ART levels following ART cessation.

For participants that had remained ART-free throughout the 2012/1 study, there was an increase in VL set-point compared to the 2007/1 study. Two of these participants likely remained ART-free in part because the VL set-point in the 2007/1 study for two of these participants was particularly low (184 copies/mL and 285 copies/mL). Nevertheless, the VL set-point was higher than the single measurement recorded at screening which could represent some level of disease progression. The potential for the rise in VL set-point to be due to immune activation induced by immunization cannot be ruled out, although another study involving intradermal immunization with a peptide-based therapeutic vaccine in ART-naïve individuals did not indicate significant increases in VL [38]. For one participant, the preART VL set-point was almost 900,000 copies/mL, however, this was based on a single value taken shortly before ART initiation and therefore potentially during initial peak viraemia following infection and before the establishment of a true set-point.

A statistically significant decrease in pvDNA levels was observed from baseline to week 4 in the PP population. Since participants had been back on ART for a mean of 36 months (min 26 max 47, n = 22) before re-boost Vacc-4x immunizations, the observed reduction in pvDNA could therefore suggest immune-based killing of infected cells following re-boosting with Vacc-4x rather than reflect the natural initial decline in pvDNA levels on ART initiation [18, 39, 40]. It is not known whether the reduced pvDNA corresponded to replication competent proviruses. This finding is nevertheless consistent with observations from the REDUC study where Vacc-4x immunization was associated with significant reductions in pvDNA (including replication competent proviral DNA) during ART, both before and after latency reversal using romidepsin [15]. pvDNA levels increased from Week 4 to Week 28 as a result of ART interruption. Other studies have similarly shown that pvDNA returns to baseline levels in the absence of ART [14]. It was not possible to compare pvDNA levels with findings from the 2007/1 study since pvDNA was not determined in the 2007/1 study. A recent randomised placebo-controlled study in newly infected individuals showed no difference in pvDNA levels over time between particpants primed with the recombinant viral vector ChAdV63.HIVconsv and bosted with MVA.HIVconsv [41]. Although immune responses were induced, there was no effect on pvDNA levels. This is in contrast to that observed to Vacc-4x. It is not clear whether the latency reversing agent in the RIVER study, vorinostat, was sufficiently effective. In the REDUC study, romidepsin was used as latency reversing agent.

In agreement with previous studies [2, 15], and irrespective of VL set-point achieved [2] or the reduction in pvDNA [15], Vacc-4x immunization did not appear to delay viral rebound which generally reached >1000 copies/mL within four to eight weeks of ART interruption. In the 2007/1 study, the time to viral rebound was not different between the Vacc-4x and placebo groups even though the VL set-point was significantly reduced compared to placebo [2]. This raises the question as to whether the factors required to delay viral rebound and the factors required to ultimately establish virus control (i.e. a set-point) below the level of detection may be different. Virus exposure during rebound could be envisaged to stimulate a broader array of innate and adaptive immune responses, which, together with more focussed T-cell responses following therapeutic vaccination, might lead to a reduction in VL set-point. It is interesting that Vacc-4x immunization appeared to reduce viral recrudescence during latency reversal in the presence of ART [15], a feature not observed during latency reversal in the absence of therapeutic vaccination [42]. This could suggest that in the presence of ART, which restricts virus spread, T-cells induced by therapeutic immunization might have been sufficient to limit virus production by targeting infected T-cells presenting viral antigens. However, the combination of Vacc-4x immunization and latency reversal was not sufficient to delay viral rebound on ART interruption. In the absence of ART, T-cell clones stimulated following therapeutic vaccination may have been insufficient to impact on virus spread and delay viral rebound. In the REDUC study, ART was resumed when VL reached 1000 copies/mL and it was therefore not possible to determine the effect of this combination intervention on VL set-point.

The high proportion of ELISPOT assay responses at baseline in the 2012/1 study may be due to either immune memory from the primary Vacc-4x immunization in the 2007/1 study and/or pre-existing immune responses to HIV. As reported earlier, the high threshold of baseline responses to HIV p24 may have impacted on the ability enhance such responses ≥2-fold to generate ‘Responders’ [29]. This may explain why the frequency of Vacc-4x ELISPOT Responders was low, but nevertheless, did increase during the course of the study. The low number of participants with ELISPOT data from wk52 in the 2007/1 study and wk28 in the 2012/1 study precluded the ability to determine any significant differences.

In common with the 2007/1 study, there was a greater proportion of participants that showed T-cell proliferation to the Vacc-4x region on p24 in the CD8 subset of T-cells, including at baseline. Although there appears to be a significantly greater proportion of participant with positive CD4 T-cell proliferation at baseline for the 2007/1 study compared to the 2012/1 study, the analysis is based on few participants and should therefore be interpreted with caution. Due to high baseline T-cell proliferation, the frequency of participants that could increase this response ≥2-fold was small but similar between the two studies.

A secondary outcome was to compare DTH induration responses between the two studies. DTH represent an in vivo measurement of immune responses to Vacc-4x antigens in the absence of GM-CSF. [2, 34, 35]. The proportion of participants, with a positive DTH response increased significantly post-baseline and compared to the 2007/1 study suggesting memory responses to Vacc-4x antigens.

As has been shown in other studies, on treatment interruption CD4 absolute counts fall, initially due to redistribution to lymphoid tissues and then due to killing by rebounding virus. CD8 T-cell counts correspondingly rose in response to rebounding virus. The CD4:CD8 ratio was therefore seen to decline while off ART. However, the changes in CD4 and CD8 T-cell counts in the 2012/1 were not significantly different between the two studies. At wk36 for participants that had resumed ART, there was an increase in median CD4 T-cell counts as well as the CD4:CD8 ratio indicating that T-cell counts were starting to recover.

Very few therapeutic vaccine studies have addressed the potential for re-boosting to maintain and/or improve vaccine effect. This may be partly because the reductions in VL set-point, or the delay in viral rebound have been insufficient alone to induce viral control/functional HIV cure. Therapeutic vaccines are therefore being assessed in combination with other interventions to improve the potential to achieve functional HIV cure. Even as a component of a functional cure, it is important to assess the duration of immune responses induced following therapeutic vaccination and the potential for reboosting to maintain therapeutic effect. One study involving a recombinant vaccinia virus expressing gp160 did assess boosting with recombinant gp160 protein one year later. Despite increases in the level of anti-Env antibodies, CTL responses were not induced. The study concluded that an alternate immunization regime may be required to maintain long-term immunity using this approach [31].

This study has a number of limitations. There was no placebo control. The participant population was a small sample of the initial clinical trial, and data were obtained for less than half of the theoretically eligible participants from the initial trial. Both issues could introduce bias. The findings should therefore be treated with caution. Ad hoc analysis of the 2007/1 baseline characteristics of the fifty-three participants that did not participate in the 2012/1 study were compared to those of the thirty-three that did participate in the 2012/1 study (S4 Table). The following characteristics that were considered to potentially influence viral rebound were median CD4 nadir [300 (200–774); n = 53 versus 296 (201–454); n = 33]; median preART CD4 [370 (200–1396) n = 46 versus 307 (201–548) n = 28]; 2007/1 median baseline CD4 [773 (342–1387) n = 52 versus 737 (456–1458) n = 33] and median VL set-point achieved in the 2007 study [26775 (36–33800) n = 44 versus 20500 (184–257500) n = 25]. The median preART VL was lower for those that did not participate in the 2012/1 study [71307 (120–2500000) n = 40 versus 110300 (14185–900000) n = 25].

The duration of treatment interruption in the 2012/1 study was shorter than or the 2007/1 study making the comparison inexact. All virological analyses were carried out in peripheral blood such that the effect of re-boost Vacc-4x immunization on pvDNA levels in tissues remains to be determined.

In conclusion, re-boosting with Vacc-4x was found to be safe and well tolerated. The study suggests that Vacc-4x re-boosting has caused a change in virus control because the post-immunization set-point, was maintained significantly lower than preART levels. However, further placebo-controlled studies will be required to address this further.

These findings nevertheless support the concept that therapeutic vaccination could become an immunological component of future ART intensification strategies [19] as well as strategies towards future functional HIV cure which in turn may help end the HIV/AIDS pandemic [43].

## Supporting information

S1 Table The CONSORT check list. (PDF)

S2 Table List of regional ethics boards that approved the CTBI-Vacc-4x 2012/1 clinical study. (PDF)

S3 Table ART regimens at the start of the 2012/1 study. (PDF)

S4 Table Comparison of participants that enrolled in the 2012/1 study and the remaining potentially eligible participants that received all Vacc-4x immunizations and stopped ART at week 28 in the 2007/1 study, but did not respond to the invitation to participate in the 2012/1 study. (PDF)

S1 File Re-boosting of subjects previously included in the CT BI-Vacc-4x 2007/1 study. **An Open, Multicenter, Immunogenicity, Follow-up Re-boosting Study with Vacc-4x in Subjects Infected with HIV-1 Who Have Maintained an Adequate Response to ART.** The CTBI-Vacc-4x-2012/1 Study protocol.
(PDF)
