# Supplementary material for: Re-boost immunizations with the peptide-based therapeutic HIV vaccine, Vacc-4x, restores geometric mean viral load set-point during treatment interruption
Source: PLoS One. 2019 Jan 30;14(1):e0210965. doi: 10.1371/journal.pone.0210965 (PMC6353572; doi:10.1371/journal.pone.0210965)
Supplement: S2 Table — (PDF) [file pone.0210965.s002.pdf]

Supporting Information

S2 Table: List of Regional Ethics Boards that approved the study.

Centre and Principal Investigator	Ethics Committee/Institutional Review Board
EPIMED<br>Gesellschaft für epidemiologische und Klinische Forschung in der Medizin mbh<br>Rubensstrasse 125<br>12157 Berlin<br>GERMANY<br>**Principal Investigator:** Dr. K. Arastéh	Ethik-Kommission des Landes Berlin<br>Landesamt für Gesundheit und Soziales Berlin<br>Geschäftsstelle der Ethik-Kommission des Landes Berlin<br>Fehrbelliner Platz 1<br>10707 Berlin
Universitätsklinikum Bonn;<br>Medizinische Klinik und Poliklinik I;<br>Immunologische Ambulanz,<br>Siegmund-Freud-Str. 25,<br>53127 Bonn<br>GERMANY<br>**Principal Investigator:** Prof J. Rockstroh	Ethik-Kommission an der Medizinischen Fakultät der Rheinischen Friedrich-Wilhelms-Universität Bonn<br>Biomedizinisches Zentrum<br>Sigmund-Freud-Str. 25<br>53105 Bonn
Universitätsklinikum Hamburg Eppendorf,<br>Ambulanzzentrum Infektiologie Haus 28,<br>Martinistr. 52,<br>20246 Hamburg<br>GERMANY<br>**Principal Investigator:** Dr J. van Lunzen	Ethik-Kommission der Ärztekammer Hamburg<br>Humboldtstr. 67 a<br>22083 Hamburg
Ospedale San Raffaele - Turro<br>Dipartimento Malattie Infettive e Tropicali<br>Via Stamira D'Ancona 20<br>20127 Milano<br>ITALY<br>**Principal Investigator:** Dr A. Lazzarin	Comitato Etico<br>Ospedale S. Raffaele s.r.l.<br>Via Olgettina 60<br>20132 Milano
Hospital Germans Trias i Pujol,<br>Carretera Canyet s/n,<br>Unidad de HIV,<br>08916 Badalona<br>SPAIN<br>**Principal Investigator:** Dr B. Clotet	CEIC Hospital Universitari Germans Trias i Pujol<br>Ctra. Canyet, s/n<br>Planta Baja - Edificio Maternal - Pasillo Archivos<br>08916 Badalona (Barcelona)
Hospital de Bellvitge<br>Unitat de VIH<br>Antiga Escola Infermeria-3ª planta<br>Feixa Llarga, s/n<br>08907 Hospitalet de Llobregat (Barcelona)<br>SPAIN<br>**Principal Investigator:** Dr. D. Podzamczer	CEIC Hospital Universitari de Bellvitge<br>Edifici de Suport a la Recerca<br>Feixa Llarga, s/n<br>08907 Hospitalet de Llobregat<br>Barcelona
Harrison Wing, St Thomas' Hospital,<br>Lambeth Palace Road<br>London SE1 7EH<br>UNITED KINGDOM<br>**Principal Investigator:** Dr. B. Peters	NRES Committee London - London Bridge<br>Health Research Authority<br>Skipton House<br>80 London Road<br>London<br>SE1 6LH

Division of Infectious Diseases<br>1500 21st St., 2nd Fl. CARES Clinic<br>CA 95811<br>Sacramento<br>USA<br>**Principal Investigator:** Dr. D. Asmuth	University of California, Davis IRB Administration<br>CTSC Bldg, Suite 1400, Rm. 1429<br>2921 Stockton Blvd<br>Sacramento, CA 95817
UCLA CARE Center,<br>Suite 980,<br>1399 S. Roxbury Drive,<br>Ste 100 Los Angeles,<br>CA 90035,<br>USA<br>**Principal Investigator:** Dr R. Mitsuyasu	UCLA Institutional Review Board (UCLA IRB)<br>University of California Los Angeles<br>11000 Kinross Avenue, Suite 211<br>Los Angeles, CA 90095-1694
