# Supplementary material for: Re-boost immunizations with the peptide-based therapeutic HIV vaccine, Vacc-4x, restores geometric mean viral load set-point during treatment interruption
Source: PLoS One. 2019 Jan 30;14(1):e0210965. doi: 10.1371/journal.pone.0210965 (PMC6353572; doi:10.1371/journal.pone.0210965)
Supplement: S3 Table — (PDF) [file pone.0210965.s003.pdf]

Supporting Information

S3 Table: ART Regimens at the start of the 2012/1 study.

ID 2012/1	Antiretroviral therapy regimen
R-001-02	ABACAVIR SULFATE W/LAMIVUDINE, RALTEGRAVIR
R-001-03	ABACAVIR SULFATE W/LAMIVUDINE, NEVIRAPINE
R-002-02	DARUNAVIR ETHANOLATE, RITONAVIR, TRUVADA (FTC/TENOFOVIR)
R-004-01	ATRIPLA (FTC/TENOFOVIR/EFAVIRENZ)
R-004-02	ATAZANAVIR, RITONAVIR, TRUVADA (FTC/TENOFOVIR)
R-004-04	NEVIRAPINE, TRUVADA (FTC/TENOFOVIR)
R-004-05	ATAZANAVIR, RITONAVIR, TRUVADA (FTC/TENOFOVIR)
R-005-03	MARAVIROC, TRUVADA (FTC/TENOFOVIR)
R-006-01	NEVIRAPINE, TRUVADA (FTC/TENOFOVIR)
R-006-03	ABACAVIR SULFATE, NEVIRAPINE
R-006-04	TRUVADA, DARUNAVIR ETHANOLATE, RITONAVIR
R-006-05	NEVIRAPINE, TRUVADA (FTC/TENOFOVIR)
R-007-01	NEVIRAPINE, ETRAVIRINE, TRUVADA (FTC/TENOFOVIR)
R-007-02	EFAVIRENZ, ABACAVIR SULFATE W/LAMIVUDINE
R-007-03	MARAVIROC, RALTEGRAVIR POTASSIUM, TENOFOVIR DISOPROXIL FUMARATE
R-007-04	RALTEGRAVIR POTASSIUM, TRUVADA (FTC/TENOFOVIR)
R-008-01	ATAZANAVIR, RITONAVIR, TRUVADA (FTC/TENOFOVIR)
R-008-02	ATAZANAVIR, RITONAVIR, TRUVADA (FTC/TENOFOVIR)
R-008-03	ATAZANAVIR, RITONAVIR, TRUVADA (FTC/TENOFOVIR)
R-009-01	MARAVIROC, RITONAVIR, DARUNAVIR ETHANOLATE
R-009-02	MARAVIROC, TRUVADA (FTC/TENOFOVIR)
R-009-03	NEVIRAPINE, TRUVADA (FTC/TENOFOVIR)
R-009-04	ABACAVIR SULFATE W/LAMIVUDINE, ATAZANAVIR SULFATE, RITONAVIR
R-009-05	ABACAVIR SULFATE W/LAMIVUDINE, MARAVIROC
R-011-01	TENOFOVIR DISOPROXIL FUMARATE, ABACAVIR SULFATE W/LAMIVUDINE, RALTEGRAVIR
R-011-02	ATRIPLA (FTC/TENOFOVIR/EFAVIRENZ)
R-011-03	ATRIPLA (FTC/TENOFOVIR/EFAVIRENZ)
