# Supplementary material for: Re-boost immunizations with the peptide-based therapeutic HIV vaccine, Vacc-4x, restores geometric mean viral load set-point during treatment interruption
Source: PLoS One. 2019 Jan 30;14(1):e0210965. doi: 10.1371/journal.pone.0210965 (PMC6353572; doi:10.1371/journal.pone.0210965)
Supplement: S4 Table — (PDF) [file pone.0210965.s004.pdf]

Supporting Information

S4 Table: Comparison of participants that enrolled in the 2012/1 study and the remaining potentially eligible participants that received all Vacc-4x immunizations and stopped ART at week 28 in the 2007/1 study, but did not respond to the invitation to participate in the 2012/1 study.

**Baseline characteristics in the 2007/1 study:**	**Enrolled 2012/1 n=33**	**Remaining 2007/1 n=53**
	**Median (min-max)**	
**CD4 nadir cells/µL**	296(201-454)	300(200-774)
**PreART CD4 cells/µL**	307(201-548) n=28	370 (200-1396) n=46
**PreART VL copies/mL**	110300 (14185-900000) n=25	71307 (120-2500000) n=40
**Baseline CD4 cells/µL**	737(456-1478)	773(342-1387) n=52
**2007/1 VL set-point copies/mL**	18900 (184-257500) n=25	23250 (36-313725) n=34

Viral load set point was defined as the mean of the last two viral load measurements before ART resumption after wk40 in the 2007/1 study (i.e. 12 weeks off ART). If participants resumed ART at week 40 in the 2007/1 study, the single wk40 measurement was taken as the set-point. If ART was resumed before wk40, no set-point was achieved because this was considered the time period when participants would experience peak viral rebound.
