# Supplementary material for: Re-boost immunizations with the peptide-based therapeutic HIV vaccine, Vacc-4x, restores geometric mean viral load set-point during treatment interruption
Source: PLoS One. 2019 Jan 30;14(1):e0210965. doi: 10.1371/journal.pone.0210965 (PMC6353572; doi:10.1371/journal.pone.0210965)
Supplement: S1 File — An Open, Multicenter, Immunogenicity, Follow-up Re-boosting Study with Vacc-4x in Subjects Infected with HIV-1 Who Have Maintained an Adequate Response to ART. The CTBI-Vacc-4x-2012/1 Study protocol. (PDF) [file pone.0210965.s005.pdf]

# Bionor Immuno AS
# Papirkaia 8, Klosterøya
# NO-3732 Skien
# Norway


**Clinical Research Protocol**

| | |
|---|---|
| **Protocol Title:** | **Re-boosting of Subjects Previously Included in the CT BI-Vacc-4x 2007/1 Study. An Open, Multicenter, Immunogenicity, Follow-up Re-boosting Study with Vacc-4x in Subjects Infected with HIV-1 Who Have Maintained an Adequate Response to ART** |

| | |
|---|---|
| **Protocol Number:** | CT-BI Vacc-4x 2012/1 |
| **Drug Product:** | Vacc-4x |
| **Drug Substance:** | A formulation of four synthetic peptides (Vacc-10, Vacc-11, Vacc-12, and Vacc-13) |
| **EudraCT Number:** | 2012-002281-12 |
| **IND Number:** | 13619 |
| **Final Protocol Date:** | 2013-08-13 |
| **Protocol Version:** | 4, Germany Specific |
| **Study Phase:** | Phase II |


**Sponsor Contact:**

| | |
|---|---|
| Name: | Vidar Wendel-Hansen MD. PhD |
| Address: | Bionor Pharma ASA<br>Kronprinsesse Märthas Plass 1<br>P.O. Box 1477 Vika<br>NO-0116 OSLO<br>NORWAY |
| Phone Number: | +47 949 85 050 |
| Fax: | +47 224 22 030 |
| Email: | vwh@bionorpharma.com |

# EMERGENCY CONTACT INFORMATION

| | |
|---|---|
| **Protocol Title:** | Re-boosting of Subjects Previously Included in the CT BI-Vacc-4x 2007/1 Study. An Open, Multicenter, Immunogenicity, Follow-up Re-boosting Study with Vacc-4x in Subjects Infected with HIV-1 Who Have Maintained an Adequate Response to ART |
| **Protocol Number:** | CT-BI Vacc-4x 2012/1 |

## In case of medical emergency please call:

**In the United States and all others Regions**:

Name: Derry Ridgway
Senior Medical Director

Address: Aptiv Solutions
3186 Leaf Drive
Merced, California 95340

Phone Number: +1 209.384.2891
Cell phone: +1 919.602.4681
Fax +1 510 655 6853

Email: derry.ridgway@aptivsolutions.com

# TABLE OF CONTENTS

Page

**PROTOCOL SIGNATURE PAGE** .......... **9**

**INVESTIGATOR PROTOCOL AGREEMENT** .......... **10**

**1 INTRODUCTION** .......... **15**

**1.1 BACKGROUND INFORMATION** .......... **15**
**1.2 INVESTIGATIONAL PRODUCT** .......... **17**
**1.3 NONCLINICAL STUDIES** .......... **18**
**1.4 CLINICAL STUDIES** .......... **18**
**1.5 RATIONALE FOR THE CURRENT STUDY** .......... **21**
1.5.1 Dose .......... 21
1.5.2 Re-boosting Schedule .......... 21
1.5.3 Use of Adjuvant GM-CSF .......... 21
**1.6 POTENTIAL RISKS AND BENEFITS** .......... **22**

**2 STUDY OBJECTIVES AND ENDPOINTS** .......... **22**

**2.1 STUDY OBJECTIVES** .......... **22**
2.1.1 Primary Objective .......... 22
2.1.2 Secondary Objectives .......... 22
**2.2 STUDY ENDPOINTS** .......... **23**
2.2.1 Efficacy Endpoints .......... 23
2.2.2 Safety Endpoints .......... 24

**3 STUDY DESIGN** .......... **24**

**3.1 OVERALL STUDY DESIGN AND FLOW CHART** .......... **24**
**3.2 RATIONALE FOR STUDY DESIGN AND TREATMENT REGIMENS** .......... **27**

**4 SELECTION AND WITHDRAWAL OF SUBJECTS** .......... **27**

**4.1 INCLUSION** .......... **27**
**4.2 INCLUSION CRITERIA** .......... **27**
**4.3 EXCLUSION CRITERIA** .......... **27**
**4.4 WITHDRAWAL OF SUBJECTS** .......... **28**
4.4.1 Vaccine Toxicity Management .......... 28

**5 TREATMENT OF SUBJECTS** .......... **29**

**5.1 STUDY DRUG AND DOSAGES** .......... **29**
5.1.1 Study Drug Description .......... 29
5.1.2 Dosage and Administration of Study Drug .......... 30
5.1.3 DTH Administration .......... 30
5.1.4 Blinding .......... 30
5.1.5 Rationale for Dosing Regimen .......... 30

5.1.6 Subject Compliance ..... 31
5.1.7 Overdose and Toxicity Management ..... 31
**5.2 CONCURRENT MEDICATIONS AND NON-DRUG THERAPIES ..... 31**
5.2.1 Permitted Medications ..... 31
5.2.2 Prohibited Medications ..... 31
**5.3 STUDY DRUG MANAGEMENT ..... 32**
5.3.1 Packaging of Study Drug ..... 32
5.3.2 Labeling of Study Drug ..... 32
5.3.3 Preparation of Study Drug ..... 32
5.3.4 Storage of Study Drug ..... 32
5.3.5 Study Drug Shipping and Handling ..... 33
5.3.6 Study Drug Accountability ..... 33
**6 STUDY PROCEDURES ..... 34**
**6.1 TIME AND EVENTS SCHEDULE ..... 34**
**6.2 SUBJECT INFORMED CONSENT ..... 34**
**6.3 PROCEDURES BY STUDY PHASE ..... 34**
6.3.1 Screening ..... 34
6.3.2 Re-boosting, DTH testing and Follow-up on ART ..... 35
6.3.3 ART-free Follow-up Period ..... 37
6.3.4 Follow-up Period to End of Study ..... 38
**6.4 SUBJECT DISCONTINUATION (LOST TO FOLLOW-UP) ..... 39**
**7 STUDY ASSESSMENTS AND MEASUREMENTS ..... 39**
**7.1 DEMOGRAPHIC AND SCREENING ASSESSMENTS ..... 39**
**7.2 EFFICACY ASSESSMENTS ..... 39**
7.2.1 Delayed-type Hypersensitivity – Test Administration ..... 39
7.2.2 CD4 and CD8 Counts ..... 40
7.2.3 HIV Viral Load ..... 40
7.2.4 Proviral HIV DNA ..... 40
7.2.5 T-cell Response ..... 40
**7.3 SAFETY ASSESSMENTS ..... 41**
7.3.1 Medical History ..... 41
7.3.2 Physical Examination ..... 41
7.3.3 Vital Signs ..... 41
7.3.4 Clinical Laboratory Tests ..... 41
7.3.5 Pregnancy ..... 42
**8 ADVERSE EVENT MANAGEMENT ..... 42**
**8.1 NON-SERIOUS AND SERIOUS ADVERSE EVENTS ..... 42**
8.1.1 Definition of an Adverse Event ..... 43

8.1.2 Definition of a Serious Adverse Event ..... 43
8.1.3 Clinical Laboratory Abnormalities and Other Abnormal Assessments ..... 44
8.1.4 Recording of Adverse Events and Serious Adverse Events ..... 44
8.1.5 Follow-up of Adverse Events and Serious Adverse Events ..... 46
8.1.6 Reporting of All Serious Adverse Events and Any Adverse Events Resulting in Study Discontinuation ..... 47
8.1.7 Post-study Adverse Events or Serious Adverse Events ..... 47
**8.2 PREGNANCY INFORMATION ..... 48**
**9 DATA COLLECTION ..... 48**
**9.1 DATA COLLECTION ..... 48**
**9.2 DATA PROCESSING ..... 49**
**10 STATISTICAL METHODS AND PLANNED ANALYSES ..... 49**
**10.1 DETERMINATION OF SAMPLE SIZE ..... 49**
**10.2 RANDOMIZATION CODES ..... 49**
**10.3 POPULATIONS TO BE ANALYZED ..... 49**
**10.4 INTERIM ANALYSIS ..... 50**
**10.5 SUBJECT ACCOUNTABILITY ..... 50**
10.5.1 Protocol Deviations ..... 50
10.5.2 Subgroup Analyses ..... 50
**10.6 STATISTICAL METHODS ..... 50**
10.6.1 Analysis of Demographic and Subject Characteristics ..... 50
10.6.2 Efficacy Analysis ..... 51
10.6.3 Safety Analyses ..... 52
**11 STUDY MANAGEMENT AND ETHICAL AND REGULATORY REQUIREMENTS ..... 53**
**11.1 REGULATORY APPROVAL AND GOOD CLINICAL PRACTICE ..... 53**
**11.2 DEVIATIONS FROM THE PROTOCOL AND PROTOCOL AMENDMENTS ..... 54**
**11.3 DISCONTINUATION OF STUDY ..... 54**
**11.4 END OF STUDY ..... 54**
**11.5 STUDY RECORDS RETENTION AND DIRECT ACCESS TO SOURCE DOCUMENTS ..... 54**
**11.6 INVESTIGATOR RESPONSIBILITIES ..... 55**
11.6.1 Subject Information and Informed Consent ..... 55
11.6.2 Institutional Review Board/Independent Ethics Committee Approval and Other Institutional Requirements ..... 56
11.6.3 Curriculum Vitae ..... 57
11.6.4 Laboratory Certification and Normal Values ..... 57
11.6.5 Delegation of Investigator Responsibilities ..... 57
11.6.6 Liability and Insurance ..... 57

11.7 STUDY MONITORING AND AUDITING ........ 57
11.8 QUALITY ASSURANCE ........ 58
11.9 STUDY TERMINATION AND SITE CLOSURE ........ 58
11.10 SITE TERMINATION ........ 59
12 DISCLOSURE OF DATA ........ 59
12.1 CONFIDENTIALITY ........ 59
12.2 PUBLICATION ........ 60
13 INVESTIGATOR'S PROTOCOL AGREEMENT ........ 60
14 REFERENCES ........ 61
15 APPENDICES ........ 64
15.1 DIVISION OF AIDS TABLE FOR GRADING THE SEVERITY OF ADULT AND PEDIATRIC ADVERSE EVENTS, VERSION 1.0 DECEMBER 2004; CLARIFICATION AUGUST 2009 ........ 64
15.2 AIDS-DEFINING ILLNESSES ........ 86

# LIST OF ABBREVIATIONS

| Abbreviation | Term |
|---|---|
| AE | Adverse Event |
| AIDS | Acquired Immunodeficiency Syndrome |
| ALT | Alanine Transaminase |
| ALP | Alkaline Phosphatase |
| ART | Antiretroviral Therapy |
| AST | Aspartate Transaminase |
| CD | Cluster of Differentiation |
| CFSE | Carboxyfluorescein Succinimidyl Ester |
| CRF | Case Report Form |
| CRP | C-reactive Protein |
| DTH | Delayed type Hypersensitivity |
| EDTA | Ethylenediamine Tetra acetic Acid |
| ELISPOT | Enzyme-linked Immunosorbent Spot Assay |
| EU | European Union |
| γ-GT | Gamma Glutamyl Transferase |
| FDA | Food and Drug Administration |
| GCP | Good Clinical Practice |
| GM-CSF | Granulocyte Macrophage Colony Stimulating Factor |
| GMP | Good Manufacturing Practice |
| HD | High Dose |
| HIV | Human Immunodeficiency Virus |
| HLA | Human Leukocyte Antigen |
| ICF | Informed Consent Form |
| ICH | International Conference on Harmonization |
| IEC | Independent Ethics Committee |
| IMP | Investigational Medicinal Product |
| IND | Investigational New Drug |
| IRB | Institutional Review Board |
| ITT | Intention-to-Treat |
| LD | Low Dose |
| LDH | Lactate Dehydrogenase |
| MedDRA | Medical Dictionary for Regulatory Activities Terminology |
| PBMC | Peripheral Blood Mononuclear Cells |
| Ph. Eur. | European Pharmacopoeia |
| PP | Per Protocol |
| Rhu-GM-CSF | Recombinant Human Granulocyte Macrophage Colony Stimulating Factor |
| RNA | Ribonucleic Acid |

| **Abbreviation** | **Term** |
|---|---|
| SAE | Serious Adverse Event |
| SAP | Statistical Analysis Plan |
| SD | Standard Deviation |
| SOC | System Organ Class |
| Th1 | T-helper Cell Type 1 |
| Th2 | T-helper Cell Type 2 |
| UK | United Kingdom |
| ULN | Upper Limit of Normal |
| US | United States |
| USP | United States Pharmacopoeia |
| WBC | White Blood Cells |
| WFI | Water For Injection |
| WHO | World Health Organization |

**PROTOCOL SIGNATURE PAGE**

**Protocol Title:** Re-boosting of Subjects Previously Included in the CT BI-Vacc-4x 2007/1 Study. An Open, Multicenter, Immunogenicity, Follow-up Re-boosting Study with Vacc-4x in Subjects Infected with HIV-1 Who Have Maintained an Adequate Response to ART

**Protocol Number:** CT-BI Vacc-4x 2012/1

**Authorized Sponsor Representative Signature** Date

Name: Vidar Wendel-Hansen MD, PhD, EVP, Head of Development
Address: Bionor Pharma ASA
P.O. Box 1477 Vika, NO-0116 Oslo
Norway
Phone Number: +47 949 85 050
Fax: + 47 224 22 030
Email: vwh@bionorpharma.com

**Authorized Sponsor Physician Signature** Date

Name: Vidar Wendel-Hansen MD, PhD. EVP, Head of Development
Address: Bionor Pharma ASA
P.O. Box 1477 Vika, NO-0116 Oslo
Norway
Phone Number: +47 949 85 050
Fax: + 47 224 22 030
Email: vwh@bionorpharma.com

**Coordinator Investigator Signature** Date

Name: Jürgen Rockstroh MD, PhD.
Address: Department of Internal Medicine
University Hospital Bonn
Sigmund-Freud-Str. 25
53105 Bonn-Venusberg
Germany
Phone Number: +49 228 287 16558
Fax: +49 228 287 15034
Email:

## INVESTIGATOR PROTOCOL AGREEMENT

| | |
|---|---|
| **Protocol Title:** | Re-boosting of Subjects Previously Included in the CT BI-Vacc-4x 2007/1 Study. An Open, Multicenter, Immunogenicity, Follow-up Re-boosting Study with Vacc-4x in Subjects Infected with HIV-1 Who Have Maintained an Adequate Response to ART |
| **Protocol Number:** | CT-BI Vacc-4x 2012/1 |

***By my signature, I confirm that my staff and I have carefully read and understand this protocol or protocol amendment, and agree to comply with the conduct and terms of the study specified herein and with any other study conduct procedures provided by Sponsor (or designee). For protocol amendments, I agree not to implement the amendment without agreement from the Sponsor and prior submission to and written approval (where required) from the Institutional Review Board (IRB), the Independent Ethics Committee (IEC), or their equivalent, and regulatory authority, except when necessary to eliminate an immediate hazard to the subjects, or for administrative aspects of the study (where permitted by all applicable regulatory requirements).***

Investigator’s Signature Date

Name:

Address:

# PROTOCOL SYNOPSIS

| | | | |
|---|---|---|---|
| **Protocol Title:** | Re-boosting of Subjects Previously Included in the CT BI-Vacc-4x 2007/1 Study. An Open, Multicenter, Immunogenicity, Follow-up Re-boosting Study with Vacc-4x in Subjects Infected with HIV-1 Who Have Maintained an Adequate Response to ART. | | |
| **Protocol Number:** | CT-BI Vacc-4x 2012/1 | | |
| **Name of Sponsor:** | Bionor Immuno AS | | |
| **Drug Substance:** | A formulation of four synthetic peptides (Vacc-10, Vacc-11, Vacc-12, and Vacc-13) | | |
| **Investigational Drug Product:** | Vacc-4x | | |
| **Phase of Development:** | II | **Indication:** | HIV-1 infection |
| **Study Center(s):** | Multicenter in the United States and Europe | | |
| **Objectives:** | **Primary objective:**<br>The primary objective of this study is to evaluate if re-boost with Vacc-4x could<br>a) reduce the viral load set-point, and<br>b) increase the immune response obtained following immunization with Vacc-4x in Study CT-BI Vacc-4x 2007/1 (EudraCT Number 2007-006302-13).<br><br>**Secondary objectives:**<br>• To evaluate the effect of a re-boost with Vacc-4x on CD4 counts and CD8 counts.<br>• To assess *in vivo* immunogenicity of Vacc-4x by evaluation of delayed type hypersensitivity (DTH) and to compare the DTH response to the DTH response observed in the initial study: CT-BI Vacc-4x 2007/1 (EudraCT Number 2007-006302-13).<br>• To evaluate the safety and tolerability of re-boosting with Vacc-4x. | | |
| **Study Design:** | The study is an open, multicenter follow-up re-boosting study of subjects who received active Vacc-4x in study CT-BI Vacc-4x 2007/1 (EudraCT Number 2007-006302-13). | | |
| **Planned Number of Subjects:** | Approximately 30-40 subjects. | | |
| **Subject Population:** | Human immunodeficiency virus (HIV)-positive subjects who have participated in the CT-BI Vacc-4x 2007/1 study and completed the immunization regimen with active drug (Vacc-4x) and stopped antiretroviral therapy (ART) at Week 28. | | |
| **Diagnosis and Main Criteria for Inclusion/ Enrollment:** | **Inclusion criteria:**<br>• Completed immunization regimen with Vacc-4x active and stopped ART (at week 28) in the CT-BI Vacc-4x 2007/1 study. (No re-start of ART is required.)<br>• Documented pre-study CD4 cell count $\geq 400 \times 10^6$/L.<br>• Documented pre-study viral load less than 300 000 copies/mL<br>• Signed informed consent.<br><br>**Exclusion criteria:**<br>• Reported AIDS-defining illness within the previous year.<br>• Malignant disease.<br>• On chronic treatment with immunosuppressive therapy.<br>• Unacceptable values of the hematologic and clinical chemistry parameters, as judged by the Investigator, including creatinine values > 1.5 x upper limit of normal (ULN), and AST, ALT and alkaline phosphatase values >2.5 x ULN.<br>• Concurrent chronic active infection such as viral hepatitis B or C or tuberculosis.<br>• Pregnant or breastfeeding women.<br>• Women of childbearing potential not using reliable and adequate contraceptive methods (defined as: use of oral, implanted, injectable, mechanical or barrier products for the prevention of pregnancy; practicing abstinence; sterile) during the 28±2 days re-boosting period including the DTH, or sexually active male subjects with partners of childbearing potential unwilling to practice effective contraception during the 28±2 days re-boosting period including the DTH and for 12 weeks after | | |

| | |
|---|---|
| | the DTH-test.<br>• Current participation in other clinical therapeutic studies.<br>• Inability to comply with the treatment protocol, in the opinion of the Investigator. |
| **Reference Product:** | None |
| **Treatment Regimen:** | Subjects will receive re-boost immunizations of 1.2 mg Vacc-4x + 0.06 mg Leukine® (sargramostim, recombinant human Granulocyte Macrophage Colony Stimulating Factor [rhu-GM-CSF], adjuvant) at Visit 2 and 3 (15±2 days between the immunizations). At Visit 5, eligible subjects whose CD4 count is ≥ $350x10^6$/L and virally controlled are expected to discontinue ART and will be followed for an additional 16 weeks without ART (at Visit 9 recommencement of ART will be recommended). |
| **Duration of Participation:** | 40 weeks; including a screening period up to 4 weeks, a 5-week re-boosting period (re-boosting and DTH at Visit 2, re-boosting at Visit 3 and DTH at Visit 4) followed by 8 weeks before cessation of ART (Visit 5), and then a 16 week ART-free follow-up period. At Visit 9, ART will be re-started and the subjects will be followed for an additional 8 weeks. |
| **Endpoints:** | **Primary efficacy endpoints:**<br><br>• Viral load set point (mean of VL count Visit 8 and 9) for subjects discontinuing ART according to protocol and *not* resuming ART until visit 9 compared to:<br><br>- viral load set point* for subjects not resuming ART before week 52 or later in the CT-BI Vacc-4x 2007/1 study and who entered this study,<br><br>or if week 52 not reached in the previous study<br><br>- viral load set point** for subjects resuming ART at the earliest week 40 in the CT-BI Vacc-4x 2007/1 study and who entered this study.<br><br>* The VL set point was defined as the mean of VL count between week 48 and week 52 in subjects who did not resume ART.<br><br>**The VL set point was defined as the week 40 VL count for subject resuming ART week 40 or the mean of the last two VL counts prior to ART resumption for subjects resuming ART week 44 or later but before week 52.<br><br>Comparison will also be made to pre-ART viral load values, if available from the CT-BI Vacc-4x 2007/1 study.<br><br>• T-cell response will be measured by ELISPOT as well as by T-cell proliferative response assay (Flow Cytometry) and intracellular cytokine staining at Visit 2 (before re-boosting), Visit 4 (28±2 days after re-boosting), Visit 6, Visit 9 and Visit 10. These will be compared to the responses reported in the study CT-BI Vacc-4x 2007/1.<br><br>**Secondary efficacy endpoints:**<br>• CD4 counts at all visits in the study, absolute numbers of CD4 counts, and treatment emergent changes in CD4 counts from mean at Screening and Week 1.<br>• Change (and percent change) in CD4 count from Visit 5 (ART discontinued) to Visit 6, 7, 8, 9 and 10.<br>• CD8 counts at all visits in the study, absolute numbers of CD8 counts, and treatment emergent changes in CD8 counts from mean of Screening and Visit 2.<br>• Change (and percent change) in CD8 count from Visit 5 (ART discontinued) to Visit 6, 7, 8, 9 and 10.<br><br>For each individual subject both the absolute and relative changes in CD4 and CD8 values registered at Visit 5, and at Visit 9 (or the last value before reinitiated ART, if ART reinitiated before Visit 9) will be compared with the corresponding values |

| | |
|---|---|
| | registered at Week 28 (ART discontinued) and Week 52 (or last visit before ART re-initiation) in the initial study CT-BI Vacc-4x 2007/1 (EudraCT Number 2007-006302-13).<br>• DTH (both induration and erythema) at Visit 2 and Visit 4, in terms of observed areas ($mm^2$ length x height) and also in terms of a positive test, defined as an area $\geq 10mm^2$ DTH-tests, both areas and number of positive tests registered at Visit 2 will be compared with the corresponding values registered at Visit 4.<br>In addition the values for these DTH parameters at Week 5 will be compared with the DTH response reported at Week 18 in the initial study CT-BI Vacc-4x 2007/1 (EudraCT Number 2007-006302-13).<br>**Safety endpoints:**<br>• Proportion of subjects who experience similar virologic suppression 8 weeks after re-initiation of ART (Visit 10) as before ART interruption (Visit 5value).<br>• AEs, recorded continuously from Screening to Visit 10 (End of Study).<br>• Vital signs (heart rate, blood pressure) at Screening, and selected visits through Visit 10 (End of Study).<br>• Clinical laboratory evaluations (clinical chemistry, hematology) at screening, and selected visits through Visit 10 (End of Study); absolute values and treatment emergent changes from mean at Screening and Visit 2. |
| **Statistical Methods:** | All subjects who receive at least one re-boosting immunization will be included within the safety population. All safety analyses will be conducted on the safety population.<br>All subjects who receive at least one re-boosting immunization and have any post-baseline efficacy data will be included in the ITT population. Unless otherwise indicated, all efficacy analyses will be conducted on the ITT Population. All ITT subjects who receive two re-boosting immunizations discontinue ART at Week 13 (as planned) and who do not incur a major protocol violation that would challenge the validity of their data will be included within the PP population. All efficacy analyses will also be conducted on the PP population. No interim analysis is planned.<br>In general continuous variables will be described using number of observations (n), mean, standard deviation (SD), geometric mean, minimum, maximum, median, and quartiles. Categorical data will be presented using counts (n) and percentages (%). All statistical analyses will be performed using appropriate procedures in SAS Software Version 9.2 or higher. Significance of effects will be determined by two-sided tests with $p\text{-value} < 0.05$.<br>All analyses will be carried out after all participants have completed Visit 10 (or early withdrawal from study) and the study database has been authorized by the Sponsor as complete and final, and protocol deviations have been identified.<br>The primary efficacy endpoint of this study is the comparison of the re-boost viral load set-point after re-boosting and a 16 week ART-free period with the viral load set-point reported in the initial CT-BI Vacc-4x 2007/1 study (EudraCT Number 2007-006302-13). The primary comparison will be a Wilcoxon signed rank test comparing changes in viral load between the two stated comparison values. The re-boost viral load set-point will also be compared to the pre-ART viral load value, if available from the study CT-BI Vacc-4x 2007/1. The co-primary endpoint of this study is the T-cell response to Vacc-4x and/or p24 which will be measured by ELISPOT as well as by T-cell proliferative response assay (flow cytometry) and intracellular cytokine staining at Visit 2 (before re-boosting), Visit 5 (two weeks after re-boosting), Visit 6, 9 and 10 (End of Study). These responses will be compared to the T-cell response reported in the initial study CT-BI Vacc-4x 2007/1 (EudraCT Number 2007-006302-13) using McNemar's |

| | |
|---|---|
| | test. Secondary efficacy endpoints for CD4, CD8, and DTH (induration and erythema) parameters will be defined based on scheduled visit assessments post start of study medication, to be compared to results from the initial study CT-BI Vacc-4x 2007/1 (EudraCT Number 2007-006302-13). Continuous data types will be analyzed using the Wilcoxon signed rank test, binary data types using McNemar's test. Safety will be assessed through the collection of AE reports, assessing virologic suppression, vital signs, and laboratory evaluations that are commonly employed in clinical studies. Visit 2 safety data will be used for comparison with the data obtained during the study period. Summary statistics will be prepared for measured values and change from Visit 2 values. Virologic suppression will be assessed between the timing of ART interruption and ART re-initiation. Summaries of treatment-emergent clinically important abnormalities in vital signs and laboratory data will be provided. No statistical testing will be performed on safety parameters. |

# 1 INTRODUCTION

## 1.1 Background Information

Human immunodeficiency virus (HIV) infects the cluster of differentiation 4 (CD4) subset of T-cells that are critical for initiating immune responses to infection. The level of CD4 cells in the blood is a marker of a subject's immunological status. During the course of an HIV infection, the number of CD4 cells decreases, resulting in reduced immunological responsiveness and ultimately immune deficiency. Before the advent of HIV medication, it was clear that sustained immune responses to the HIV capsid protein p24 were inversely correlated with disease progression (CD4 cell decline) (Forster, 1987; Weber, 1987). When CD4 counts are below $200x10^6$/L, immune status is reduced to a level that allows for opportunistic infections. In contrast, long term non-progressor subjects show sustained immune responses to HIV p24 (Dyer, 2008). Sustained immune responses to p24 appear to be essential to maintain CD4 counts and thereby restrict disease progression.

Current management of an HIV infection includes antiretroviral therapy (ART). The advent of effective ART in 1996 led to a profound decrease in type 1 HIV (HIV-1)-associated morbidity and mortality in developed countries where ART has been available.

Despite the ability of ART to inhibit HIV-1 replication, it cannot cure infection, making ART a life-long treatment that requires sustained compliance and imposes significant individual and societal financial burdens on healthcare services. Furthermore, ART side effects (e.g., metabolic toxicity and stigmatizing body fat redistribution) often require medication that further increases the inconveniences and financial burdens of HIV management. Of additional concern is the emergence of viruses resistant to ART that can result in treatment failure.

Vacc-4x is a peptide-based HIV therapeutic vaccine. The primary objective of Vacc-4x therapeutic vaccine is to strengthen the immune system's response to HIV p24.

ART dramatically reduces the level of virus in circulation in the body, thereby allowing the immune system to focus on the therapeutic vaccine that is administered. ART also allows for the generation of new naïve CD4 cells that can be triggered by the therapeutic vaccine to generate new immune responses to HIV-1. Subjects are therefore immunized with Vacc-4x in the presence of ART to generate new HIV-specific immune responses that can sustain immunological fitness for prolonged periods when subjects are removed from ART. It is likely that periodic boosting on ART will be required to sustain the immunotherapeutic effect – in this way ART may become an intermittent therapy.

Vacc-4x is comprised of peptides that are modified from conserved domains of HIV p24, the major capsid protein of HIV. It is administered intradermally following the administration of recombinant human-granulocyte macrophage colony stimulating factor (rhu-GM-CSF), which is used as a local adjuvant. GM-CSF is specifically chosen because of its ability to facilitate dendritic cell maturation and migration to the lymph nodes for antigen presentation (Disis, 1996; Banchereau, 1998). Dendritic cells are targeted because they are the most potent antigen presenting cells of the

immune system and have the capacity to carry out 'cross presentation' during which foreign antigens are presented on both human leukocyte antigen (HLA) classes 1 and 2 (Figure 1).

**Figure 1 Simplified Diagram Showing the Two Pathways for Peptide Digestion Leading to Presentation at the Cell Surface**

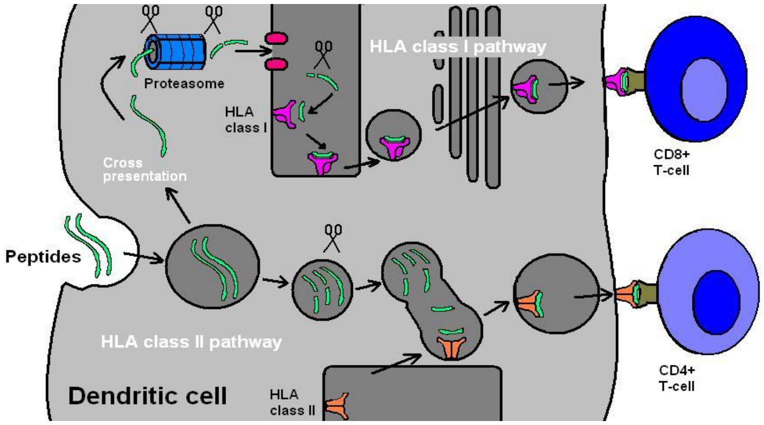


Dendritic cells ingest peptides into small vesicles. If they follow the human leukocyte antigen (HLA) class II pathway, they are cleaved within the vesicle that subsequently fuses with one carrying the HLA class II molecule. Some of the peptide fragments bind to the HLA class II and reach the cell surface attached to this molecule. The peptide fragment is recognized by a CD4 cell that docks onto the HLA molecule. Dendritic cells have the unique ability to transfer peptides to the HLA class I pathway. Here, the peptides are digested by the proteasome. The peptide fragments are then transported into specialized compartments of the cell where the HLA class I molecules reside. The peptides attach themselves to the HLA class I molecule and are transported to the cell surface. Peptide fragments are recognized by a CD8 cell that docks onto the HLA class I molecule. (From: Sommerfelt, 2004)

A consequence of dendritic cell stimulation by peptides is the proliferation of both CD4 and CD8 cells. The CD8 cells acquire the ability to recognize infected cells expressing HIV epitopes that are represented in the modified peptides. The Vacc-4x therapeutic vaccine therefore aims to stimulate cell-mediated immunity.

To date, four clinical studies have been conducted with Vacc-4x. A Phase I clinical study (Study CTN B-HIV-1/99) was performed at the University of Bergen, Norway enrolled 11 chronic HIV-infected subjects (Åsjö, 2002). A Phase II clinical study (CTN B-HIV-2/2001) performed at Ullevål University hospital in Oslo, Norway enrolled 40 chronic HIV-infected subjects. Immunization started late 2002 and the study ended in 2004 (Kran, 2004). On completion of the Phase II trial, subjects that responded well to Vacc-4x had significantly reduced viral loads and correspondingly improved CD4 counts compared to low responders at the end of a three month ART-free period (Kran, 2005). Furthermore, the majority of subjects remained off ART on completion of the study, and the duration of treatment interruption was linked to immune responsiveness to the peptides (Kran, 2006). The median treatment interruption achieved for subjects was 31 months. A re-boosting study of 22 available subjects from the Ullevål study was performed in 2010 (CTN-BI/Vacc-4x/2009/1). Long-term proliferative CD4 and CD8 T cell memory was shown 7 years after

intradermal immunizations against short HIV Gag p24-like peptides targeting dendritic cells (Lind, 2010; Lind, 2012). In addition, Vacc-4x has been tested in a double blind placebo-controlled multinational Phase II clinical study (CT-BI Vacc-4x 2007/1 - EudraCT Number 2007-006302-13). This study included 135 subjects. The study showed no difference in time to return to ART, or in CD4 counts over time between the Vacc-4x and the placebo group. However, The viral load set point after ART cessation varied between subjects with evidence of favorable effect of Vacc-4x immunization over placebo. There was also a significant difference in the viral load set point (mean of Weeks 48 and 52) compared to pre-ART values in the Vacc-4x, but not the placebo group. Preliminary immunological analyses shows that positive enzyme-linked immunosorbent spot assay (ELISPOT) responses to p24 in the Vacc-4x group were associated with a reduced viral load set point compared to placebo. This indicates a potential qualitative rather than a quantitative difference in immunological responses to p24 between the Vacc-4x and placebo groups. Vacc-4x was also found to be safe and well tolerated (Rockstroh, 2011).

## 1.2 Investigational Product

Vacc-4x is comprised of peptides that are modified from conserved domains of HIV p24, the major capsid protein of HIV. Prior to the introduction of ART, it became clear that the survival time during HIV-1 infection was related to immune responsiveness to the major capsid protein p24. When immune responses to p24 declined, disease progression was accelerated. Disease progression leading to Acquired Immunodeficiency Syndrome (AIDS) is related to CD4 counts. United States (US) and European consensus guidelines from December 2007 recommend treating subjects who have CD4 counts $\leq 350 \times 10^6$/L. Sustained immune responses to p24 should maintain CD4 counts and delay disease progression. It has been shown that p24 antigens (not virus-associated) can persist during ART. Increasing levels of p24 antigen while on ART are inversely correlated with CD4 count and disease progression. Sustained immune responses to p24 appear to be essential to maintain CD4 counts and thereby restrict disease progression. The Vacc-4x peptides are based on conserved domains of the major capsid protein p24. Vacc-4x has induced new and sustained immune responses to p24; immune responsiveness to Vacc-4x correlated with time off ART.

## 1.3 Nonclinical Studies

Conclusions from Non-clinical Safety Studies – Vacc-4x

The results of single-dose toxicity studies in mice and rats (Covance 1638/3; Covance 1638/4) indicate that intravenous Vacc-4x elicit no adverse reactions at doses up to 3 mg/kg. Thus, the no adverse effect level (NOAEL) was determined to be in excess of 3 mg/kg, which constitutes a 175-fold safety margin over the human dose level (1.2 mg peptides).

The safety of Vacc-4x and the adjuvant rhu-GM-CSF were also evaluated in a 22-week intradermal rabbit repeated dose toxicity study (Covance1638/010). Vacc-4x and rhu-GM-CSF were administered intermittently study weeks 1, 2, 3, 4, 12, 16, 18 and 22. The dosing regimen mimicked the regimen used in the clinical CT-BI Vacc-4x 2007/1 study up to study week 18. The administration study week 22 was in addition to the conducted clinical CT-BI Vacc-4x 2007/1 dosing regimen. Two different dose levels were evaluated, i.e. 0.12 mg/dose (1/10 of human dose) and 1.2 mg/dose (human dose). Local intradermal reactions such as erythema and edema were noted in Vacc-4x treated animals. However, similar effects were noted in control animals both macroscopically and histologically. The local reactions were slightly more pronounced in the Vacc-4x treated animals. There were no systemic adverse reactions in this study. These data indicate that Vacc-4x has no limiting toxicology in a model that is relevant to the proposed clinical study.

A 4 week intermittent intradermal administration toxicity study (Covance 1638/011) in the rabbit, with a 4-week treatment free period was done to bridge between the adjuvant Molgramostim (used in the Covance study number 1638/010, referred to as GMCSF) and the adjuvant Leukine® (sargramostim). In conclusion, the intradermal administration of Vacc-4x (1.2 mg/dose) combined with the adjuvant Leukine® or Molgramostim to the rabbit once weekly for 4 weeks was well tolerated except for local reactions. No systemic effects were noted in any parameter during the study. Local reactions at the repeat injection site were comparable for animals dosed with Leukine® or Molgramostim in addition to Vacc-4x.

## 1.4 Clinical Studies

The immunotherapy candidate, Vacc-4x, completed a Phase I clinical study at the University of Bergen, Norway, and a Phase II clinical study at Ullevål University hospital in Oslo, Norway, with a follow-up re-boosting study (EudraCT Number 2009-015249-22) and a Phase II clinical study (EudraCT Number 2007-006302-13) performed in US and Europe (United Kingdom [UK], Germany, Spain and Italy).

SUMMARY Phase I study (Study CTN B-HIV-1/99):

The Phase I study enrolled 11 HIV-positive subjects, including 9 subjects on ART. Subjects were maintained on ART (if entered on ART); all subjects were treated with 12 immunizations of Vacc-4x at a dose of 0.4 mg/intradermal injection over a period of 26 weeks. Immunizations were performed following injection of rhu-GM-CSF (Leucomax® [molgramostim]) as adjuvant.

*Safety Results:*
All subjects experienced one or more adverse events (AEs); nine subjects experienced events judged as related to treatment. The adverse reactions reported were mild or moderate in severity except for severe local reactions at the injection site in one subject. No subjects were withdrawn due to treatment-related AEs or toxicological reactions; no serious AEs (SAEs) occurred.

*Efficacy Results:*
All subjects experienced a cell-mediated immune response, measured by delayed-type hypersensitivity (DTH) skin reaction. Some cell-mediated immune response, measured by gamma interferon (γ-IFN) release using ELISPOT, was reported for 45% of the subjects; no antibody response to Vacc-4x peptides was observed.

SUMMARY Phase II Study (Study CTN B-HIV-2/2001):

The Phase II study (CTN B-HIV-2/2001) of Vacc-4x enrolled 40 HIV- positive subjects, of which 38 completed the trial. This was a dose-finding study where subjects were maintained on ART and treated with 10 immunizations at a dose of 0.4 mg (20 subjects) or 1.2 mg (20 subjects) of Vacc-4x intradermal injection, over a period of 26 weeks. Immunizations with Vacc-4x were performed following injection of rhu-GM-CSF (Leucomax [molgramostim]) as a local adjuvant. ART was interrupted from Week 26 to Week 30 to allow exposure to the subject's own virus (autologous immunization). ART was resumed from Week 30 to Week 38 to allow maturation of immune responses to the Vacc-4x peptides and to the subject's own virus. ART was discontinued from Week 38 to Week 52, when the study was formally concluded.

*Safety Results:*
No safety concern was raised during this study. No SAEs were reported during the period of immunization. One subject experienced a transient vasovagal reaction in conjunction with immunization and the DTH test at Week 26 and Week 38. A second subject experienced a vasovagal reaction in conjunction with the DTH test at Week 52. For the laboratory parameters, vital signs, and performance status, no changes attributable to immunization were observed. Changes in HIV ribonucleic acid (RNA), CD4 cell counts, and CD8 cell counts showed no safety concerns related to immunization.

*Efficacy Results*:
Immunological responses reported as DTH-positive reactions were observed for all subjects. Overall, positive responses both for induration and erythema were statistically significantly higher in the high dose (HD, 1.2 mg Vacc-4x) group compared to the low dose (LD, 0.4 mg Vacc-4x) group. The dose-dependent differences in DTH reactions were maintained throughout the study. T-cell proliferation appeared stable after Week 12 and demonstrated an HD advantage, consistent with the DTH results. ART was interrupted at Week 38 with planned re-start when CD4 counts fell to less than 200/µL or when AIDS- or HIV-related events were observed (i.e. clinical practice). DTH responses to Vacc-4x (high versus low response determined at Week 38) were associated with reduced viral loads and correspondingly improved CD4 counts at the end of the study (Week 52).

During the immunization period, CD4 counts were stable or increased. Interruption of ART resulted in reduction of CD4 counts. However, 14 weeks after the last interruption of ART (Week 52), the mean CD4 counts were still above 200 $x10^6$ cells/L. No difference between the LD and the HD groups was observed. The majority of subjects remained off ART following completion of the study (Week 52); permission was given to follow the subjects until they resumed ART. The duration of treatment interruption was linked to immune responsiveness to the peptides. When study subjects were compared to similar subjects in the Netherlands that had stopped treatment without immunotherapy, a significantly slower decline in CD4 cells was noted for the Vacc-4x subjects. The median treatment interruption achieved for all subjects that participated in the Vacc-4x Phase II clinical study was 31 months.

In a follow-up study of 26 of these subjects (CTN-BI/Vacc-4x/2009/1 - EudraCT Number 2009-015249-22), seven years after immunization, most subjects who earlier had responded to Vacc-4x now showed detectable CD4 memory cells which could be activated.

SUMMARY Phase II Study (CT-BI Vacc-4x 2007/1 - EudraCT Number 2007-006302-13):

This study was performed in US and Europe (UK, Germany, Spain and Italy). The study was a randomized, double-blind, multicenter, immunogenicity study of Vacc-4x versus placebo in subjects infected with HIV-1 who had maintained an adequate response to ART. The primary objective was to evaluate the effect of Vacc-4x immunizations versus placebo on CD4 counts, T-cell function (ELISPOT, T-cell proliferative responses and intracellular cytokine staining) and the response to interruption of ART. The study was planned to include approximately 345 subjects but due to recruitment difficulties the study was amended to reduce the planned number of subjects to a minimum of 120 subjects. Due to reduced numbers of subjects the Food and Drug Administration (FDA) responded as follows: “Due to the size (N=130) and nature (no formal hypothesis testing) of this study, please understand that CBER [Center for Biologics Evaluation and Research] will regard this study as exploratory and for hypothesis-generating purpose only”. Following the FDA response it was important to look more in detail into the data by including post-hoc analyses.

It was concluded that Vacc-4x did not reduce the proportion of subjects requiring resumption of ART after ART cessation at Week 28 in comparison with placebo. In addition, there was no effect compared with placebo on the percentage change in CD4 count between Week 28 and the last CD4 assessment before resumption of ART.

The time to restarting ART was similar in Vacc-4x and placebo-treated subjects.

The viral load set point (mean of the last two viral load determinations before ART resumption or at study end at week 52) varied between subjects with evidence of favorable effects of Vacc-4x immunization over placebo. In the subgroup of “completer” subjects who remained off ART until Week 52, the median viral load set point was significantly lower ($p = 0.0397$) in the Vacc-4x-treated subjects (22300 copies/ml, $n = 56$) compared to placebo-treated subjects (61900 copies/ml, $n = 25$). In the subgroup of “LOCF” (last observation carried forward), including all subjects off ART until at least Week 40, the median viral load set point was lower in the Vacc-4x group (26775

copies/ml, n = 72) compared to the placebo group (57975 copies/ml, n = 32), but this difference did not reach the level of statistical significance (p = 0.1489).

No safety concern was raised during this study. The study was supervised by a Data Safety Monitoring Board (DSMB).

## 1.5 Rationale for the Current Study

This study is a follow-up, re-boosting study of Study CT-BI Vacc-4x 2007/1 (EudraCT Number 2007-006302-13) performed in US and Europe (UK, Germany, Spain and Italy). All subjects to be included have been given a therapeutic immunization with Vacc-4x during the CT-BI Vacc-4x 2007/1 study. During the study a reduction in the viral load set-point (mean viral load at Week 48 and Week 52, or if Week 52 not reached, mean viral load of the last two measured values before restart of ART) was seen in the Vacc-4x group compared to placebo group. Further stimulation of the immune system by re-boosting with Vacc-4x could reduce the viral load set-point further. The study is open because the number of subjects available is limited.

### 1.5.1 Dose

The re-boosting dose will be 1.2 mg of Vacc-4x, the same as given in Study CT-BI Vacc-4x 2007/1 (EudraCT Number 2007-006302-13).

### 1.5.2 Re-boosting Schedule

The subjects will receive two re-boosting immunizations with a 2 weeks interval between immunizations.

### 1.5.3 Use of Adjuvant GM-CSF

Peptide vaccines are poorly immunogenic by themselves. To induce measurable levels of T-helper cell type 1 (Th1) or type 2 (Th2) immune responses against these peptides an adjuvant is often required. Recombinant humanGM-CSF was selected for the development of Vacc-4x based on its adjuvant activity when given in combination with protein and peptide antigens (Hodge, 2006; Arellano, 2004). GM-CSF facilitates dendritic cell maturation and migration to the lymph nodes for antigen presentation (Disis, 1996; Banchereau, 1998) and is indicated for other uses, such as chemotherapy and transplantations. Following chemotherapy, GM-CSF is administered subcutaneously at an initial recommended dose of 3 μg/kg/day, followed by a maintenance dose between 0.3 and 8 μg/kg/day; however, when using GM-CSF as an adjuvant, GM-CSF is administered intradermally at much lower doses (Gjertsen, 2001; Brunsvig, 2006; Bernhardt, 2006). In the Vacc-4x program, $0.33 \times 10^6$ international units (IU) GM-CSF is used (irrespective of body weight), a dose which is considered to be safe and sufficient to induce a local response. Other studies have also demonstrated the safety and potential usefulness of GM-CSF in this setting (Sasaki, 2003). GM-CSF administered subcutaneously may induce modest, but not clinically significant, increases in plasma HIV type 1 RNA levels and CD4 lymphocyte counts in subjects with uncontrolled HIV infection (Jacobson, 2003); however, in the current study, as in preceding

ones, subjects will be exposed to commercial GM-CSF (Leukine® sargramostim, rhu-GM-CSF) while on ART when viremia is controlled.

## 1.6 Potential Risks and Benefits

Participants will provide important scientific information that may contribute to improved future vaccination of HIV-positive subjects. Potential benefits for participants include a possible sustained improvement in the immune response to p24 and HIV. Potential risks include the discomfort and inconvenience associated with the immunizations and the risk of known or unknown side effects of exposure to Vacc-4x and Leukine® including, most commonly, local reactions at the site of injections and fatigue (incidence not yet determined). However, since all subjects in this study have participated in Study CT-BI Vacc-4x 2007/1 (EudraCT Number 2007-006302-13) they are aware of potential discomfort.

Interruption of ART for extended periods is associated with an increased morbidity and mortality, thus planned long-term therapy interruption strategies are not recommended outside of controlled clinical trials (Department of Health and Human Services [DHHS] Guidelines for the Use of Antiretroviral Agents in HIV-infected Adults and Adolescents, January 29, 2008). In the present study the subjects are stopping ART for a relatively short period of up to 16 weeks, during which they will be closely monitored.

The subjects will remain on ART throughout the immunization period and for another 10 weeks after the final immunization, in order to prevent interference from the immune response caused by viral rebound associated with ART interruption. In all studies with Vacc-4x, subjects have been vaccinated when on ART with no loss of viral control. Viral rebound was only observed after subjects stopped ART. During the ART interruption period, subjects are being monitored carefully and ART will be restarted if subjects exceed pre-specified criteria for viral load and CD4 reduction or for any other medical reasons (4.4.1.3 Criteria for Restart of ART).

# 2 STUDY OBJECTIVES AND ENDPOINTS

## 2.1 Study Objectives

### 2.1.1 Primary Objective

The primary objective of this study is to evaluate if a re-boost with Vacc-4x could

a) reduce the viral load set-point, and

b) increase the immune response obtained following immunization with Vacc-4x in Study CT-BI Vacc-4x 2007/1 (EudraCT Number 2007-006302-13).

### 2.1.2 Secondary Objectives

- To evaluate the effect of a re-boost with Vacc-4x on CD4 counts and CD8 counts.

- To assesses *in vivo* immunogenicity of Vacc-4x by evaluation of DTH and to compare the DTH response to the DTH response observed in the initial study; CT-BI Vacc-4x 2007/1 (EudraCT Number 2007-006302-13).
- To evaluate the safety and tolerability of re-boosting with Vacc-4x.

## 2.2 Study Endpoints

### 2.2.1 Efficacy Endpoints

#### 2.2.1.1 Primary Efficacy Endpoints

- Viral load set point (mean of VL count Visit 8 and Visit 9) for subjects discontinuing ART according to protocol and *not* resuming ART until Visit 9 compared to:

  - viral load set point* for subjects not resuming ART before week 52 or later in the CT-BI Vacc-4x 2007/1 study and who entered this study,

  or if week 52 not reached in the previous study

  - viral load set point** for subjects resuming ART at the earliest week 40 in the CT-BI Vacc-4x 2007/1 study and who entered this study.

  ** The VL set point was defined as the mean of VL count week 48 and week 52 week in subjects who did not resume ART.*

  ***The VL set point was defined as the week 40 VL count for subject resuming ART week 40 or the mean of the last two VL counts prior to ART resumption for subjects resuming ART week 44 or later but before week 52.*

  Comparison will also be made to pre-ART viral load values, if available from the CT-BI Vacc-4x 2007/1 study.

- T-cell response will be measured by ELISPOT as well as by T-cell proliferative response assay (Flow Cytometry) and intracellular cytokine staining at Visit 2 (before re-boosting), Visit 4 (two weeks after re-boosting), Visit 6, Visit 9 and Visit 10. These will be compared to the responses reported in the study CT-BI Vacc-4x 2007/1.

#### 2.2.1.2 Secondary Efficacy Endpoints

- CD4 counts at all visits in the study, absolute numbers of CD4 counts, and treatment emergent changes of CD4 counts from mean of Screening and Visit 2.
- Change (and percent change) in CD4 count from Visit 5 (discontinue ART) to Visits 6, 7, 8, 9 and 10 (End of Study).
- CD8 counts at all visits in the study, absolute numbers of CD8 counts, and treatment emergent changes of CD8 from mean of Screening and Visit 2.
- Change (and percent change) in CD8 count from Visit 5 (discontinue ART) to Visits 6, 7, 8, 9 and 10 (End of Study).

For each individual subject both the absolute and relative changes in CD4 and CD8 values registered at Visit 5, and at Visit 9 (or the last value before reinitiated ART, if ART reinitiated before Visit 9) will be compared with the corresponding values registered at Week 28 (ART discontinued) and Week 52 (or last visit before ART re-initiation) in the initial study CT-BI Vacc-4x 2007/1 (EudraCT Number 2007-006302-13).

- DTH (both induration and erythema) at Visit 2 and Visit 4, in terms of observed areas ($mm^2$ length x height) and also in terms of a positive test, defined as an area ≥ $10mm^2$ DTH-tests, both areas and number of positive tests registered at Visit 2 will be compared with the corresponding values registered at Visit 4.

In addition the values for these DTH parameters at Visit 4 will be compared with the DTH response reported at Week 18 in the initial study CT-BI Vacc-4x 2007/1 (EudraCT Number 2007-006302-13).

### 2.2.2 Safety Endpoints

- Proportion of subjects who experience similar virologic suppression 8 weeks after re-initiation of ART (Visit 10) as before ART interruption (Visit 5 value).
- AEs, recorded continuously from screening to Visit 10 (End of Study).
- Vital signs (heart rate, blood pressure) at Screening, and selected visits through Visit 10 (End of Study).
- Clinical laboratory evaluations (clinical chemistry, hematology) at screening, and selected visits through Visit 10 (End of Study); absolute values and treatment emergent changes from mean Screening and Week 1.

# 3 STUDY DESIGN

## 3.1 Overall Study Design and Flow Chart

The study is an open label, multicenter, follow-up, re-boosting study of subjects who previously completed the immunization regimen with Vacc-4x active and stopped ART (at Week 28) in the CT-BI Vacc-4x 2007/1 Study (EudraCT Number 2007-006302-13). No restart of ART is required. The subjects will be re-boosted with two immunizations (Visit 2 and Visit 3) of Vacc-4x, 1.2 mg peptides (12 mg/mL), with a 2 week interval between immunizations.

At Visit 5 ART medication (if being used) will be stopped in subjects whose CD4 count ≥ $350x10^6$/L, for a 16 week period. At Week 29 ART will be restarted, according to the Investigator and subject’s decision, and the subjects will be followed for an additional 8 weeks (End of Study; Visit 10).

DTH will be measured at Visit 2, and at Visit 4 (3 weeks after the second re-boosting immunization).

Viral load, CD4 and CD8 counts will be measured at all study visits. T-cell responses will be measured at Visits 2, 4, 6, 9 and 10 (End of Study) both by ELISPOT, T-cell proliferation assay and intracellular cytokine staining.

Vital signs and clinical laboratory tests will be done at all study visits.

Monitoring of AEs and concomitant medications will be done continuously from the time of signing ICF through the End of Study.

### Table 1. Time and Events Schedule

| Parameters | Screening/ Verification Inclusion | Re-vaccination cycle | | | | | | | | Follow-up |
|---|---|---|---|---|---|---|---|---|---|---|
| | | Re-boost period | | | Follow-up on ART | ART-free follow-up period | | | | END of STUDY |
| **Visit:** | **1** | **2** | **3** | **4** | **5** | **6** | **7** | **8** | **9** | **10** |
| Day | | 1 | 15±2 | 28±2 | 84±3 | 112±7 | 140±7 | 168±7 | 196±7 | 252±7 |
| **Week** | **-4** | **0** | **2** | **4** | **12** | **16** | **20** | **24** | **28** | **36** |
| Re-boost: Adjuvant + Vacc-4x | | x | x | | | | | | | |
| DTH[a] | | x | | x | | | | | | |
| ART status | On or Off | | | | Stop[g] | | | | Re-start[h] | |
| Informed Consent | x | | | | | | | | | |
| Inclusion/exclusion | x | | | | | | | | | |
| Physical examination incl. Weight and Height[b] | x | x | | | x | | | | x | x |
| Medical History | x | | | | | | | | | |
| Vital Signs | x | x | x | x | x | x | x | x | x | x |
| Clinical Chemistry/Hematology[c] | x | x[i] | x[i] | x | x | x | x | x | x | x |
| Viral load/CD4 and CD8 Count[d] | x | x[i] | x[i] | x | x | x | x | x | x | x |
| T-cell Response[e] /Proviral DNA | | x[i] | | x | | x | | | x | x |
| Pregnancy Test[f] | | x | x | | | | | | | |
| Adverse Events | x | x | x | x | x | x | x | x | x | x |
| Concomitant Medications | x | x | x | x | x | x | x | x | x | x |

a. Immunization for the DTH skin reaction is performed on day indicated; the test is evaluated 48 hours after immunization. This evaluation can be made by the subject, if properly trained.
b. Height only once at Screening.
c. Includes measurements of hemoglobin, CRP, WBC, platelets, creatinine, AST (SGOT), ALT (SGPT), LDH, ALP, $\gamma$-GT, albumin, total bilirubin, and $\beta$2-microglobulin.
d. Viral load will be quantified by HIV RNA copies/mL using PCR. CD4 and CD8 counts will be determined using flow cytometry.
e. T-cell response will be measured by ELISPOT as well as T-cell proliferative response assay and intracellular cytokine staining.
f. A urine pregnancy test will be administered and a negative test will be confirmed, prior to study drug administration.
g. If on ART subjects whose CD4 count is $\geq 350x10^6$/L and virally controlled at Visit 5 are expected to stop ART.
h. Restart according to Investigator's/subject's decision.
i. Blood samples should be taken before immunization (re-boosting).

## 3.2 Rationale for Study Design and Treatment Regimens

This study includes two re-boosting immunizations with Vacc-4x to subjects who have previously been immunized with the same peptides (Vacc-4x) followed by interruption of ART in the study CT-BI Vacc-4x 2007/1 (EudraCT Number 2007-006302-13). This study is performed to evaluate whether or not a re-boosting with Vacc-4x could enhance the immunological effect and further reduce the viral load set-point achieved after the first immunization regimen. This information is highly desired in planning future vaccination regimens.

# 4 SELECTION AND WITHDRAWAL OF SUBJECTS

## 4.1 Inclusion

All subjects to be included will have participated in the CT-BI Vacc-4x 2007/1 study (EudraCT Number 2007-006302-13), completed the immunization regimen with active Vacc-4x and stopped ART at Week 28.

## 4.2 Inclusion Criteria

1. Completed immunization regimen with Vacc-4x active and stopped ART (at Week 28) in the CT-BI Vacc-4x 2007/1 study. (No re-start of ART is required).
2. Documented pre-study CD4 cell count $\geq 400x10^6$/L.
3. Documented pre-study viral load < 300 000copies/mL.
4. Signed informed consent.

## 4.3 Exclusion Criteria

A subject will not be eligible for this study if any of the following criteria apply:

1. Reported AIDS-defining illness within the previous year.
2. Malignant disease.
3. On chronic treatment with immune-suppressive therapy.
4. Unacceptable values of the hematologic and clinical chemistry parameters, as judged by the Investigator, including creatinine values >1.5 x upper limit of normal (ULN), and AST, ALT and alkaline phosphatase (ALP) values >2.5 x ULN.
5. Concurrent chronic active infection such as viral hepatitis B or C or tuberculosis.
6. Pregnant or breastfeeding women.
7. Women of childbearing potential not using reliable and adequate contraceptive methods (defined as: use of oral, implanted, injectable, mechanical or barrier products for the prevention of pregnancy; practicing abstinence; sterile) during the 28±2 days re-boosting period including the DTH and for 2 weeks after the DTH test, or sexually active male subjects with partners of child bearing potential unwilling to practice effective contraception during the 28±2 days re-boosting period including the DTH and for 12 weeks after the DTH-test.
8. Current participation in other clinical therapeutic studies.
9. Incapability of compliance to treatment protocol, in the opinion of the Investigator.

## 4.4 Withdrawal of Subjects

A subject may voluntarily withdraw or be withdrawn from the study at any time for reasons including, but not limited to, the following:

- The subject wishes to withdraw from further participation.
- Continuation in the study would be impractical or detrimental to the subject's safety in the opinion of the Investigator.
- The subject becomes pregnant or is breast feeding.
- The subject is significantly noncompliant with the protocol or requires a concomitant medication prohibited by the protocol.

If a subject is discontinued prematurely from the study, the Investigator must provide an explanation in writing and complete the appropriate End of Study Assessment. In the event a subject discontinues prematurely due to an AE, the AE will be followed until it resolves (returns to normal or value before study start) or stabilizes, or until it is judged by the Investigator to be no longer clinically significant.

### 4.4.1 Vaccine Toxicity Management

#### 4.4.1.1 Injection Site Reactions

The following local reactions will be recorded as AEs:

- Swelling or redness at the injection site: The size (in cm) of the reaction at its largest from edge to edge will be estimated as 'None, (<0.5 cm)', '1 cm (0.5 - 1.4 cm)', '2 cm (1.5 - 2.4)', '3 cm (2.5 - 3.4 cm)', or 'over 3 cm (≥3.5 cm)'.
- Pain, tenderness, or other reactions at the injection site. The severity of pain or other reactions will be categorized as: 'None,' 'Mild' (awareness of symptom but easily tolerated), 'Moderate' (discomfort enough to cause interference with usual activities), or 'Severe' (incapacitating with inability to work or do usual activity).

For injection site reactions judged to be life-threatening or severe, the Sponsor (or designee) should be notified within 24 hours, and further vaccines should not be given to the subject prior to consultation with the Medical Monitor and Sponsor.

#### 4.4.1.2 Systemic Reactions

Systemic reactions will be graded according to the Division of AIDS Table for Grading Severity of Adult and Pediatric Adverse Events, Version 1.0 December 2004; Clarification August 2009 (see Appendix 15.1) and recorded as AEs. The Sponsor (or designee) should be contacted within 24 hours for any systemic Grade 3 or 4 reactions thought definitely, possibly, or probably related to treatment. Further vaccines should not be given to the subject prior to consultation with the Medical Monitor and Sponsor.

#### 4.4.1.3 Criteria for Restart of ART

During the period of interrupted ART, ART should be resumed if:

- CD4 cell count falls below $350x10^6$/L (test must be repeated and confirmed within two weeks).
- CD4 counts fall by ≥50% (test must be repeated and confirmed within two weeks).
- Viral load is >300000 copies/mL on two consecutive tests (the re-test should be within two weeks of the last test).
- Development of HIV- or AIDS-related events, including any of the clinical events listed in Appendix 15.2 (AIDS-Defining Illnesses).
- If in the opinion of the site Investigator the subject, as a result of stopping ART, is likely to be (or has a reasonable possibility of) suffering from clinical or laboratory features that could be controlled by re-starting ART, strong consideration should be given to restarting the subject's antiretroviral agents. Examples of these features include antiretroviral syndrome, significant clinical thrombocytopenia without other probable cause, and onset of relevant cardiovascular or renal clinical or laboratory features.

#### 4.4.1.4 Study Discontinuation/Stopping Rules

The study may be discontinued at any time by the Sponsor, Independent Review Board (IRB) Independent Ethics Committee (IEC), the FDA or other regulatory authorities as part of their duties to ensure that research subjects are protected.

# 5 TREATMENT OF SUBJECTS

## 5.1 Study Drug and Dosages

### 5.1.1 Study Drug Description

#### 5.1.1.1 Vacc-4x

The Investigational Medicinal Product (IMP), Vacc-4x, consists of four synthetic peptides (Vacc-10 acetate, Vacc-11 acetate, Vacc-12 acetate, and Vacc-13 acetate), each corresponding to conserved domains on the HIV-1 p24 capsid protein representing the native Gag regions with residues 166-185, 252-269, 264-284, and 335-354, respectively.

Vacc-4x is manufactured in accordance with Good Manufacturing Practice (GMP). Vacc-4x is supplied as sterile vials of freeze-dried white powder. There is no additional ingredient in the product.

Vacc-4x should be administered intradermally superficial to the deltoid muscle at a dose of 0.1 mL of a 12 mg/mL solution 10 to 20 minutes after administration of Leukine®.

#### 5.1.1.2 Adjuvant: Leukine®

GM-CSF is a growth and differentiation factor for epidermal Langerhans cells. Intradermal GM-CSF has demonstrated adjuvant properties in the induction of immune responses (Hodge, 2006; Arellano, 2004).

Leukine® is a commercially available product and is supplied as sterile vials of sargramostim, rhu-GM-CSF, 250 µg/vial.

Leukine® should be administered intradermally superficial to the deltoid muscle at a dose of 0.1 mL of a 0.60 mg/mL solution.

### 5.1.2 Dosage and Administration of Study Drug

Vacc-4x is administered as an intradermal injection in combination with Leukine®,. 10 to 20 minutes before each administration of Vacc-4x, 60 µg (0.1 mL, 0.6 mg/mL) Leukine® is administered intradermally as an adjuvant (Gjertsen, 2001; Brunsvig, 2006; Bernhardt, 2006). Vacc-4x may only be administered intradermally at the same site as Leukine®, superficial to the deltoid muscle.

When administering the intradermal injection, utmost care must be taken so that no material is injected subcutaneously. If administered correctly, after puncture of the skin a small bleb should appear following the injection of only a small amount of product. An injection that is too superficial should be avoided as this will result in loss of the sample volume from the injection site during injection or after withdrawal of the needle.

At Visit 2 and Visit 3 subjects will receive Leukine®, as adjuvant, administered intradermally superficial to the deltoid muscle at a dose of 0.1 mL of 0.60 mg/mL solution. 10 to 20 minutes later Vacc-4x will be administered intradermally at a dose of 0.1 mL of a 12 mg/mL solution, and at the same site as Leukine®.

### 5.1.3 DTH Administration

At Visit 2 and Visit 4 a DTH testing will be performed. This is an injection of 0.1 mL of a 4.0 mg/mL Vacc-4x solution to be administered intradermally at the front of arm in the absence of Leukine®.

### 5.1.4 Blinding

There will be no blinding as this study is an open study.

### 5.1.5 Rationale for Dosing Regimen

The re-boosting dose and immunization procedure are as in the previous study: CT-BI Vacc-4x 2007/1 study (EudraCT Number 2007-006302-13).

### 5.1.6 Subject Compliance

The study treatment (Vacc-4x and Leukine®) will be administered by trained study personnel, and will not be distributed to subjects, thus subject compliance is assured.

Clinical staff at the site is to record the timing and sites of administrations in the source notes, which will be transcribed onto the case report forms (CRFs). Any variations to the treatment schedule should be documented.

Ongoing compliance with ART during the re-boosting period will be supported by the site clinicians according to standard site protocols and will not be formally evaluated in this study. Study participants are not permitted to be enrolled in any other clinical trial while participating in this study.

### 5.1.7 Overdose and Toxicity Management

Following each immunization, the subject will be required to remain at the clinic for at least one hour for observation of any immediate reaction to the immunization.

As both study treatments will only be administered by study personnel no overdoses are expected. There is no experience with human overdose of Vacc-4x. The experience in the nonclinical intravenous studies with mice and rats suggests that even if Vacc-4x is inadvertently delivered to the circulatory system clinically important toxicity should not be expected.

<u>Emergency Procedures</u>: In previous human studies, two subjects experienced transient vasovagal reactions after exposure to Vacc-4x (as an immunization or DTH test). The subjects recovered quickly. In case of anaphylactic reaction, the site should use standard supportive treatment and emergency equipment must be available at the site. Cases of anaphylaxis should be reported as SAEs. Study drug should be permanently discontinued.

## 5.2 Concurrent Medications and Non-drug Therapies

Administration of all concomitant medications must be reported in the appropriate section of the CRF along with dosage information, dates of administration, and reasons for use. Trade names (product names) for concomitant medication should be used, if possible. The total daily dose should be recorded whenever possible. Additionally, diagnostic, therapeutic, or surgical procedures performed during the study period must be recorded in the comments section of the corresponding AE report.

### 5.2.1 Permitted Medications

Any treatment which is considered necessary for the subject's welfare (including supportive care), and which will not interfere with the study drug, may be given at the discretion of the Investigator.

### 5.2.2 Prohibited Medications

Subjects are prohibited from concomitant use of any of the following medications:

- Any immune-modulating therapy (except Meningococcal vaccination).

- Chronic corticosteroids, except for asthma inhalers or topical use.
- Other investigational drugs, agents, or devices.

## 5.3 Study Drug Management

### 5.3.1 Packaging of Study Drug

Vacc-4x will be provided as an IMP with the appropriate cautionary statements included on the label per local regulatory requirements. Leukine® will be provided as a marketed product.

### 5.3.2 Labeling of Study Drug

The vials of Vacc-4x will be labeled with the protocol number (Europe only), administration instructions, expiration date (Europe only), lot number, and Sponsor address. Additional information will be included according to local laws and regulations. Leukine® will be additionally labeled for clinical trial use as required by local regulatory requirements.

### 5.3.3 Preparation of Study Drug

The preparation of Vacc-4x should be performed by the study site pharmacist (or other responsible person). Vacc-4x is to be reconstituted with 0.30 mL of sterile Water for Injection (WFI) without preservative, United States Pharmacopoeia (USP) or European Pharmacopoeia (Ph. Eur.). Dilution of the lyophilized powder with 0.30 mL results in a total peptide concentration of 12 mg/mL per vial. A volume of 0.1 mL of the re-suspended solution is administered to the subject resulting in a net peptide content per dose of 1.2 mg. All handling of the product should be in compliance with handling of sterile product for injection. The date (DD/MM/YYYY) and time (XX: XX [24 hours]) of reconstitution should be recorded. The reconstituted product should be used as soon as possible after preparation (and no later than 8 hours after preparation).

The preparation of the Leukine® should be performed by the study site pharmacist (or other responsible person). Leukine® is to be reconstituted with sterile WFI (without preservative), USP or Ph. Eur. (0.4 mL) to yield the final concentration of 0.6 mg/mL. The solution should be clear and colorless. A volume of 0.1 mL (0.06 mg) will be administered intradermally superficial to the left deltoid muscle. All handling of the product should be in compliance with handling of sterile product for injection. The date (DD/MM/YYYY) and time (XX: XX [24 hours]) of reconstitution should be recorded. The reconstituted product should be used as soon as possible after preparation (and no later than 6 hours after preparation).

### 5.3.4 Storage of Study Drug

Lyophilized Vacc-4x should be stored at 2 to 8°C (36 to 46°F) and within the expiry date printed on the certificate of analysis. Reconstituted Vacc-4x should be stored at 2 to 8°C (36 to 46°F). Once reconstituted, Vacc-4x is stable for 8 hours at 2 to 8°C (36 to 46°F).

Lyophilized Leukine® should be stored at 2 to 8°C (36 to 46°F) but should not be used beyond the expiration date printed on the vial. Reconstituted Leukine® will remain stable at 2 to 8°C (36 to 46°F) for 6 hours, according to manufacturer's guidelines.

### 5.3.5 Study Drug Shipping and Handling

Vacc-4x and Leukine® will be dispatched to the site from Penn Pharmaceutical Services Ltd.

Study drug must be prepared, dispensed and administered according to procedures described herein and in the Investigational Product Handling Instructions for the study. Only subjects enrolled in the study may receive study drug, in accordance with all applicable regulatory requirements.

### 5.3.6 Study Drug Accountability

The pharmacy is responsible for maintaining accurate records of Vacc-4x and Leukine® shipments (receipts) and for recording the use of Vacc-4x and Leukine® on accountability forms. The pharmacy is also responsible for the dispensation and return of Vacc-4x and Leukine®. Any Vacc-4x and Leukine® accidentally or deliberately destroyed must be accounted for.

Partially used vials must be accounted for before they are destroyed at the study center according to local procedures or returned to the Sponsor.

Unused vials must be destroyed locally or returned to the Sponsor. At the finalization of the clinical study, the overall numbers of Vacc-4x and Leukine® vials shipped to the study center and the number destroyed and/or returned must be provided by the pharmacy. An account must be given of any discrepancies that might have occurred during the study.

Empty tubes of sterile WFI and vials containing residual Vacc-4x or Leukine® should be retained until final drug accountability has been performed and until the Sponsor provides the instruction to discard the samples.

# 6 STUDY PROCEDURES

## 6.1 Time and Events Schedule

The study procedures to be conducted for each subject are divided into the following study phases:

- Screening (up to 4 weeks)
- Re-boosting (up to 28±2 days including DTH test) followed by 8 weeks before cessation of ART (Visit 5).
- ART-free follow-up (16 weeks)
- Follow-up after restart of ART (8 weeks)

The procedures to be completed during each of these phases are presented in the Time end Events Schedule (Section 3.1, Table 1). Detailed descriptions of the assessments and the definition of study endpoints are provided in Section 7 (Study Assessments and Measurements). Any deviation from study procedures should be noted in the source documents and significant deviations should be reported to the Sponsor immediately.

## 6.2 Subject Informed Consent

Prior to performing any study procedures, the Investigator (or his/her designated staff) will obtain written informed consent from the subject as described in Section 11.6.1. After reading the informed consent document and having all questions answered, the subject must give consent in writing. The subject's consent must be confirmed at the time of the subject’s signature by the personally dated signature of the person conducting the informed consent discussions. A copy of the signed consent document must be given to the subject. The Investigator will retain the original signed consent document. The Investigator will not undertake any measures specifically required for the clinical study until valid consent has been obtained.

## 6.3 Procedures by Study Phase

### 6.3.1 Screening

#### 6.3.1.1 Screening Visit (Visit 1)

Having given consent, subjects will undergo Screening assessments to determine whether they are eligible to participate in the study according to the criteria listed in Section 4.2 and Section 4.3. The following evaluations will be performed during Screening:

- Review of inclusion/exclusion criteria.
- ART status (on ART or not on ART), including drug name(s) and date(s) started.
- Medical history since end of study CT-BI Vacc-4x 2007/1 (EudraCT Number 2007-006302-13)
- Physical examination (including height).

- Vital signs (blood pressure, heart rate).
- Clinical chemistry and hematology (hemoglobin, C-reactive protein [CRP], white blood cells (WBCs), platelets, creatinine, AST (SGOT), ALT (SGPT), lactate dehydrogenase (LDH), ALP, gamma Glutamyl Transferase (γ-GT), albumin, total bilirubin, β2-microglobulin).
- If restarted ART after Week 52 in CT-BI Vacc-4x 2007/1 study (EudraCT Number 2007-006302-13), last viral load before restart ART should be recorded.
- Viral load, CD4 and CD8 counts.
- Concomitant medications and AEs.

At the Investigator's discretion, laboratory tests may be ordered through a local laboratory to confirm a subject's current status regarding chronic active infections or AIDS-related illnesses in order to confirm eligibility in the trial if the status is not sufficiently documented in the subject's medical records.

### 6.3.2 Re-boosting, DTH testing and Follow-up on ART

Re-boosting consists of two administrations of Vacc-4x + Leukine® with a 2 weeks (±2 days) interval between immunizations.

#### 6.3.2.1 Visit 2

This visit will occur at least five days but not more than 28 days after the Screening Visit. The Visit 2 should occur after the results of the Screening evaluations are available to confirm eligibility.

The following procedures will be performed in the following order (it is important that all blood samples are taken before administration of study treatments):

- Review of concomitant medications and AEs.
- Physical examination including weight.
- Vital signs (blood pressure, heart rate).
- Urine pregnancy test (all females of child bearing potential).
- Clinical chemistry and hematology.
- Viral load, proviral DNA, CD4 and CD8 counts.
- T-cell response (ELISPOT, T-cell proliferative response assay and intracellular cytokine staining).
- Intradermal administration of Leukine®.
- Intradermal administration of Vacc-4x, 10 to 20 minutes after administration of Leukine®.
- Administration of DTH test (test to be evaluated 48 hours after administration).

The subject should remain in the clinic for <u>1 hour</u> after the administration of study drug and the DTH test to be monitored for adverse reactions to the immunization.

#### 6.3.2.2 Visit 3

This visit should be 15 (±2) days from Visit 2.

The following procedures will be performed in the following order (it is important that all blood samples are taken before administration of study treatments):

- Review of concomitant medications and AEs.
- Vital signs (blood pressure, heart rate).
- Urine pregnancy test (all females of child bearing potential).
- Clinical chemistry and hematology.
- Viral load, CD4 and CD8 counts.
- Intradermal administration of Leukine®.
- Intradermal administration of Vacc-4x, 10 to 20 minutes after administration of Leukine®.

The subject should remain in the clinic for <u>1 hour</u> after the administration of study drug to be monitored for adverse reactions to the immunization.

#### 6.3.2.3 Visit 4

This visit should be 28 (±2) days from Visit 2.

The following procedures will be performed:

- Review of concomitant medications and AEs.
- Vital signs (blood pressure, heart rate).
- Clinical chemistry and hematology.
- Viral load, proviral DNA, CD4 and CD8 counts.
- T-cell response (ELISPOT, T-cell proliferative response assay and intracellular cytokine staining).
- DTH test (test to be evaluated 48 hours after immunization).

The subject should remain in the clinic for <u>1 hour</u> after the administration of the DTH test to be monitored for adverse reactions to the test.

#### 6.3.2.4 Visit 5

This visit should be 84 (±3) days from Visit 2.

If on ART, the subject will be instructed to now stop ART if their CD4 count is $\geq$ 350 x $10^6$/L and virally controlled (undetectable VL [<50copies] over a period of three months on a stable antiretroviral regimen with no more than one blip allowed). If the CD4 count is <350 x $10^6$/L and/or the virus is not controlled, the subject will be instructed to remain on ART. The instruction will be based on the results of CD4 count and viral load from this visit. As soon as possible when these

results are available, and at the latest within one week after this visit, the investigator will contact the subject by phone and tell the subject to stop ART, or not to stop ART. Stopping of ART will be done according to local guidelines and their current clinical judgment and based on the individual ART the subjects are receiving. Subjects will be instructed to write down the date(s) when stopping ART and take this information to the investigator at the next visit.

The Investigator needs to see the results from the blood sampling at Visit 5 before the subject can stop ART. The information to the subject that he/she can stop ART and instructions on how to do this can be made by telephone. It is important that the treatment interruption period has a full 16 week duration.

The following procedures will be performed:

- Review of concomitant medications and AEs.
- Physical examination including weight.
- Vital signs (blood pressure, heart rate).
- Clinical chemistry and hematology.
- Viral load, CD4 and CD8 counts.

### 6.3.3 ART-free Follow-up Period

#### 6.3.3.1 Visit 6

This visit should be 112 (±7) days from Visit 2.

The following procedures will be performed:

- Recording date(s) of stopping ART
- Review of concomitant medications and AEs.
- Vital signs (blood pressure, heart rate).
- Clinical chemistry and hematology.
- Viral load, proviral DNA, CD4 and CD8 counts.
- T-cell response (ELISPOT, T-cell proliferative response assay and intracellular cytokine staining).

#### 6.3.3.2 Visit 7

This visit should be 140 (±7) days from Visit 2.

The following procedures will be performed:

- Review of concomitant medications and AEs.
- Vital signs (blood pressure, heart rate).
- Clinical chemistry and hematology.

- Viral load, CD4 and CD8 counts.

#### 6.3.3.3 Visit 8

This visit should be 168 (±7) days from Visit 2.

The following procedures will be performed:

- Review of concomitant medications and AEs.
- Vital signs (blood pressure, heart rate).
- Clinical chemistry and hematology.
- Viral load, CD4 and CD8 counts.

#### 6.3.3.4 Visit 9

This visit should be 196 (±7) days from Visit 2.

At this visit it will be recommended that the subjects restart ART.

The following procedures will be performed:

- Review of concomitant medications and AEs.
- Physical examination including weight.
- Vital signs (blood pressure, heart rate).
- Clinical chemistry and hematology.
- Viral load, proviral DNA, CD4 and CD8 counts.
- T-cell response (ELISPOT, T-cell proliferative response assay and intracellular cytokine staining).

### 6.3.4 Follow-up Period to End of Study

The follow-up period after restarting ART at Visit 9 consists of an 8-week period.

#### 6.3.4.1 Visit 10 (End of Study)

This visit should be 252 (±7) days from Visit 2.

The following procedures will be performed:

- Review of concomitant medications and AEs.
- Physical examination including weight.
- Vital signs (blood pressure, heart rate).
- Clinical chemistry and hematology.
- Viral load, proviral DNA, CD4 and CD8 counts.

- T-cell response (ELISPOT, T-cell proliferative response assay and intracellular cytokine staining).

## 6.4 Subject Discontinuation (Lost to Follow-up)

In the event that a subject elects not to return to the clinic for the final study visit (End of Study), the Investigator must make every effort to contact the subject to review all AEs. In the event that a subject drops out of the study at any time, the reason for discontinuation must be fully documented in the source documents and the CRF. The site personnel will document the AEs and any other assessments in the source documents and will make every effort to complete all required End of Study assessments.

# 7 STUDY ASSESSMENTS AND MEASUREMENTS

## 7.1 Demographic and Screening Assessments

The following information will be collected at the Screening Visit:

- Date of birth and the subject identification number from the Study CT-BI Vacc-4x 2007/1 (EudraCT Number 2007-006302-13). to verify the subject's participation in that study
- Medical history (from end of Study CT-BI Vacc-4x 2007/1) HIV-1 disease status as measured by viral load and CD4 and CD8 counts.
- ART medications and other concomitant medications.
- Clinical chemistry and hematology.

## 7.2 Efficacy Assessments

### 7.2.1 Delayed-type Hypersensitivity – Test Administration

DTH skin reactivity testing represents a simple *in vivo* method for monitoring cellular immune responses. The DTH test consists of Vacc-4x with a concentration of 4.0 mg/mL. At the pharmacy, a DTH dose is prepared to the final concentration of 4.0 mg/mL as described in the Pharmacy Manual. A volume of 0.1 mL should be drawn into a syringe ready for use. All handling of the product should be in compliance with handling of sterile product for injection. The reconstituted product should be used as soon as possible after preparation, and within a maximum of 8 hours when stored at 2 to 8$^{o}$C.

DTH testing will be performed at the time of the first re-boost (Visit 2) and 2 weeks after last re-boost (Visit 4). An injection of 0.1 mL of a 4.0 mg/mL Vacc-4x should be administered intradermally at the front of arm; the DTH test does not require prior injection of adjuvant.

The DTH test results will be evaluated by study personnel or by the subject (as done in the previous study) 48 hours after the tests are performed. For each DTH test, evaluation of the injection site will include documentation of palpable skin induration and skin erythema. A positive DTH test is defined

as an area of induration or erythema $\geq 10mm^2$ (length x height). Study personnel will record DTH results in the subject's source documents and transcribe them into the CRF.

### 7.2.2 CD4 and CD8 Counts

A blood sample (approximately 4 mL) will be collected in tubes containing ethylenediamine tetraacetic acid (EDTA) at all visits in the study. CD4 and CD8 cells will be measured by flow cytometry. Tests will be performed at central laboratories in the US and in Europe. Handling of samples at the site will be detailed in laboratory manuals.

### 7.2.3 HIV Viral Load

A blood sample (approximately 6 mL) will be collected in tubes containing EDTA at all visits in the study. Viral load will be quantified by HIV RNA copies/mL using polymerase chain reaction (PCR). Tests will be performed at central laboratories in the US and in Europe. Handling of samples at the site will be detailed in laboratory manuals.

### 7.2.4 Proviral HIV DNA

To provide additional exploratory information on the HIV viral load, proviral HIV DNA will be analyzed at the central laboratory in Europe (Lausanne, Switzerland) on the same PBL samples mentioned in Section 7.2.5. Proviral DNA will be analyzed at Visit 2, 4, 6, 9 and 10 (End of Study). Handling of samples at the site will be described in detail in laboratory manuals.

### 7.2.5 T-cell Response

Blood samples (approximately 60 mL) will be drawn and the peripheral blood lymphocytes (PBL) separated at each clinical trial site. The PBL will be frozen at < -70°C and shipped frozen within 21 days of collection to the central laboratory in Europe (Lausanne, Switzerland) for evaluation. Handling of samples at the site will be detailed in laboratory manuals. The T-cell response to the p24 antigen (including Vacc-4x peptides) will be measured using an ELISPOT test, T-cell proliferation and intracellular cytokine staining at Visit 2, 4, 6, 9 and 10 (End of Study).

ELISPOT is a sensitive immunoassay that will enable detection of antigen-specific T-cells generated after Vacc-4x re-boosting compared with Visit 2 (before re-boosting).

Blood samples will also be analyzed by flow cytometry. Collected cells will be labeled with fluorescent dye (carboxyfluorescein succinimidyl ester [CFSE]), and upon antigen-challenge, activity (intracellular cytokines/ biomarkers, proliferation, T-cell receptor repertoire, surface activation markers) of antigen-specific cells will be visualized and quantified.

T-cell proliferative responses will be evaluated using the T-cell proliferation assay modeled after a previously reported assay (Kvale, 2005). T-cell proliferative responses will be compared with data reported in the initial study CT-BI Vacc-4x 2007/1 (EudraCT Number 2007-006302-13).

Results for intracellular cytokine testing will be presented in a single written report appended to the Clinical Study Report, to be prepared by the central laboratories. These outputs will be supplied directly to the Sponsor.

In addition, supernatant from the T-cell proliferation assay will be collected for further possible biomarker (e.g., cytokines) testing by Bionor Immuno AS in order to depict Vacc-4x induced T-cell responses. A single written report appended to the Clinical Study Report will be performed by Bionor Immuno AS.

## 7.3 Safety Assessments

### 7.3.1 Medical History

The medical history from the time the subject finished the previous study (CT-BI Vacc-4x 2007/1; EudraCT Number 2007-006302-13) up to inclusion in this re-boosting study will be collected.

### 7.3.2 Physical Examination

Physical examinations will be performed at Visit 1, 2, 5, 9 and 10 (End of Study). Physical examination will include height (only at Screening), weight and evaluation of the subject's medical condition. Any new or worsening of medical condition compared to the Visit 2 (first re-boosting dose) should be recorded as an AE on the CRF.

### 7.3.3 Vital Signs

At all visits in the study, vital signs (heart rate, systolic and diastolic blood pressure) will be measured prior to administration of study drug (if applicable) with the subject in the sitting position. Vital signs will be measured after the subject has been sitting at rest for 5 minutes.

### 7.3.4 Clinical Laboratory Tests

Blood samples will be collected (approximately 10 mL) at all visits in the study. It is important that blood samples are taken prior to administration of study treatments. For the European sites the blood samples will be sent to Covance laboratory in Geneva, Switzerland, for analysis. For the US sites the blood samples will be sent to Covance laboratory in Indianapolis, US, for analysis.

The following clinical chemistry and hematology parameters will be measured:

| **Chemistry** | **Hematology** |
|---|---|
| AST | Hemoglobin |
| ALT | WBC count with differential |
| Creatinine | Platelets |
| CRP | |
| ALP | |
| Albumin | |
| Total Bilirubin | |
| B2-microglobulin | |
| Γ-Glutamyl Transferase | |
| LDH | |

### 7.3.5 Pregnancy

If relevant, a urine pregnancy test will be administered at Visit 2 and Visit 3. The results must be reviewed prior to initiating study treatment at each visit.

# 8 ADVERSE EVENT MANAGEMENT

## 8.1 Non-serious and Serious Adverse Events

The Investigator is responsible for the detection and documentation of events meeting the criteria and definition of an AE or SAE as provided in this protocol (see below). All AEs and SAEs that occur from the time written informed consent is obtained until completion of the study at Visit 10 must be documented. Information to be collected includes the description, date and time of onset and stop, intensity, duration, causality, seriousness relationship, outcome and action taken (Section 8.1.4). Even if the AE is assessed by the Investigator as not reasonably attributable to study drug, its occurrence must be recorded in the source documents and reported on the CRF.

During each study visit, after the subject has had an opportunity to spontaneously mention any problems, the Investigator should inquire about the occurrence of AEs. The following are examples of open-ended questions that may be used to obtain this information:

- “How are you feeling?”
- “Have you had any medical problems since your last visit/assessment?”
- “Have you taken any new medicines, other than those given to you in this study, since your last visit/assessment?”

### 8.1.1 Definition of an Adverse Event

An AE is any untoward medical occurrence in a subject administered a pharmaceutical product that does not necessarily have a causal relationship with this treatment.

An AE can therefore be any unfavorable and unintended sign (including an abnormal laboratory finding, for example), symptom, or disease temporally associated with the use of a medicinal product, whether or not considered related to the medicinal product.

Examples of AEs include:

- Exacerbation of a pre-existing illness following the start of the study.
- Increase in frequency or intensity of a pre-existing episodic event or condition.
- Condition detected or diagnosed after study drug administration even though it may have been present prior to the start of the study.

An AE **does not** include:

- Medical or surgical procedure (e.g., surgery, endoscopy, tooth extraction, transfusion); the condition that leads to the procedure may be an AE.
- Day to day fluctuations of pre-existing diseases or conditions present or detected at the start of the study that do not worsen.
- Situations where an untoward medical occurrence has not occurred (e.g., hospitalizations for cosmetic or elective surgery, social and/or convenience admissions).
- The disease or disorder being studied or sign or symptom associated with the disease or disorder unless more severe than expected for the subject’s condition.
- Overdose of either study drug or concurrent medication without any signs or symptoms.

### 8.1.2 Definition of a Serious Adverse Event

An SAE is any untoward medical occurrence that at any dose:

- Results in death.
- Is life-threatening.

  *Note:* The term “life-threatening” in the definition of “serious” refers to an event in which the subject was at risk of death at the time of the event; it does not refer to an event which hypothetically might have caused death if it were more severe.

- Requires in hospitalization or prolongation of existing hospitalization.

  *Note:* Complications that occur during hospitalization are AEs. If a complication prolongs hospitalization or fulfills any other serious criteria, the event is serious. Hospitalization for

elective treatment of a pre-existing condition that did not worsen from Baseline is not considered to be an AE.

- Results in persistent or significant disability/incapacity.

  *Note:* The term disability means a substantial disruption of a person’s ability to conduct normal life functions. This definition is not intended to include experiences of relatively minor medical significance such as uncomplicated headache, nausea, vomiting, diarrhea, influenza, or accidental trauma (i.e., sprained ankle) that may interfere or prevent everyday life functions but do not constitute a substantial disruption.

- Is a congenital anomaly/birth defect.

- Important medical events that may not be immediately life-threatening or result in death or hospitalization but may jeopardize the Subject or may require intervention to prevent one of the outcomes listed in the definition above should also usually be considered serious.

  *Note:* Examples of such events are intensive treatment in an emergency room or at home for allergic bronchospasm, blood dyscrasias, or convulsions that do not result in hospitalization; or development of drug dependency or drug abuse.

### 8.1.3 Clinical Laboratory Abnormalities and Other Abnormal Assessments

Laboratory abnormalities are usually not recorded as AEs or SAEs. All abnormal laboratory values judged to be at least possibly drug related or clinically significant abnormal laboratory results of any causality not associated with the disease under study must be repeated. The Investigator will exercise medical judgment in deciding whether abnormal laboratory values are clinically significant. In some cases, significant changes within the range of normal will require similar judgment by the Investigator.

Clinically significant abnormal laboratory values should not be listed on the AE page of the CRF unless clinical signs or symptoms are present. If an abnormal laboratory value or assessment is clearly related to a medically defined diagnosis or syndrome, the diagnosis or syndrome will be recorded on the AE page, not the individual laboratory values.

All clinically significant abnormal laboratory results or assessments will be followed until they resolve (return to normal or values before re-boost) or stabilize, or until they are judged by the Investigator to be no longer clinically significant.

### 8.1.4 Recording of Adverse Events and Serious Adverse Events

From the point of signing the informed consent form (ICF) to the first re-boost (first administration of study treatment), any worsening of an existing or new medical condition must be recorded on the Medical History CRF page. Following administration of study medication up to the Visit 10 (End of Study), any worsening of an existing or new medical condition must be recorded as an AE on the CRF. The Investigator should review all documentation (e.g., hospital progress notes, laboratory, and diagnostic reports) relative to the event being reported. The Investigator will then record all relevant

information regarding an AE/SAE on the appropriate CRF page. The Investigator will evaluate AEs using the following guidelines:

- Description of event (if the event consists of a cluster of signs and symptoms, a diagnosis should be recorded [e.g., flu syndrome] rather than each sign and symptom).
- Onset date and time.
- Stop date and time.
- Intensity should be described according to the Division of AIDS Table for Grading Severity of Adult and Pediatric Adverse Events, Version 1.0 December 2004; Clarification August 2009 (Appendix 15.1) as Mild (Grade 1), Moderate (Grade 2), Severe (Grade 3), or Life threatening (Grade 4). Intensity is defined as one of the following:
    - Mild: transient or mild discomfort; no limitation in activity; no medical intervention/therapy required.
    - Moderate: mild to moderate limitation in activity- some assistance may be needed; no or minimal medical intervention/therapy required.
    - Severe: marked limitation in activity, some assistance usually required; medical intervention/therapy required; hospitalization is possible.
    - Life-threatening: extreme limitation in activity, significant assistance required; significant medical intervention/therapy required; hospitalization or hospice care probable.

  It is important to distinguish between SAEs and severe AEs. Severity is a measure of intensity, whereas seriousness is defined by the criteria provided in Section 8.1.2. An AE of severe intensity need not necessarily be considered serious. For example, a migraine headache that incapacitates a subject for many hours may be considered a severe AE, whereas a stroke that results in a limited degree of disability may be considered mild, but should be reported as an SAE. If any parameter within a record of an AE changes, a fresh record should be made in the CRF. For example, when the severity of the AE increases, the AE should be recorded as resolved at the existing severity level and re-entered into the CRF with a new start date reflecting the first date of the increased severity.

- Seriousness (see definition of an SAE, Section 8.1.2).

  The Investigator must record whether or not the AE meets the definition of serious. If the event is serious, the Investigator must complete an SAE Report Form.

- Relationship to study drug.

  The Investigator must make a causality assessment for all AEs and must decide whether there is a reasonable possibility that the AE may have been caused by the study drug. If there is any valid reason for suspecting that there is a causal relationship between the AE and the study drug, the AE should be judged “possibly related,” “probably related,” or “definitely related”

to study drug. Unless an AE can be excluded from causality, it must be judged "possibly related" to study drug rather than "not related" to study drug.

- **Definitely Related**: An AE is considered definitely related when there is clear evidence that the event was caused by test article use. A definitely related event has a strong temporal relationship and an alternative cause is unlikely (other possible contributing factors can be ruled out).
- **Probably Related**: An AE is considered probably related when there is a reasonable possibility that the event is likely to have been caused by test article use. The AE has a timely relationship to the study procedure(s) and follows a known pattern of response, but a potential alternative cause may be present.
- **Possibly Related**: An AE is considered possibly related when there is some evidence to suggest a causal relationship or when there is a reasonable possibility that the event might have been caused by test article use. Typically, the event occurs within a reasonable time after test article use. However, other factors may have also contributed to the event, such as the subject's clinical condition or concomitant treatments.
- **Not related/Unrelated**: An AE is considered not related or unrelated when the cause of the AE is known, and the event is in no way related to any aspect of test article use.

- Outcome.

  Outcome of AEs should be recorded as resolved, resolved with sequel, not resolved, or fatal based on the status of the AE at discontinuation from the study. If an AE is not resolved at the time of discontinuation, the AE should be followed until it is resolved (returns to normal or Baseline values) or stabilized, or until it is judged by the Investigator to be no longer clinically significant.

- Action taken.
  None, required treatment, reduction of dose, or permanent discontinuation of study drug. *All applicable actions taken should be recorded*.

### 8.1.5 Follow-up of Adverse Events and Serious Adverse Events

All AEs and SAEs must be followed until they are resolved (return to normal or values before re-boosting) or stabilized, or until they are judged by the Investigator to be no longer clinically significant. Supplemental measurements and/or evaluations may be necessary to fully investigate the nature and/or causality of an AE or SAE. This may include additional laboratory tests, diagnostic procedures, or consultation with other healthcare professionals. If the subject dies, any findings (including histopathology) must be provided to the Sponsor (or designee).

### 8.1.6 Reporting of All Serious Adverse Events and Any Adverse Events Resulting in Study Discontinuation

The Investigator will promptly report all SAEs to the Sponsor (or designee) within the timeframes specified in Table 2. Prompt notification of SAEs by the Investigator is essential so that the Sponsor can meet its regulatory and ethical obligations for this study.

The Investigator should comply with the applicable local regulatory requirements related to reporting of SAEs to his/her EC.

Any SAE, regardless of expectedness or causality, must be reported by telephone and by faxing (or mailing) the completed SAE Report Form to the Sponsor (or designee) within 24 hours of the Investigator or any other site personnel's knowledge of the event. In addition, any AE resulting in permanent study discontinuation for a subject, even if not serious, must be reported immediately to the Sponsor (or designee). An updated SAE Report Form should be forwarded to the Sponsor (or designee) within 24 hours of receipt of the new/updated information. The Sponsor (or designee) will provide a list of project contacts for SAE receipt, fax numbers, telephone numbers, and mailing addresses for SAE reports.

**Table 2 Serious Adverse Event Reporting Requirements**

| | **INITIAL REPORTS** | **FOLLOW-UP REPORTS** |
|---|---|---|
| **REPORTING REQUIREMENTS** | **<u>24 hours</u>**<br>Telephone and fully completed SAE Report Form to the Sponsor (or designee) | **<u>24 hours</u>**<br>Updated SAE Report Form to the Sponsor (or designee) |

For IMPs, an AE is judged expected if its description agrees in nature, severity, frequency, and specificity with the description of events in the current Investigator's Brochure.

Safety Reports will be prepared according to the Sponsor's policies and forwarded to the Investigator as necessary. The purpose of the Safety Report is to fulfill specific regulatory and Good Clinical Practice (GCP) requirements regarding the product under investigation. When a site receives from the Sponsor (or designee) an initial or follow-up Safety Report or other safety information (e.g., a revised Investigator's Brochure), the Investigator is required to promptly notify his EC according to local guidelines, and maintain the information, as well as the documentation of the EC communication, in the site's study files.

### 8.1.7 Post-study Adverse Events or Serious Adverse Events

Investigators are not obligated to actively seek AEs or SAEs after termination of the study period. However, the Investigator should promptly notify the Sponsor (or designee) if the Investigator learns

of any SAE or death of a study subject within 30 days after a subject has been discontinued from the study, and such event(s) is (are) reasonably related to the study drug.

Investigators should promptly notify the Sponsor (or designee) if they become aware of a former study participant who is one of the parents of a subsequently conceived child with a congenital anomaly.

## 8.2 Pregnancy Information

If relevant, a urine pregnancy test will be administered at Visit 2 and Visit 3 before re-boosting. If any female subject becomes pregnant during the study, the Investigator must notify the Sponsor (or designee) by telephone within 24 hours of learning about the pregnancy and report it in the electronic CRF (eCRF). The eCRF must be completed within 14 days. The Investigator must diligently follow the subject until delivery or termination of the pregnancy, providing necessary updated information to the Sponsor (or designee). Any premature termination of the pregnancy will also be reported.

Although pregnancy occurring in a clinical study is not considered to be an AE or SAE, any pregnancy complication or elective termination of a pregnancy for medical reasons will be recorded as an AE or SAE and will be followed as such. A spontaneous abortion is always considered to be an SAE.

# 9 DATA COLLECTION

## 9.1 Data Collection

Subject data will be collected on eCRF and will be substantiated by subject date of birth, the subject identification number in the Study CT-BI Vacc-4x 2007/1 study and study number. The eCRF will be completed according to guidelines provided by the Sponsor (or designee) in writing and/or verbally. All required data are to be recorded on the eCRF.

Completed eCRFs will be reviewed by the study monitor in line with eCRF review guidelines for the study to ensure completeness and consistency. Data will be collected using the Aptiv Advantage® system, a web-based electronic data capture technology, which is based on an Oracle® platform. The Sponsor (or designee) will subject all eCRF data to 100% source verification and audit to ensure adequate quality control and assurance of subject data. Any discrepancies found during the eCRF review are to be clarified by the Investigator. This includes eCRF reviews at the site by the Sponsor (or designee), or during quality assurance review of the data.

The Investigator (or designee) must record all required subject data using the previously specified data collection method defined by the Sponsor. An explanation must be documented for any missing data. The Investigator must sign and date a declaration on the eCRF attesting to his/her responsibility for the quality of all data recorded, and that the data represents a complete and accurate record of each subject's participation in the study. Each subject eCRF will be retained by the Investigator on a CD.

## 9.2 Data Processing

Subject eCRFs will be completed from source documents by designated site staff in line with eCRF completion guidelines. The on-going status of data entry will be reflected in the EDC system and will be accessible to all users based on their access privileges.

Data within the eCRFs will be source data verified (100 %) and the EDC system will be updated to indicate which data items have been verified. In the event that changes are made to the eCRF after verification by the CRA, the CRA will be required to re-verify the data.

The eCRF will be approved by the Investigator after it has been verified and all discrepancies resolved. In the event that changes are made to the eCRF after approval by the Investigator, the eCRF must be re-approved.

The eCRF will be validated through extensive data checking and query processing capabilities within the EDC system. Electronic messaging (discrepancy routing) between users will forward discrepancies within the EDC system from one user group to another depending on the action required at the time. A full audit trail of any changes made to the eCRF will also be stored within the system.

# 10 STATISTICAL METHODS AND PLANNED ANALYSES

## 10.1 Determination of Sample Size

Eighty-eight subjects completed the initial study CT-BI Vacc-4x 2007/1 (EudraCT Number 2007-006302-13) immunization regimen with active Vacc-4x and stopped ART at Week 28. It is estimated that approximately 30 to 40 of these subjects will be eligible for this follow-up, re-boosting study.

## 10.2 Randomization Codes

This is a non-randomized study.

## 10.3 Populations to be Analyzed

The following populations will be used in the analyses:

Safety: All subjects who receive at least one re-boosting immunization will be included within the safety population. All safety analyses will be conducted on the safety population.

Intention to treat (ITT): All subjects who receive at least one re-boosting immunization and have any post-baseline efficacy data will be included in the ITT population. Unless otherwise indicated, all efficacy analyses will be conducted on the ITT Population.

Per protocol (PP): All ITT subjects who receive two re-boosting immunizations, discontinue ART at Visit 5 (as planned) and who do not incur a major protocol violation that would challenge the validity of their data will be included within the PP population. All efficacy analyses will also be conducted on the PP population.

## 10.4 Interim Analysis

No interim analysis is planned.

## 10.5 Subject Accountability

The number of subjects in the study will be summarized, both overall and by study center. The number of subjects in each of the Safety, ITT and PP populations will be summarized. In addition, the number of subjects who discontinued ART at Visit 5, and re-started ART at Visit 9 will also be summarized. Any subjects who discontinued/re-started ART at different scheduled visits will be described. In addition, the number of subjects completing / not-completing the final follow-up Visit 10 (end of study) assessment will be summarized along with the primary reason for early withdrawal from the study.

### 10.5.1 Protocol Deviations

Subject data will be reviewed for major protocol deviations by a qualified clinical reviewer at a data review meeting to be held prior to database lock. Subjects with any major protocol deviations will be excluded from the PP population. Protocol deviations will be listed by subject.

### 10.5.2 Subgroup Analyses

For the reporting of efficacy endpoints subgroup analyses will be performed. The definition of subgroups (either certain subjects excluded or part of their data excluded) will be based on the timing of when a subject is on or off ART during their participation in the study. This will be further documented within the Statistical Analysis Plan.

## 10.6 Statistical Methods

In general continuous variables will be described using number of observations (n), mean, standard deviation (SD), geometric mean, minimum, maximum, median, and quartiles. Categorical data will be presented using counts (n) and percentages (%).

All statistical analyses will be performed using appropriate procedures in SAS Software Version 9.2 or higher. Significance of effects will be determined by two-sided tests with p-value $< 0.05$.

All analyses will be carried out after all participants have completed Visit 10 (or early withdrawal from study) and the study database has been authorized by the Sponsor as complete and final, and protocol deviations have been identified.

### 10.6.1 Analysis of Demographic and Subject Characteristics

Descriptive statistics of the demographic profile, baseline conditions, and subject disposition will be summarized.

### 10.6.2 Efficacy Analysis

#### 10.6.2.1 Primary Efficacy Endpoints

The primary efficacy endpoint of this study is the comparison of the re-boost viral load set-point (definition given in Section 2.2.1.1) after re-boosting and a 16 week ART-free period with the viral load set-point (definition given in Section 2.2.1.1) reported in the initial CT-BI Vacc-4x 2007/1 study (EudraCT Number 2007-006302-13). The primary comparison will be a Wilcoxon signed rank test comparing changes in viral load between the two stated comparison values. The re-boost viral load set-point will also be compared to the pre-ART viral load value, if available from the study CT-BI Vacc-4x 2007/1.

Note: For the reporting of this viral load endpoint, viral load values may have an appropriate transformation (e.g. log base 10) applied prior to endpoint derivation and subsequent analysis.

The co-primary endpoint of this study is the T-cell response (binary outcome: response Yes/No) to Vacc-4x and/or p24 which will be measured by ELISPOT as well as by T-cell proliferative response assay (flow cytometry) and intracellular cytokine staining at Visit 2 (before re-boosting), Visit 4 (two weeks after re-boosting), Visit 6, 9 and 10 (End of Study). These responses will be compared to the T-cell response reported in the initial study CT-BI Vacc-4x 2007/1 (EudraCT Number 2007-006302-13) using McNemar’s test. Definition of positive responses will be defined in the statistical analysis plan (SAP).

#### 10.6.2.2 Secondary Efficacy Endpoints

The following endpoints (based on continuous data types) will be compared (for within subject comparisons) using the Wilcoxon signed rank test:

- CD4 counts at all visits, absolute numbers, and treatment emergent changes from the mean of Screening and Visit 2 data.
- Change (and percent change) in CD4 count from Visit 5 (discontinue ART) to Visit 6, 7, 8, 9 and 10 (End of Study).
- CD8 counts at all visits, absolute numbers, and treatment emergent changes from the mean of Screening and Visit 2 data.
- Change (and percent change) in CD8 count from Visit 5 (discontinue ART) to Visit 6, 7, 8, 9 and 10 (End of Study).

For each individual subject both the absolute and relative changes in CD4 and CD8 values registered at Visit 9) will be compared with the corresponding values registered at Week 28 (ART discontinued) and Week 52 (or last visit before ART re-initiation) in the initial study CT-BI Vacc-4x 2007/1 (EudraCT Number 2007-006302-13).

- DTH (both induration and erythema) at Visit 2 and Visit 4, in terms of observed areas ($mm^2$ length x height), areas at Visit 2 will be compared with the corresponding values registered at Visit 4. In addition the values for these DTH parameters at Visit 4 will be compared with the DTH responses reported at Week 18 in the initial study CT-BI Vacc-4x 2007/1 (EudraCT Number 2007-006302-13).

The following endpoints (based on binary data types) will be compared (for within subject comparisons) using McNemar’s test:

- DTH (both induration and erythema) at Visit 2 and Visit 4, in terms of a positive test, defined as an area $\geq 10mm^2$., The number of positive tests registered at Visit 2 will be compared with the corresponding values registered at Visit 4. In addition the values for these DTH parameters at Visit 4 will be compared with the DTH responses reported at Week 18 in the initial study CT-BI Vacc-4x 2007/1 (EudraCT Number 2007-006302-13).

### 10.6.3 Safety Analyses

Safety will be assessed through the collection of AE reports, assessing virologic suppression, vital signs, and laboratory evaluations that are commonly employed in clinical studies. Week 1 safety data will be used for comparison with the data obtained during the study period. Summary statistics will be prepared for measured values and change from Visit 2 values. Virologic suppression will be assessed between the timing of ART interruption and ART re-initiation. Summaries of treatment-emergent clinically important abnormalities in vital signs and laboratory data will be provided. No statistical testing will be performed on safety parameters.

#### 10.6.3.1 Adverse Events

For presentation, AE verbatim text will be coded into a Medical Dictionary for Regulatory Activities (MedDRA) term, and classified by System Organ Class (SOC) and preferred term. AEs will then be summarized and grouped by SOC and preferred term. Results will be displayed in order of decreasing frequency, both across SOC and within each SOC term. AEs with onset on or after the start of study medication are considered treatment-emergent. AEs reported prior to treatment administration will be reported separately from treatment-emergent AEs.

In addition, summaries will be provided by incidence, severity (mild, moderate, severe), and relationship to study medication (Related, Unrelated). "Possibly Related", "Probably Related" and "Definitely Related" or relationship is missing will be grouped as “Related.”

SAEs and AEs leading to discontinuation will be listed.

#### 10.6.3.2 Virologic Suppression

The number and percentage of subjects who experience similar virologic suppression (HIV RNA < 50 copies/ml) 8 weeks after reinitiation of ART (Visit 10) as before ART interruption (Visit 5) will be summarized.

#### 10.6.3.3 Vital Signs and Laboratory Parameters

Descriptive statistics of sitting systolic and diastolic blood pressure (mmHg), heart rate (bpm), and body weight (kg), absolute values and change from Visit 2, will be presented for each scheduled visit at which they were measured. Subject listings of all vital sign and body weight data collected during the study will also be presented.

Laboratory results (absolute values and treatment emergent changes from mean of Screening and Visit 2) will be summarized for blood chemistry and hematology parameters. Laboratory values that are below the level of quantification (BLQ) will be set to ‘0’ in computations for the aggregate analyses but will be noted as below the detection limit in the listings. Laboratory values that are missing will remain missing but will be noted as such in listings.

#### 10.6.3.4 Physical Examinations

Abnormal physical examination findings at Visit 2, throughout the study, and at Visit 10 (End of Study) will be summarized, both overall and by body system.

#### 10.6.3.5 Concurrent Medications

A summary of all concomitant medications taken during the course of the study will be presented in tabular form by therapeutic drug class and generic drug name using the World Health Organization (WHO) Drug classification. All concomitant medications will be detailed in the subject data listings.

# 11 STUDY MANAGEMENT AND ETHICAL AND REGULATORY REQUIREMENTS

## 11.1 Regulatory Approval and Good Clinical Practice

This study will be conducted in accordance with GCP requirements described in the current revision of International Conference on Harmonization (ICH) of Technical Requirements of Pharmaceuticals for Human Use Guidelines and all applicable regulations. Compliance with these regulations and guidelines also constitutes compliance with the ethical principles originating from the Declaration of Helsinki. This study will also be carried out in accordance with local legal requirements.

Before the first subject is screened in the study, all ethical and legal requirements must be met.

The study is commercially funded by the Sponsor, Bionor Immuno AS, and there are no identified institutional affiliations or potential conflicts of interest. All subjects in this study will have completed the initial study CT-BI Vacc-4x 2007/1 (EudraCT Number 2007-006302-13) and are well aware of the treatment. The Sponsor has appropriate medical insurance and will pay medical expenses for treatment or injury incurred by subjects as a consequence of participating in this study. Medical treatments available will be in accordance with standard clinical practice. Compensation for any injury caused by taking part in this study will be in accordance with the guidelines of the Association

of the British Pharmaceutical Industry (ABPI). The ABPI guidelines recommend that the Sponsor, without legal commitment, should compensate subjects on a “balance of probabilities” basis for an injury that is likely to have resulted from giving Vacc-4x, Leukine® or any other procedure carried out in accordance with the approved protocol. The Sponsor will not compensate subjects where such injury results from any procedure carried out which is not in accordance with the approved protocol. A subject’s right by law to claim compensation for injury where they can prove negligence is not affected.

## 11.2 Deviations from the Protocol and Protocol Amendments

A copy of the approved protocol and the curriculum vitae of the Investigator, together with other required documentation, will be filed with the appropriate regulatory authorities. If it is necessary to amend the protocol during the study, proper notification in accordance with all applicable regulations, will be made to the regulatory authorities and IRBs/IECs in the form of a protocol amendment. Any protocol or other deviations that occur during the study will be documented and reported to the Sponsor. Depending on the nature of the deviation, this may be reported to the appropriate regulatory authority.

Neither the Investigator nor the Sponsor will alter this study protocol without obtaining the written agreement of the other. Once the study has started, amendments should be made only in exceptional cases. The changes then become part of the study protocol.

## 11.3 Discontinuation of Study

The Sponsor reserves the right to discontinue the study for any reason at any time. In addition, the study may be stopped at any time if, in the opinion of the Investigator or Sponsor, safety data suggest that the medical safety of subjects is being compromised.

## 11.4 End of Study

The End of the Study is defined as last subject last visit (Visit 10).

## 11.5 Study Records Retention and Direct Access to Source Documents

The Investigator must retain essential documents until notified by the Sponsor. No study document will be destroyed without prior written agreement between the Sponsor and the Investigator. Should the Investigator wish to assign the study records to another party or move them to another location, written agreement must be obtained from the Sponsor.

The Investigator must maintain a copy of all data collected for each subject treated (including CRF and source data). eCRF data will be stored on a CD and provided to the Investigator by the Sponsor. In order to assure the accuracy of data collected in the eCRF, it is mandatory that representatives of the Sponsor (or designee) as well as representatives of regulatory authorities and IRBs/IECs have direct access to original source documents (e.g., subject records, subject charts, laboratory reports). During the review of these documents, the anonymity of the subject will be respected with strict adherence to professional standards of confidentiality.

The Sponsor reserves the right to terminate the study for refusal of the Investigator to supply source documentation of work performed in this clinical study.

Records that must be retained by the Investigator include, but are not limited to:

- Signed informed consent documents for all subjects.
- Subject identification code list, screening log (if applicable), and enrollment log.
- Record of all relevant communications between the Investigator and the IRB/IEC.
- Composition of the IRB/IEC.
- Record of all relevant communications between the Investigator and the Sponsor (or designee).
- List of Sub-Investigators and other appropriately qualified persons to whom the Investigator has delegated significant study related duties, together with their roles in the study and their signatures.
- Copies of documentation of corrections for all subjects.
- CD containing eCRF data
- Drug accountability records.
- Record of any body fluids or tissue samples retained.
- All other source documents (subject records, hospital records, laboratory records, etc.).

All other documents as listed in Section 8 of the ICH consolidated guideline on GCP (Essential Documents for the Conduct of a Clinical Trial).

## 11.6 Investigator Responsibilities

### 11.6.1 Subject Information and Informed Consent

Before being admitted to the clinical study, all subjects (and their caregivers, if applicable) must consent in writing to participate. An ICF and Subject Information Sheet will be given to each subject as appropriate, which will contain all regulatory required elements, all ICH required elements, and appropriate data protection information, when applicable, in language that is understandable to the subject. The consent should note that the Investigator is receiving compensation for the expenses of conducting the study.

The process of obtaining the informed consent will be in compliance with all regulations, ICH requirements, and local laws.

The Investigator will review the study with each subject and caregiver (if applicable). The review will include the nature, scope, procedures, and possible consequences of the subject's participation in the study. The consent and review must be in a form understandable to the subject. The Investigator (or designee) and the subject must both sign and date the ICF after review and before the subject can

participate in the study. The subject will receive a copy of the signed and dated form, and the original will be retained in the site study files. The Investigator (or designee) must emphasize to the subject that study participation is entirely voluntary and that consent regarding study participation may be withdrawn at any time without penalty or loss of benefits to which the subject is otherwise entitled.

If the ICF is amended during the study, the Investigator must follow all applicable regulatory requirements pertaining to approval of the amended ICF by the IRB/IEC. The site must use the amended consent form for all new subjects and repeat the consent process with the amended ICF for any ongoing subjects.

### 11.6.2 Institutional Review Board/Independent Ethics Committee Approval and Other Institutional Requirements

Before the start of the study, the study protocol, informed consent document, and any other appropriate documents will be submitted to the IRB/IEC with a cover letter or a form listing the documents submitted, version numbers and dates, and the site (or region or area of jurisdiction, as applicable) for which approval is sought. Per institutional/local regulatory requirements, the study protocol and any other appropriate documents will be submitted to scientific committees for approval.

The Investigator will forward to the Sponsor (or designee) a copy of the IRB's/IEC's approval of this protocol, amendments, ICF and any changes to the informed consent, and any other submitted documentation that requires approval. The Investigator will also keep documentation of study approval by internal scientific committees per institutional/local regulatory requirements.

Study drug can only be supplied to the Investigator after documentation of all ethical and legal requirements for starting the study has been received by the Sponsor.

While the study is ongoing and at study completion/discontinuation, the Investigator must submit to the IRB/IEC the following information, as required by local regulations:

- Information on serious or unexpected Adverse Drug Reactions showing due diligence in providing this information as soon as possible.
- Periodic safety reports.
- Periodic reports on the progress of the study.
- Notification of the end of study or early termination of the study.
- Final Study Summary upon study completion or closure.
- Other information as required by local regulations.

#### 11.6.3 Curriculum Vitae

The Investigator must provide the Sponsor (or designee) with current curricula vitae for herself/himself and each Sub-Investigator. A copy of the current license for each clinician, where issued, must also be provided.

#### 11.6.4 Laboratory Certification and Normal Values

In this study central laboratories will be used. For clinical chemistry the Covance laboratory in Geneva, Switzerland will be used for the European sites, while a US Covance laboratory in Indianapolis will be used for the US sites. All immunological analysis will be performed at the University of Lausanne, Switzerland. The laboratories will provide the Sponsor (or designee) with the laboratory certification and normal values for all laboratory tests required by protocol.

#### 11.6.5 Delegation of Investigator Responsibilities

The Investigator should ensure that all persons assisting with the study are adequately informed about the protocol, any amendments to the protocol, the study drug, and their study related duties and functions. The Investigator should maintain a list of sub-Investigators and other appropriately qualified persons to whom he or she has delegated significant study related duties.

#### 11.6.6 Liability and Insurance

Liability and insurance provisions for this study are specified in the Investigator's contract.

### 11.7 Study Monitoring and Auditing

In accordance with applicable regulations, GCP, and the procedures of the Sponsor (or designee), the study monitor will periodically contact the site and conduct on-site visits. The extent, nature, and frequency of on-site visits will be based on study complexity, enrollment rate, and data quality at the site. Through frequent communications (e.g., letter, e-mail, telephone), the study monitor will ensure that the investigation is conducted according to protocol and regulatory requirements.

During these contacts, the monitoring activities will include:

- Checking and assessing the progress of the study.
- Reviewing study data collected to date for completeness and accuracy.
- Conducting source document verification by reviewing each subject’s CRF against source documents (e.g., medical records, laboratory result reports, raw data collection forms).
- Identifying any issues and addressing resolutions.

These activities will be done in order to verify that the:

- Data are authentic, accurate, and complete.

- Safety and rights of the subjects are being protected.
- Study is conducted in accordance with the currently approved protocol (and any amendments), GCP, and all applicable regulatory requirements.

The Investigator will allow the study monitor direct access to all relevant documents, and allocate his/her time and the time of his/her staff to the study monitor to discuss findings and any relevant issues.

In addition to contacts during the study, the study monitor will contact the site prior to the start of the study to discuss the protocol and data collection procedures with site personnel.

At study closure, study monitors will conduct all activities as indicated in Section 11.9.

## 11.8 Quality Assurance

The Sponsor (or designee) may conduct a quality assurance audit of this study at any time. The Sponsor's (or designee's) auditing procedures will be followed in order to comply with GCP guidelines and ensure acceptability of the study data for registration purposes. If such an audit occurs, the Investigator will give the auditor direct access to all relevant documents, and will allocate his/her time and the time of his/her staff to the auditor as may be required to discuss findings and any relevant issues.

In addition, regulatory authorities may conduct an inspection of this study. If such an inspection occurs, the Investigator will allow the inspector direct access to all source documents, CRFs, and other study documentation for source data check and/or on-site audit inspection. The Investigator must allocate his/her time and the time of his/her staff to the inspector to discuss findings of any relevant issues.

## 11.9 Study Termination and Site Closure

Upon completion of the study, the following activities, when applicable, must be conducted by the study monitor in conjunction with the Investigator, as appropriate:

- Return of all study data to the Sponsor.
- Data clarifications and/or resolutions.
- Accounting, reconciliation, and final disposition of used and unused study drug.
- Review of site study records for completeness.

In addition, the Sponsor reserves the right to temporarily suspend or prematurely terminate this study for any reason.

If the study is suspended or terminated for safety reasons, the Sponsor will promptly inform the Investigator, and will also inform the regulatory authorities of the suspension or termination of the

study and the reasons for the action. The Investigator is responsible for promptly informing the IRB/IEC, and providing the reasons for the suspension or termination of the study.

If the study is prematurely terminated, all study data must be returned to the Sponsor. In addition, the site must conduct final disposition of all unused study drugs in accordance with the Sponsor procedures for the study.

## 11.10 Site Termination

The Sponsor may at any time, at its sole discretion, terminate the study site for various reasons, including, but not limited to, the following:

- Failure of the Investigator to enroll subjects into the study at a reasonable rate.
- Failure of the Investigator to comply with applicable laws and/or pertinent regulations.
- Submission of knowingly false information from the research facility to the Sponsor, study monitor, or regulatory authorities.
- Insufficient adherence to protocol requirements.

If the participation of the study site is terminated for reasons other than safety, the Sponsor will issue a written notice to the Investigator. The written notice will contain the reasons for taking such action. If the study site is terminated for non-compliance, appropriate regulatory authorities will also be notified by the Sponsor.

# 12 DISCLOSURE OF DATA

## 12.1 Confidentiality

Subject names will remain confidential and will not be included in the database supplied to the Sponsor (or designee). Only the subject number for this study, the subject identification number for the previous study (Study CT-BI Vacc-4x 2007/1), and year of birth will be recorded on the CRF. If the subject name appears on any other document collected (e.g., hospital discharge summary), the name must be obliterated before the document is transmitted to the Sponsor (or designee). All study findings will be stored in electronic databases. The subjects will give explicit permission for representatives of the Sponsor, regulatory authorities, and the IRB/IEC to inspect their medical records to verify the information collected. Subjects will be informed that all personal information made available for inspection will be handled in the strictest confidence and in accordance with appropriate laws and regulations. All personnel involved in the study will observe or work within the confines of the local data protection regulations.

The Investigator will maintain a personal subject identification list (subject and treatment numbers with the corresponding subject names) to enable records to be identified.

## 12.2 Publication

All information concerning the product as well as any information such as clinical indications for the drug, its formula, methods of manufacture and other scientific data relating to it, that have been provided by the Sponsor (or designee), and are unpublished, are confidential and must remain the sole property of the Sponsor. The Investigator will agree to use the information only for the purposes of carrying out this study and for no other purpose unless prior written permission from the Sponsor is obtained. The Sponsor has full ownership of the CRFs completed as part of the study.

By signing the study protocol, the Investigator agrees that the results of the study may be used for the purposes of national and international registration, publication, and information for medical and pharmaceutical professionals by the Sponsor. If necessary, the authorities will be notified of the Investigator's name, address, qualifications, and extent of involvement.

The Sponsor (or designee) will prepare a final report on the study. The Investigator may not publish or present any information on this study without the express written approval of the Sponsor. Additionally, the Sponsor, may, for any reason, withhold approval for publication or presentation.

# 13 INVESTIGATOR’S PROTOCOL AGREEMENT

The Investigator Protocol Agreement at the front of this document must be signed by the Investigator. An original or a copy must be kept on file with the Sponsor, and the Investigator must retain an original or a copy. The completed protocol agreement signifies review and acceptance of the protocol by the Investigator prior to initiation of the study.

## 14 REFERENCES

Arellano M, K Waller E. 2004. Granulocyte-macrophage-colony-stimulating factor and other cytokines: as adjuncts to cancer immunotherapy, stem cell transplantation, and vaccines. Curr Hematol Rep. 3(6):424-31.

Åsjö B, Stavang H, Sorensen B, Baksaas I, Nyhus J, Langeland N. 2002. Phase I trial of a therapeutic HIV type 1 vaccine, Vacc-4x, in HIV type 1-infected individuals with or without antiretroviral therapy. AIDS Res Hum Retroviruses. 18(18):1357-65.

Banchereau J, Steinman RM. 1998. Dendritic cells and the control of immunity. Nature. 392(6673):245-52.

Bernhardt SL, Gjertsen MK, Trachsel S, Moller M, Eriksen JA, Meo M, Buanes T, Gaudernack G. 2006. Telomerase peptide vaccination of patients with non-resectable pancreatic cancer: A dose escalating phase I/II study. Br. J. Cancer. 95(11):1474-82.

Brunsvig PF, Aamdal S, Gjertsen MK, Kvalheim G, Markowski-Grimsrud CJ, Sve I, Dyrhaug M, Trachsel S, Moller M, Eriksen JA, Gaudernack G. 2006. Telomerase peptide vaccination: a Phase I/II study in patients with non-small cell lung cancer. Cancer Immunol Immunother. 55(12):1553-64.

Clinical Study Report 2001-06-01 of CTN B-HIV-1/99 Immunotherapy of HIV-infected patients.

Clinical Study Report 2005-09-26 of CTN B-HIV-1/2001 Immunotherapy of HIV-infected patients.

Clinical Study Report 2011-02-28 of CTN BI/Vacc-4x/2009/1 Immunotherapy of HIV-infected patients.

Covance Report No. 1638/3 – D6144, 1999. Vacc-4x: Single Dose Intravenous Toxicity Study in the Rat.

Covance Report No. 1638/4 – D6144, 1999. Vacc-4x: Single Dose Intravenous Toxicity Study in the Mouse.

Covance Report No. 1638/010, 2008. Vacc-4x: 22 Week Intermittent Intradermal Administration Toxicity Study in the Rabbit.

Covance Report No. 1638/011, 2008. Vacc-4x: 4 Week Intermittent Intradermal Administration Toxicity Study in the Rabbit, with a 4-week Treatment-free Period.

Disis ML, Bernhard H, Shiota FM, Hand SL, Gralow JR, Huseby ES, Gillis S, Cheever MA. 1996. Granulocyte-macrophage colony-stimulating factor: an effective adjuvant for protein and peptide-based vaccines. Blood. 88(1):202-10.

Dyer WB, Zaunders JJ, Yuan FF, Wanng B, Learmont JC, Geczy AF, Saksena NK, McPhee DA, Gorry PR, Sullivan JS. 2008. Mechanisms of HIV non-progression; robust and sustained CD4 T-cell proliferative responses to p24 antigen correlate with control of viraemia and lack of disease progression after long-term transfusion-acquired HIV-1 infection. Retrovirology 5:112

Forster SM, Osborne LM, Cheinsong-Popov R, Kenny S, Burnell R, Jeffries DJ, Jeffries DJ, Pinching AJ, Harris JR, Weber JN. 1987. Decline of anti-p24 antibody precedes antigenaemia as correlate of prognosis in HIV-1 infection. AIDS. 1(4):235-40.

Gjertsen MK, Buanes T, Rosseland AR, Bakka A, Gladhaug I, Soreide O, Eriksen JA, Moller M, Baksaas I, Lothe RA, Seterdal I, Gaudernack G. 2001. Intradermal ras peptide vaccination with granulocyte-macrophage colony-stimulating factor as adjuvant: Clinical and immunological responses in patients with pancreatic adenocarcinoma. Int J Cancer. 92(3):441-50.

Hodge JW, Greiner JW, Tsang KY, Sabzevari H, Kudo-Saito C, Grosenbach DW, Gulley JL, Arlen PM, Marshall JL, Panicali D, Schlom J. 2006. Costimulatory molecules as adjuvants for immunotherapy. Front Biosci. 11:788-803.

Jacobson JM, Lederman MM, Spritzler J, Valdez H, Tebas P, Skowron G, Wang R, Jackson JB, Fox L, Landay A, Gilbert MJ, O'Neil D, Bancroft L, Al-Harthi L, Jacobson MA, Merigan TC Jr, Glesby MJ. 2003. National Institute of Allergy and Infectious Diseases AIDS Clinical Trials Group. Granulocyte-macrophage colony-stimulating factor induces modest increases in plasma human immunodeficiency virus (HIV) type 1 RNA levels and CD4+ lymphocyte counts in patients with uncontrolled HIV infection. J Infect Dis. 188(12):1804-14.

Kran AM, Sorensen B, Nyhus J, Sommerfelt MA, Baksaas I, Bruun JN, Kvale D. 2004. HLA- and dose-dependent immunogenicity of a peptide-based HIV-1 immunotherapy candidate (Vacc-4x). AIDS. 18(14):1875-83.

Kran A-M. B. et al. Reduced viral burden amongst high responder patients following HIV-1 p24 peptide-based therapeutic immunisation. Vaccine 2005 23:4011-4015.

Kran A-M. B. et al. Long-term HIV-specific responses and delayed resumption of antiretroviral therapy after peptide immunization targeting dendritic cells. AIDS 2006 20:627-630.

Kvale D, Kran AM, Sommerfelt MA, Nyhus J, Baksaas I, Bruun JN, Sorensen B. 2005. Divergent in vitro and in vivo correlates of HIV-specific T-cell responses during onset of HIV viraemia. AIDS. 19(6):563-67.

Lind A. et al. Long-term proliferative CD4 and CD8 T cell memory 7 years after intradermal immunizations against short HIV Gag p24-like peptides targeting dendritic cells. AIDS 2010.

Lind A. et al. Intradermal vaccination of HIV-infected patients with short HIV Gag p24-like peptides induces CD4 and CD8 T cell responses lasting more than 7 years. Scand J Inf Dis 2012 :1-7.

Rockstroh J.K. et al. A phase II, randomized, double-blind, multicenter, immunogenicity study of Vacc-4x versus placebo in patients infected with HIV-1 who have maintained an adequate response to ART. IAS 2011.

Sasaki MG, Foccacia R, de Messias-Reason IJ. 2003. Efficacy of granulocyte-macrophage colony-stimulating factor (GM-CSF) as a vaccine adjuvant for hepatitis B virus in patients with HIV infection. Vaccine. 21(31):4545-9.

Sommerfelt M, Nyhus J, Sørensen B. 2004. Novel peptide-based HIV-1 immunotherapy. Expert Opinion on Biological Therapy. 4 (3):349-61.

Sommerfelt M. et al. Comparing $CD4^+$ T-cell decline during treatment interruption in HIV-1-infected patients who did not receive the candidate immunotherapy Vacc-4x. Presented at XVI International AIDS Conference, Toronto Canada, 13-18 August 2006.

Weber JN, Clapham PR, Weiss RA, Parker D, Roberts C, Duncan J, Weller I, Carne C, Tedder RS, Pinching T, Chiengsong-Popov R. 1987. Human immunodeficiency virus infection in two cohorts of homosexual men: neutralising sera and association of anti-gag antibody with prognosis. Lancet. 1:119-22.

# 15 APPENDICES

## 15.1 Division of AIDS Table for Grading the Severity of Adult and Pediatric Adverse Events, Version 1.0 December 2004; Clarification August 2009

# DIVISION OF AIDS TABLE FOR GRADING THE SEVERITY OF ADULT AND PEDIATRIC ADVERSE EVENTS
## VERSION 1.0, DECEMBER, 2004; CLARIFICATION AUGUST 2009

The Division of AIDS Table for Grading the Severity of Adult and Pediatric Adverse Events ("DAIDS AE Grading Table") is a descriptive terminology which can be utilized for Adverse Event (AE) reporting. A grading (severity) scale is provided for each AE term.

This clarification of the DAIDS Table for Grading the Severity of Adult and Pediatric AE's provides additional explanation of the DAIDS AE Grading Table and clarifies some of the parameters.

### I. Instructions and Clarifications

Grading Adult and Pediatric AEs

The DAIDS AE Grading Table includes parameters for grading both Adult and Pediatric AEs. When a single set of parameters is not appropriate for grading specific types of AEs for both Adult and Pediatric populations, separate sets of parameters for Adult and/or Pediatric populations (with specified respective age ranges) are given in the Table. If there is no distinction in the Table between Adult and Pediatric values for a type of AE, then the single set of parameters listed is to be used for grading the severity of both Adult and Pediatric events of that type.

> **Note:** In the classification of adverse events, the term "**severe**" is not the same as "**serious**." Severity is an indication of the intensity of a specific event (as in mild, moderate, or severe chest pain). The term "**serious**" relates to a participant/event outcome or action criteria, usually associated with events that pose a threat to a participant's life or functioning.

Addenda 1-3 Grading Tables for Microbicide Studies

For protocols involving topical application of products to the female genital tract, male genital area or rectum, strong consideration should be given to using Appendices I-III as the primary grading scales for these areas. The protocol would need to specifically state that one or more of the Appendices would be primary (and thus take precedence over the main Grading Table) for items that are listed in both the Appendix and the main Grading Table.

- Addendum 1 - Female Genital Grading Table for Use in Microbicide Studies - PDF
- Addendum 2 - Male Genital Grading Table for Use in Microbicide Studies - PDF
- Addendum 3 - Rectal Grading Table for Use in Microbicide Studies - PDF

Grade 5

For any AE where the outcome is death, the severity of the AE is classified as Grade 5.

Estimating Severity Grade for Parameters Not Identified in the Table

In order to grade a clinical AE that is not identified in the DAIDS AE grading table, use the category "Estimating Severity Grade" located on Page 3.

Determining Severity Grade for Parameters "Between Grades"

If the severity of a clinical AE could fall under either one of two grades (e.g., the severity of an AE could be either Grade 2 or Grade 3), select the higher of the two grades for the AE. If a laboratory value that is graded as a multiple of the ULN or LLN falls between two grades, select the higher of the two grades for the AE. For example, Grade 1 is 2.5 x ULN and Grade 2 is 2.6 x ULN for a parameter. If the lab value is 2.53 x ULN (which is between the two grades), the severity of this AE would be Grade 2, the higher of the two grades.

Values Below Grade 1

Any laboratory value that is between either the LLN or ULN and Grade 1 should not be graded.

**DIVISION OF AIDS TABLE FOR GRADING THE SEVERITY OF ADULT AND PEDIATRIC ADVERSE EVENTS**
**VERSION 1.0, DECEMBER, 2004; CLARIFICATION AUGUST 2009**

Determining Severity Grade when Local Laboratory Normal Values Overlap with Grade 1 Ranges
In these situations, the severity grading is based on the ranges in the DAIDS AE Grading Table, even when there is a reference to the local lab LLN.

*For example: Phosphate, Serum, Low, Adult and Pediatric > 14 years (Page 20) Grade 1 range is 2.50 mg/dL - < LLN.* A particular laboratory's normal range for Phosphate is 2.1 – 3.8 mg/dL. A participant's actual lab value is 2.5. In this case, the value of 2.5 exceeds the LLN for the local lab, but will be graded as Grade 1 per DAIDS AE Grading Table.
.

**II. Definitions of terms used in the Table:**

| | |
|---|---|
| Basic Self-care Functions | Adult<br>Activities such as bathing, dressing, toileting, transfer/movement, continence, and feeding.<br><br>Young Children<br>Activities that are age and culturally appropriate (e.g., feeding self with culturally appropriate eating implement). |
| LLN | Lower limit of normal |
| Medical Intervention | Use of pharmacologic or biologic agent(s) for treatment of an AE. |
| NA | Not Applicable |
| Operative Intervention | Surgical OR other invasive mechanical procedures. |
| ULN | Upper limit of normal |
| Usual Social & Functional Activities | Adult<br>Adaptive tasks and desirable activities, such as going to work, shopping, cooking, use of transportation, pursuing a hobby, etc.<br><br>Young Children<br>Activities that are age and culturally appropriate (e.g., social interactions, play activities, learning tasks, etc.). |

## DIVISION OF AIDS TABLE FOR GRADING THE SEVERITY OF ADULT AND PEDIATRIC ADVERSE EVENTS
## VERSION 1.0, DECEMBER, 2004; CLARIFICATION AUGUST 2009

| PARAMETER | GRADE 1 MILD | GRADE 2 MODERATE | GRADE 3 SEVERE | GRADE 4 POTENTIALLY LIFE-THREATENING |
|---|---|---|---|---|
| **ESTIMATING SEVERITY GRADE** | | | | |
| Clinical adverse event NOT identified elsewhere in this DAIDS AE Grading Table | Symptoms causing no or minimal interference with usual social & functional activities | Symptoms causing greater than minimal interference with usual social & functional activities | Symptoms causing inability to perform usual social & functional activities | Symptoms causing inability to perform basic self-care functions OR Medical or operative intervention indicated to prevent permanent impairment, persistent disability, or death |
| **SYSTEMIC** | | | | |
| Acute systemic allergic reaction | Localized urticaria (wheals) with no medical intervention indicated | Localized urticaria with medical intervention indicated OR Mild angioedema with no medical intervention indicated | Generalized urticaria OR Angioedema with medical intervention indicated OR Symptomatic mild bronchospasm | Acute anaphylaxis OR Life-threatening bronchospasm OR laryngeal edema |
| Chills | Symptoms causing no or minimal interference with usual social & functional activities | Symptoms causing greater than minimal interference with usual social & functional activities | Symptoms causing inability to perform usual social & functional activities | NA |
| Fatigue<br>Malaise | Symptoms causing no or minimal interference with usual social & functional activities | Symptoms causing greater than minimal interference with usual social & functional activities | Symptoms causing inability to perform usual social & functional activities | Incapacitating fatigue/ malaise symptoms causing inability to perform basic self-care functions |
| Fever (nonaxillary) | 37.7 – 38.6°C | 38.7 – 39.3°C | 39.4 – 40.5°C | > 40.5°C |
| Pain (indicate body site)<br>DO NOT use for pain due to injection (See Injection Site Reactions: Injection site pain)<br>See also Headache, Arthralgia, and Myalgia | Pain causing no or minimal interference with usual social & functional activities | Pain causing greater than minimal interference with usual social & functional activities | Pain causing inability to perform usual social & functional activities | Disabling pain causing inability to perform basic self-care functions OR Hospitalization (other than emergency room visit) indicated |

**Basic Self-care Functions – Adult**: Activities such as bathing, dressing, toileting, transfer/movement, continence, and feeding.

**Basic Self-care Functions – Young Children**: Activities that are age and culturally appropriate (e.g., feeding self with culturally appropriate eating implement).

**Usual Social & Functional Activities – Adult**: Adaptive tasks and desirable activities, such as going to work, shopping, cooking, use of transportation, pursuing a hobby, etc.

**Usual Social & Functional Activities – Young Children**: Activities that are age and culturally appropriate (e.g., social interactions, play activities, learning tasks, etc.).

## DIVISION OF AIDS TABLE FOR GRADING THE SEVERITY OF ADULT AND PEDIATRIC ADVERSE EVENTS
## VERSION 1.0, DECEMBER, 2004; CLARIFICATION AUGUST 2009

| PARAMETER | GRADE 1 MILD | GRADE 2 MODERATE | GRADE 3 SEVERE | GRADE 4 POTENTIALLY LIFE-THREATENING |
|---|---|---|---|---|
| Unintentional weight loss | NA | 5 – 9% loss in body weight from baseline | 10 – 19% loss in body weight from baseline | ≥ 20% loss in body weight from baseline OR Aggressive intervention indicated [e.g., tube feeding or total parenteral nutrition (TPN)] |
| **INFECTION** | | | | |
| Infection (any other than HIV infection) | Localized, no systemic antimicrobial treatment indicated AND Symptoms causing no or minimal interference with usual social & functional activities | Systemic antimicrobial treatment indicated OR Symptoms causing greater than minimal interference with usual social & functional activities | Systemic antimicrobial treatment indicated AND Symptoms causing inability to perform usual social & functional activities OR Operative intervention (other than simple incision and drainage) indicated | Life-threatening consequences (e.g., septic shock) |
| **INJECTION SITE REACTIONS** | | | | |
| Injection site pain (pain without touching) Or Tenderness (pain when area is touched) | Pain/tenderness causing no or minimal limitation of use of limb | Pain/tenderness limiting use of limb OR Pain/tenderness causing greater than minimal interference with usual social & functional activities | Pain/tenderness causing inability to perform usual social & functional activities | Pain/tenderness causing inability to perform basic self-care function OR Hospitalization (other than emergency room visit) indicated for management of pain/tenderness |
| Injection site reaction (localized) | | | | |
| **Adult > 15 years** | Erythema OR Induration of 5x5 cm – 9x9 cm (or 25 $cm^2$ – 81$cm^2$) | Erythema OR Induration OR Edema > 9 cm any diameter (or > 81 $cm^2$) | Ulceration OR Secondary infection OR Phlebitis OR Sterile abscess OR Drainage | Necrosis (involving dermis and deeper tissue) |
| **Pediatric ≤ 15 years** | Erythema OR Induration OR Edema present but ≤ 2.5 cm diameter | Erythema OR Induration OR Edema > 2.5 cm diameter but < 50% surface area of the extremity segment (e.g., upper arm/thigh) | Erythema OR Induration OR Edema involving ≥ 50% surface area of the extremity segment (e.g., upper arm/thigh) OR Ulceration OR Secondary infection OR Phlebitis OR Sterile abscess OR Drainage | Necrosis (involving dermis and deeper tissue) |

**Basic Self-care Functions – Adult**: Activities such as bathing, dressing, toileting, transfer/movement, continence, and feeding.

**Basic Self-care Functions – Young Children**: Activities that are age and culturally appropriate (e.g., feeding self with culturally appropriate eating implement).

**Usual Social & Functional Activities – Adult**: Adaptive tasks and desirable activities, such as going to work, shopping, cooking, use of transportation, pursuing a hobby, etc.

**Usual Social & Functional Activities – Young Children**: Activities that are age and culturally appropriate (e.g., social interactions, play activities, learning tasks, etc.).

**DIVISION OF AIDS TABLE FOR GRADING THE SEVERITY OF ADULT AND PEDIATRIC ADVERSE EVENTS**
**VERSION 1.0, DECEMBER, 2004; CLARIFICATION AUGUST 2009**

| PARAMETER | GRADE 1 MILD | GRADE 2 MODERATE | GRADE 3 SEVERE | GRADE 4 POTENTIALLY LIFE-THREATENING |
|---|---|---|---|---|
| Pruritis associated with injection<br>See also Skin: Pruritis (itching - no skin lesions) | Itching localized to injection site AND Relieved spontaneously or with < 48 hours treatment | Itching beyond the injection site but not generalized OR Itching localized to injection site requiring ≥ 48 hours treatment | Generalized itching causing inability to perform usual social & functional activities | NA |
| **SKIN – DERMATOLOGICAL** | | | | |
| Alopecia | Thinning detectable by study participant (or by caregiver for young children and disabled adults) | Thinning or patchy hair loss detectable by health care provider | Complete hair loss | NA |
| Cutaneous reaction – rash | Localized macular rash | Diffuse macular, maculopapular, or morbilliform rash OR Target lesions | Diffuse macular, maculopapular, or morbilliform rash with vesicles or limited number of bullae OR Superficial ulcerations of mucous membrane limited to one site | Extensive or generalized bullous lesions OR Stevens-Johnson syndrome OR Ulceration of mucous membrane involving two or more distinct mucosal sites OR Toxic epidermal necrolysis (TEN) |
| Hyperpigmentation | Slight or localized | Marked or generalized | NA | NA |
| Hypopigmentation | Slight or localized | Marked or generalized | NA | NA |
| Pruritis (itching – no skin lesions)<br>(See also Injection Site Reactions: Pruritis associated with injection) | Itching causing no or minimal interference with usual social & functional activities | Itching causing greater than minimal interference with usual social & functional activities | Itching causing inability to perform usual social & functional activities | NA |
| **CARDIOVASCULAR** | | | | |
| Cardiac arrhythmia (general)<br>(By ECG or physical exam) | Asymptomatic AND No intervention indicated | Asymptomatic AND Non-urgent medical intervention indicated | Symptomatic, non-life-threatening AND Non-urgent medical intervention indicated | Life-threatening arrhythmia OR Urgent intervention indicated |
| Cardiac-ischemia/infarction | NA | NA | Symptomatic ischemia (stable angina) OR Testing consistent with ischemia | Unstable angina OR Acute myocardial infarction |

**Basic Self-care Functions – Adult**: Activities such as bathing, dressing, toileting, transfer/movement, continence, and feeding.

**Basic Self-care Functions – Young Children**: Activities that are age and culturally appropriate (e.g., feeding self with culturally appropriate eating implement).

**Usual Social & Functional Activities – Adult**: Adaptive tasks and desirable activities, such as going to work, shopping, cooking, use of transportation, pursuing a hobby, etc.

**Usual Social & Functional Activities – Young Children**: Activities that are age and culturally appropriate (e.g., social interactions, play activities, learning tasks, etc.).

28 Dec 04/Clarification Aug 09 Page 5 of 21 Version 1.0/Clarification 1

## DIVISION OF AIDS TABLE FOR GRADING THE SEVERITY OF ADULT AND PEDIATRIC ADVERSE EVENTS
## VERSION 1.0, DECEMBER, 2004; CLARIFICATION AUGUST 2009

| PARAMETER | GRADE 1<br>MILD | GRADE 2<br>MODERATE | GRADE 3<br>SEVERE | GRADE 4<br>POTENTIALLY LIFE-THREATENING |
|---|---|---|---|---|
| Hemorrhage (significant acute blood loss) | NA | Symptomatic AND No transfusion indicated | Symptomatic AND Transfusion of ≤ 2 units packed RBCs (for children ≤ 10 cc/kg) indicated | Life-threatening hypotension OR Transfusion of > 2 units packed RBCs (for children > 10 cc/kg) indicated |
| Hypertension | | | | |
| **Adult > 17 years**<br>(with repeat testing at same visit) | 140 – 159 mmHg systolic<br>OR<br>90 – 99 mmHg diastolic | 160 – 179 mmHg systolic<br>OR<br>100 – 109 mmHg diastolic | ≥ 180 mmHg systolic<br>OR<br>≥ 110 mmHg diastolic | Life-threatening consequences (e.g., malignant hypertension) OR Hospitalization indicated (other than emergency room visit) |
| **Correction**: in Grade 2 to 160 - 179 from > 160-179 (systolic) and to ≥ 100 -109 from > 100-109 (diastolic) and in Grade 3 to ≥ 180 from > 180 (systolic) and to ≥ 110 from > 110 (diastolic). | | | | |
| **Pediatric ≤ 17 years**<br>(with repeat testing at same visit) | NA | $91^{st}$ – $94^{th}$ percentile adjusted for age, height, and gender (systolic and/or diastolic) | ≥ $95^{th}$ percentile adjusted for age, height, and gender (systolic and/or diastolic) | Life-threatening consequences (e.g., malignant hypertension) OR Hospitalization indicated (other than emergency room visit) |
| Hypotension | NA | Symptomatic, corrected with oral fluid replacement | Symptomatic, IV fluids indicated | Shock requiring use of vasopressors or mechanical assistance to maintain blood pressure |
| Pericardial effusion | Asymptomatic, small effusion requiring no intervention | Asymptomatic, moderate or larger effusion requiring no intervention | Effusion with non-life threatening physiologic consequences OR Effusion with non-urgent intervention indicated | Life-threatening consequences (e.g., tamponade) OR Urgent intervention indicated |
| Prolonged PR interval | | | | |
| **Adult > 16 years** | PR interval 0.21 – 0.25 sec | PR interval > 0.25 sec | Type II $2^{nd}$ degree AV block OR Ventricular pause > 3.0 sec | Complete AV block |
| **Pediatric ≤ 16 years** | $1^{st}$ degree AV block (PR > normal for age and rate) | Type I $2^{nd}$ degree AV block | Type II $2^{nd}$ degree AV block | Complete AV block |

**Basic Self-care Functions – Adult**: Activities such as bathing, dressing, toileting, transfer/movement, continence, and feeding.

**Basic Self-care Functions – Young Children**: Activities that are age and culturally appropriate (e.g., feeding self with culturally appropriate eating implement).

**Usual Social & Functional Activities – Adult**: Adaptive tasks and desirable activities, such as going to work, shopping, cooking, use of transportation, pursuing a hobby, etc.

**Usual Social & Functional Activities – Young Children**: Activities that are age and culturally appropriate (e.g., social interactions, play activities, learning tasks, etc.).

**DIVISION OF AIDS TABLE FOR GRADING THE SEVERITY OF ADULT AND PEDIATRIC ADVERSE EVENTS**
**VERSION 1.0, DECEMBER, 2004; CLARIFICATION AUGUST 2009**

| PARAMETER | GRADE 1 MILD | GRADE 2 MODERATE | GRADE 3 SEVERE | GRADE 4 POTENTIALLY LIFE-THREATENING |
|---|---|---|---|---|
| Prolonged QTc | | | | |
| **Adult > 16 years** | Asymptomatic, QTc interval 0.45 – 0.47 sec OR Increase interval < 0.03 sec above baseline | Asymptomatic, QTc interval 0.48 – 0.49 sec OR Increase in interval 0.03 – 0.05 sec above baseline | Asymptomatic, QTc interval ≥ 0.50 sec OR Increase in interval ≥ 0.06 sec above baseline | Life-threatening consequences, e.g. Torsade de pointes or other associated serious ventricular dysrhythmia |
| **Pediatric ≤ 16 years** | Asymptomatic, QTc interval 0.450 – 0.464 sec | Asymptomatic, QTc interval 0.465 – 0.479 sec | Asymptomatic, QTc interval ≥ 0.480 sec | Life-threatening consequences, e.g. Torsade de pointes or other associated serious ventricular dysrhythmia |
| Thrombosis/embolism | NA | Deep vein thrombosis AND No intervention indicated (e.g., anticoagulation, lysis filter, invasive procedure) | Deep vein thrombosis AND Intervention indicated (e.g., anticoagulation, lysis filter, invasive procedure) | Embolic event (e.g., pulmonary embolism, life-threatening thrombus) |
| Vasovagal episode (associated with a procedure of any kind) | Present without loss of consciousness | Present with transient loss of consciousness | NA | NA |
| Ventricular dysfunction (congestive heart failure) | NA | Asymptomatic diagnostic finding AND intervention indicated | New onset with symptoms OR Worsening symptomatic congestive heart failure | Life-threatening congestive heart failure |
| **GASTROINTESTINAL** | | | | |
| Anorexia | Loss of appetite without decreased oral intake | Loss of appetite associated with decreased oral intake without significant weight loss | Loss of appetite associated with significant weight loss | Life-threatening consequences OR Aggressive intervention indicated [e.g., tube feeding or total parenteral nutrition (TPN)] |
| **Comment:** Please note that, while the grading scale provided for Unintentional Weight Loss may be used as a guideline when grading anorexia, this is not a requirement and should not be used as a substitute for clinical judgment. | | | | |
| Ascites | Asymptomatic | Symptomatic AND Intervention indicated (e.g., diuretics or therapeutic paracentesis) | Symptomatic despite intervention | Life-threatening consequences |

**Basic Self-care Functions – Adult**: Activities such as bathing, dressing, toileting, transfer/movement, continence, and feeding.

**Basic Self-care Functions – Young Children**: Activities that are age and culturally appropriate (e.g., feeding self with culturally appropriate eating implement).

**Usual Social & Functional Activities – Adult**: Adaptive tasks and desirable activities, such as going to work, shopping, cooking, use of transportation, pursuing a hobby, etc.

**Usual Social & Functional Activities – Young Children**: Activities that are age and culturally appropriate (e.g., social interactions, play activities, learning tasks, etc.).

**DIVISION OF AIDS TABLE FOR GRADING THE SEVERITY OF ADULT AND PEDIATRIC ADVERSE EVENTS**
**VERSION 1.0, DECEMBER, 2004; CLARIFICATION AUGUST 2009**

| PARAMETER | GRADE 1 MILD | GRADE 2 MODERATE | GRADE 3 SEVERE | GRADE 4 POTENTIALLY LIFE-THREATENING |
|---|---|---|---|---|
| Cholecystitis | NA | Symptomatic AND Medical intervention indicated | Radiologic, endoscopic, or operative intervention indicated | Life-threatening consequences (e.g., sepsis or perforation) |
| Constipation | NA | Persistent constipation requiring regular use of dietary modifications, laxatives, or enemas | Obstipation with manual evacuation indicated | Life-threatening consequences (e.g., obstruction) |
| Diarrhea | | | | |
| **Adult and Pediatric ≥ 1 year** | Transient or intermittent episodes of unformed stools OR Increase of ≤ 3 stools over baseline per 24-hour period | Persistent episodes of unformed to watery stools OR Increase of 4 – 6 stools over baseline per 24-hour period | Bloody diarrhea OR Increase of ≥ 7 stools per 24-hour period OR IV fluid replacement indicated | Life-threatening consequences (e.g., hypotensive shock) |
| **Pediatric < 1 year** | Liquid stools (more unformed than usual) but usual number of stools | Liquid stools with increased number of stools OR Mild dehydration | Liquid stools with moderate dehydration | Liquid stools resulting in severe dehydration with aggressive rehydration indicated OR Hypotensive shock |
| Dysphagia-Odynophagia | Symptomatic but able to eat usual diet | Symptoms causing altered dietary intake without medical intervention indicated | Symptoms causing severely altered dietary intake with medical intervention indicated | Life-threatening reduction in oral intake |
| Mucositis/stomatitis (clinical exam)<br>Indicate site (e.g., larynx, oral)<br>See Genitourinary for Vulvovaginitis<br>See also Dysphagia-Odynophagia and Proctitis | Erythema of the mucosa | Patchy pseudomembranes or ulcerations | Confluent pseudomembranes or ulcerations OR Mucosal bleeding with minor trauma | Tissue necrosis OR Diffuse spontaneous mucosal bleeding OR Life-threatening consequences (e.g., aspiration, choking) |
| Nausea | Transient (< 24 hours) or intermittent nausea with no or minimal interference with oral intake | Persistent nausea resulting in decreased oral intake for 24 – 48 hours | Persistent nausea resulting in minimal oral intake for > 48 hours OR Aggressive rehydration indicated (e.g., IV fluids) | Life-threatening consequences (e.g., hypotensive shock) |

**Basic Self-care Functions – Adult**: Activities such as bathing, dressing, toileting, transfer/movement, continence, and feeding.

**Basic Self-care Functions – Young Children**: Activities that are age and culturally appropriate (e.g., feeding self with culturally appropriate eating implement).

**Usual Social & Functional Activities – Adult**: Adaptive tasks and desirable activities, such as going to work, shopping, cooking, use of transportation, pursuing a hobby, etc.

**Usual Social & Functional Activities – Young Children**: Activities that are age and culturally appropriate (e.g., social interactions, play activities, learning tasks, etc.).

## DIVISION OF AIDS TABLE FOR GRADING THE SEVERITY OF ADULT AND PEDIATRIC ADVERSE EVENTS
## VERSION 1.0, DECEMBER, 2004; CLARIFICATION AUGUST 2009

| PARAMETER | GRADE 1 MILD | GRADE 2 MODERATE | GRADE 3 SEVERE | GRADE 4 POTENTIALLY LIFE-THREATENING |
|---|---|---|---|---|
| Pancreatitis | NA | Symptomatic AND Hospitalization not indicated (other than emergency room visit) | Symptomatic AND Hospitalization indicated (other than emergency room visit) | Life-threatening consequences (e.g., circulatory failure, hemorrhage, sepsis) |
| Proctitis (functional-symptomatic)<br>Also see Mucositis/stomatitis for clinical exam | Rectal discomfort AND No intervention indicated | Symptoms causing greater than minimal interference with usual social & functional activities OR Medical intervention indicated | Symptoms causing inability to perform usual social & functional activities OR Operative intervention indicated | Life-threatening consequences (e.g., perforation) |
| Vomiting | Transient or intermittent vomiting with no or minimal interference with oral intake | Frequent episodes of vomiting with no or mild dehydration | Persistent vomiting resulting in orthostatic hypotension OR Aggressive rehydration indicated (e.g., IV fluids) | Life-threatening consequences (e.g., hypotensive shock) |
| **NEUROLOGIC** | | | | |
| Alteration in personality-behavior or in mood (e.g., agitation, anxiety, depression, mania, psychosis) | Alteration causing no or minimal interference with usual social & functional activities | Alteration causing greater than minimal interference with usual social & functional activities | Alteration causing inability to perform usual social & functional activities | Behavior potentially harmful to self or others (e.g., suicidal and homicidal ideation or attempt, acute psychosis) OR Causing inability to perform basic self-care functions |
| Altered Mental Status<br>For Dementia, see Cognitive and behavioral/attentional disturbance (including dementia and attention deficit disorder) | Changes causing no or minimal interference with usual social & functional activities | Mild lethargy or somnolence causing greater than minimal interference with usual social & functional activities | Confusion, memory impairment, lethargy, or somnolence causing inability to perform usual social & functional activities | Delirium OR obtundation, OR coma |
| Ataxia | Asymptomatic ataxia detectable on exam OR Minimal ataxia causing no or minimal interference with usual social & functional activities | Symptomatic ataxia causing greater than minimal interference with usual social & functional activities | Symptomatic ataxia causing inability to perform usual social & functional activities | Disabling ataxia causing inability to perform basic self-care functions |

**Basic Self-care Functions – Adult**: Activities such as bathing, dressing, toileting, transfer/movement, continence, and feeding.

**Basic Self-care Functions – Young Children**: Activities that are age and culturally appropriate (e.g., feeding self with culturally appropriate eating implement).

**Usual Social & Functional Activities – Adult**: Adaptive tasks and desirable activities, such as going to work, shopping, cooking, use of transportation, pursuing a hobby, etc.

**Usual Social & Functional Activities – Young Children**: Activities that are age and culturally appropriate (e.g., social interactions, play activities, learning tasks, etc.).

**DIVISION OF AIDS TABLE FOR GRADING THE SEVERITY OF ADULT AND PEDIATRIC ADVERSE EVENTS**
**VERSION 1.0, DECEMBER, 2004; CLARIFICATION AUGUST 2009**

| PARAMETER | GRADE 1 MILD | GRADE 2 MODERATE | GRADE 3 SEVERE | GRADE 4 POTENTIALLY LIFE-THREATENING |
|---|---|---|---|---|
| Cognitive and behavioral/attentional disturbance (including dementia and attention deficit disorder) | Disability causing no or minimal interference with usual social & functional activities OR Specialized resources not indicated | Disability causing greater than minimal interference with usual social & functional activities OR Specialized resources on part-time basis indicated | Disability causing inability to perform usual social & functional activities OR Specialized resources on a full-time basis indicated | Disability causing inability to perform basic self-care functions OR Institutionalization indicated |
| CNS ischemia (acute) | NA | NA | Transient ischemic attack | Cerebral vascular accident (CVA, stroke) with neurological deficit |
| Developmental delay – **Pediatric ≤ 16 years** | Mild developmental delay, either motor or cognitive, as determined by comparison with a developmental screening tool appropriate for the setting | Moderate developmental delay, either motor or cognitive, as determined by comparison with a developmental screening tool appropriate for the setting | Severe developmental delay, either motor or cognitive, as determined by comparison with a developmental screening tool appropriate for the setting | Developmental regression, either motor or cognitive, as determined by comparison with a developmental screening tool appropriate for the setting |
| Headache | Symptoms causing no or minimal interference with usual social & functional activities | Symptoms causing greater than minimal interference with usual social & functional activities | Symptoms causing inability to perform usual social & functional activities | Symptoms causing inability to perform basic self-care functions OR Hospitalization indicated (other than emergency room visit) OR Headache with significant impairment of alertness or other neurologic function |
| Insomnia | NA | Difficulty sleeping causing greater than minimal interference with usual social & functional activities | Difficulty sleeping causing inability to perform usual social & functional activities | Disabling insomnia causing inability to perform basic self-care functions |
| Neuromuscular weakness (including myopathy & neuropathy) | Asymptomatic with decreased strength on exam OR Minimal muscle weakness causing no or minimal interference with usual social & functional activities | Muscle weakness causing greater than minimal interference with usual social & functional activities | Muscle weakness causing inability to perform usual social & functional activities | Disabling muscle weakness causing inability to perform basic self-care functions OR Respiratory muscle weakness impairing ventilation |

**Basic Self-care Functions – Adult**: Activities such as bathing, dressing, toileting, transfer/movement, continence, and feeding.

**Basic Self-care Functions – Young Children**: Activities that are age and culturally appropriate (e.g., feeding self with culturally appropriate eating implement).

**Usual Social & Functional Activities – Adult**: Adaptive tasks and desirable activities, such as going to work, shopping, cooking, use of transportation, pursuing a hobby, etc.

**Usual Social & Functional Activities – Young Children**: Activities that are age and culturally appropriate (e.g., social interactions, play activities, learning tasks, etc.).

## DIVISION OF AIDS TABLE FOR GRADING THE SEVERITY OF ADULT AND PEDIATRIC ADVERSE EVENTS
## VERSION 1.0, DECEMBER, 2004; CLARIFICATION AUGUST 2009

| PARAMETER | GRADE 1<br>MILD | GRADE 2<br>MODERATE | GRADE 3<br>SEVERE | GRADE 4<br>POTENTIALLY LIFE-THREATENING |
|---|---|---|---|---|
| Neurosensory alteration (including paresthesia and painful neuropathy) | Asymptomatic with sensory alteration on exam or minimal paresthesia causing no or minimal interference with usual social & functional activities | Sensory alteration or paresthesia causing greater than minimal interference with usual social & functional activities | Sensory alteration or paresthesia causing inability to perform usual social & functional activities | Disabling sensory alteration or paresthesia causing inability to perform basic self-care functions |
| Seizure: (new onset) **– Adult ≥ 18 years**<br>See also Seizure: (known pre-existing seizure disorder) | NA | 1 seizure | 2 – 4 seizures | Seizures of any kind which are prolonged, repetitive (e.g., status epilepticus), or difficult to control (e.g., refractory epilepsy) |
| Seizure: (known pre-existing seizure disorder) **– Adult ≥ 18 years**<br>For worsening of existing epilepsy the grades should be based on an increase from previous level of control to any of these levels. | NA | Increased frequency of pre-existing seizures (non-repetitive) without change in seizure character OR Infrequent break-through seizures while on stable medication in a previously controlled seizure disorder | Change in seizure character from baseline either in duration or quality (e.g., severity or focality) | Seizures of any kind which are prolonged, repetitive (e.g., status epilepticus), or difficult to control (e.g., refractory epilepsy) |
| Seizure **– Pediatric < 18 years** | Seizure, generalized onset with or without secondary generalization, lasting < 5 minutes with < 24 hours post ictal state | Seizure, generalized onset with or without secondary generalization, lasting 5 – 20 minutes with < 24 hours post ictal state | Seizure, generalized onset with or without secondary generalization, lasting > 20 minutes | Seizure, generalized onset with or without secondary generalization, requiring intubation and sedation |
| Syncope (not associated with a procedure) | NA | Present | NA | NA |
| Vertigo | Vertigo causing no or minimal interference with usual social & functional activities | Vertigo causing greater than minimal interference with usual social & functional activities | Vertigo causing inability to perform usual social & functional activities | Disabling vertigo causing inability to perform basic self-care functions |

**Basic Self-care Functions – Adult**: Activities such as bathing, dressing, toileting, transfer/movement, continence, and feeding.

**Basic Self-care Functions – Young Children**: Activities that are age and culturally appropriate (e.g., feeding self with culturally appropriate eating implement).

**Usual Social & Functional Activities – Adult**: Adaptive tasks and desirable activities, such as going to work, shopping, cooking, use of transportation, pursuing a hobby, etc.

**Usual Social & Functional Activities – Young Children**: Activities that are age and culturally appropriate (e.g., social interactions, play activities, learning tasks, etc.).

**DIVISION OF AIDS TABLE FOR GRADING THE SEVERITY OF ADULT AND PEDIATRIC ADVERSE EVENTS**
**VERSION 1.0, DECEMBER, 2004; CLARIFICATION AUGUST 2009**

| PARAMETER | GRADE 1 MILD | GRADE 2 MODERATE | GRADE 3 SEVERE | GRADE 4 POTENTIALLY LIFE-THREATENING |
|---|---|---|---|---|
| **RESPIRATORY** | | | | |
| Bronchospasm (acute) | FEV1 or peak flow reduced to 70 – 80% | FEV1 or peak flow 50 – 69% | FEV1 or peak flow 25 – 49% | Cyanosis OR FEV1 or peak flow < 25% OR Intubation |
| Dyspnea or respiratory distress | | | | |
| **Adult ≥ 14 years** | Dyspnea on exertion with no or minimal interference with usual social & functional activities | Dyspnea on exertion causing greater than minimal interference with usual social & functional activities | Dyspnea at rest causing inability to perform usual social & functional activities | Respiratory failure with ventilatory support indicated |
| **Pediatric < 14 years** | Wheezing OR minimal increase in respiratory rate for age | Nasal flaring OR Intercostal retractions OR Pulse oximetry 90 – 95% | Dyspnea at rest causing inability to perform usual social & functional activities OR Pulse oximetry < 90% | Respiratory failure with ventilatory support indicated |
| **MUSCULOSKELETAL** | | | | |
| Arthralgia<br>See also Arthritis | Joint pain causing no or minimal interference with usual social & functional activities | Joint pain causing greater than minimal interference with usual social & functional activities | Joint pain causing inability to perform usual social & functional activities | Disabling joint pain causing inability to perform basic self-care functions |
| Arthritis<br>See also Arthralgia | Stiffness or joint swelling causing no or minimal interference with usual social & functional activities | Stiffness or joint swelling causing greater than minimal interference with usual social & functional activities | Stiffness or joint swelling causing inability to perform usual social & functional activities | Disabling joint stiffness or swelling causing inability to perform basic self-care functions |
| Bone Mineral Loss | | | | |
| **Adult ≥ 21 years** | BMD t-score -2.5 to -1.0 | BMD t-score < -2.5 | Pathological fracture (including loss of vertebral height) | Pathologic fracture causing life-threatening consequences |
| **Pediatric < 21 years** | BMD z-score -2.5 to -1.0 | BMD z-score < -2.5 | Pathological fracture (including loss of vertebral height) | Pathologic fracture causing life-threatening consequences |
| Myalgia (non-injection site) | Muscle pain causing no or minimal interference with usual social & functional activities | Muscle pain causing greater than minimal interference with usual social & functional activities | Muscle pain causing inability to perform usual social & functional activities | Disabling muscle pain causing inability to perform basic self-care functions |

**Basic Self-care Functions – Adult**: Activities such as bathing, dressing, toileting, transfer/movement, continence, and feeding.

**Basic Self-care Functions – Young Children**: Activities that are age and culturally appropriate (e.g., feeding self with culturally appropriate eating implement).

**Usual Social & Functional Activities – Adult**: Adaptive tasks and desirable activities, such as going to work, shopping, cooking, use of transportation, pursuing a hobby, etc.

**Usual Social & Functional Activities – Young Children**: Activities that are age and culturally appropriate (e.g., social interactions, play activities, learning tasks, etc.).

**DIVISION OF AIDS TABLE FOR GRADING THE SEVERITY OF ADULT AND PEDIATRIC ADVERSE EVENTS**
**VERSION 1.0, DECEMBER, 2004; CLARIFICATION AUGUST 2009**

| PARAMETER | GRADE 1<br>MILD | GRADE 2<br>MODERATE | GRADE 3<br>SEVERE | GRADE 4<br>POTENTIALLY LIFE-THREATENING |
|---|---|---|---|---|
| Osteonecrosis | NA | Asymptomatic with radiographic findings AND No operative intervention indicated | Symptomatic bone pain with radiographic findings OR Operative intervention indicated | Disabling bone pain with radiographic findings causing inability to perform basic self-care functions |
| **GENITOURINARY** | | | | |
| Cervicitis (symptoms)<br>(For use in studies evaluating topical study agents)<br>For other cervicitis see Infection: Infection (any other than HIV infection) | Symptoms causing no or minimal interference with usual social & functional activities | Symptoms causing greater than minimal interference with usual social & functional activities | Symptoms causing inability to perform usual social & functional activities | Symptoms causing inability to perform basic self-care functions |
| Cervicitis (clinical exam)<br>(For use in studies evaluating topical study agents)<br>For other cervicitis see Infection: Infection (any other than HIV infection) | Minimal cervical abnormalities on examination (erythema, mucopurulent discharge, or friability) OR Epithelial disruption < 25% of total surface | Moderate cervical abnormalities on examination (erythema, mucopurulent discharge, or friability) OR Epithelial disruption of 25 – 49% total surface | Severe cervical abnormalities on examination (erythema, mucopurulent discharge, or friability) OR Epithelial disruption 50 – 75% total surface | Epithelial disruption > 75% total surface |
| Inter-menstrual bleeding (IMB) | Spotting observed by participant OR Minimal blood observed during clinical or colposcopic examination | Inter-menstrual bleeding not greater in duration or amount than usual menstrual cycle | Inter-menstrual bleeding greater in duration or amount than usual menstrual cycle | Hemorrhage with life-threatening hypotension OR Operative intervention indicated |
| Urinary tract obstruction (e.g., stone) | NA | Signs or symptoms of urinary tract obstruction without hydronephrosis or renal dysfunction | Signs or symptoms of urinary tract obstruction with hydronephrosis or renal dysfunction | Obstruction causing life-threatening consequences |

**Basic Self-care Functions – Adult**: Activities such as bathing, dressing, toileting, transfer/movement, continence, and feeding.

**Basic Self-care Functions – Young Children**: Activities that are age and culturally appropriate (e.g., feeding self with culturally appropriate eating implement).

**Usual Social & Functional Activities – Adult**: Adaptive tasks and desirable activities, such as going to work, shopping, cooking, use of transportation, pursuing a hobby, etc.

**Usual Social & Functional Activities – Young Children**: Activities that are age and culturally appropriate (e.g., social interactions, play activities, learning tasks, etc.).

**DIVISION OF AIDS TABLE FOR GRADING THE SEVERITY OF ADULT AND PEDIATRIC ADVERSE EVENTS**
**VERSION 1.0, DECEMBER, 2004; CLARIFICATION AUGUST 2009**

| PARAMETER | GRADE 1 MILD | GRADE 2 MODERATE | GRADE 3 SEVERE | GRADE 4 POTENTIALLY LIFE-THREATENING |
|---|---|---|---|---|
| Vulvovaginitis (symptoms) (Use in studies evaluating topical study agents) For other vulvovaginitis see Infection: Infection (any other than HIV infection) | Symptoms causing no or minimal interference with usual social & functional activities | Symptoms causing greater than minimal interference with usual social & functional activities | Symptoms causing inability to perform usual social & functional activities | Symptoms causing inability to perform basic self-care functions |
| Vulvovaginitis (clinical exam) (Use in studies evaluating topical study agents) For other vulvovaginitis see Infection: Infection (any other than HIV infection) | Minimal vaginal abnormalities on examination OR Epithelial disruption < 25% of total surface | Moderate vaginal abnormalities on examination OR Epithelial disruption of 25 - 49% total surface | Severe vaginal abnormalities on examination OR Epithelial disruption 50 - 75% total surface | Vaginal perforation OR Epithelial disruption > 75% total surface |
| **OCULAR/VISUAL** | | | | |
| Uveitis | Asymptomatic but detectable on exam | Symptomatic anterior uveitis OR Medical intervention indicated | Posterior or pan-uveitis OR Operative intervention indicated | Disabling visual loss in affected eye(s) |
| Visual changes (from baseline) | Visual changes causing no or minimal interference with usual social & functional activities | Visual changes causing greater than minimal interference with usual social & functional activities | Visual changes causing inability to perform usual social & functional activities | Disabling visual loss in affected eye(s) |
| **ENDOCRINE/METABOLIC** | | | | |
| Abnormal fat accumulation (e.g., back of neck, breasts, abdomen) | Detectable by study participant (or by caregiver for young children and disabled adults) | Detectable on physical exam by health care provider | Disfiguring OR Obvious changes on casual visual inspection | NA |
| Diabetes mellitus | NA | New onset without need to initiate medication OR Modification of current medications to regain glucose control | New onset with initiation of medication indicated OR Diabetes uncontrolled despite treatment modification | Life-threatening consequences (e.g., ketoacidosis, hyperosmolar non-ketotic coma) |

**Basic Self-care Functions – Adult**: Activities such as bathing, dressing, toileting, transfer/movement, continence, and feeding.

**Basic Self-care Functions – Young Children**: Activities that are age and culturally appropriate (e.g., feeding self with culturally appropriate eating implement).

**Usual Social & Functional Activities – Adult**: Adaptive tasks and desirable activities, such as going to work, shopping, cooking, use of transportation, pursuing a hobby, etc.

**Usual Social & Functional Activities – Young Children**: Activities that are age and culturally appropriate (e.g., social interactions, play activities, learning tasks, etc.).

**DIVISION OF AIDS TABLE FOR GRADING THE SEVERITY OF ADULT AND PEDIATRIC ADVERSE EVENTS**
**VERSION 1.0, DECEMBER, 2004; CLARIFICATION AUGUST 2009**

| PARAMETER | GRADE 1 MILD | GRADE 2 MODERATE | GRADE 3 SEVERE | GRADE 4 POTENTIALLY LIFE-THREATENING |
|---|---|---|---|---|
| Gynecomastia | Detectable by study participant or caregiver (for young children and disabled adults) | Detectable on physical exam by health care provider | Disfiguring OR Obvious on casual visual inspection | NA |
| Hyperthyroidism | Asymptomatic | Symptomatic causing greater than minimal interference with usual social & functional activities OR Thyroid suppression therapy indicated | Symptoms causing inability to perform usual social & functional activities OR Uncontrolled despite treatment modification | Life-threatening consequences (e.g., thyroid storm) |
| Hypothyroidism | Asymptomatic | Symptomatic causing greater than minimal interference with usual social & functional activities OR Thyroid replacement therapy indicated | Symptoms causing inability to perform usual social & functional activities OR Uncontrolled despite treatment modification | Life-threatening consequences (e.g., myxedema coma) |
| Lipoatrophy (e.g., fat loss from the face, extremities, buttocks) | Detectable by study participant (or by caregiver for young children and disabled adults) | Detectable on physical exam by health care provider | Disfiguring OR Obvious on casual visual inspection | NA |

**Basic Self-care Functions – Adult**: Activities such as bathing, dressing, toileting, transfer/movement, continence, and feeding.

**Basic Self-care Functions – Young Children**: Activities that are age and culturally appropriate (e.g., feeding self with culturally appropriate eating implement).

**Usual Social & Functional Activities – Adult**: Adaptive tasks and desirable activities, such as going to work, shopping, cooking, use of transportation, pursuing a hobby, etc.

**Usual Social & Functional Activities – Young Children**: Activities that are age and culturally appropriate (e.g., social interactions, play activities, learning tasks, etc.).

**DIVISION OF AIDS TABLE FOR GRADING THE SEVERITY OF ADULT AND PEDIATRIC ADVERSE EVENTS**
**VERSION 1.0, DECEMBER, 2004; CLARIFICATION AUGUST 2009**

| LABORATORY | | | | |
|---|---|---|---|---|
| **PARAMETER** | **GRADE 1 MILD** | **GRADE 2 MODERATE** | **GRADE 3 SEVERE** | **GRADE 4** POTENTIALLY LIFE-THREATENING |
| **HEMATOLOGY** | ***Standard International Units are listed in italics*** | | | |
| Absolute CD4+ count **– Adult and Pediatric > 13 years** (HIV NEGATIVE ONLY) | 300 – 400/mm$^3$ *300 – 400/µL* | 200 – 299/mm$^3$ *200 – 299/µL* | 100 – 199/mm$^3$ *100 – 199/µL* | < 100/mm$^3$ *< 100/µL* |
| Absolute lymphocyte count **– Adult and Pediatric > 13 years** (HIV NEGATIVE ONLY) | 600 – 650/mm$^3$ *0.600 x 10$^9$ – 0.650 x 10$^9$/L* | 500 – 599/mm$^3$ *0.500 x 10$^9$ – 0.599 x 10$^9$/L* | 350 – 499/mm$^3$ *0.350 x 10$^9$ – 0.499 x 10$^9$/L* | < 350/mm$^3$ *< 0.350 x 10$^9$/L* |
| **Comment:** Values in children ≤ 13 years are not given for the two parameters above because the absolute counts are variable. | | | | |
| Absolute neutrophil count (ANC) | | | | |
| **Adult and Pediatric, > 7 days** | 1,000 – 1,300/mm$^3$ *1.000 x 10$^9$ – 1.300 x 10$^9$/L* | 750 – 999/mm$^3$ *0.750 x 10$^9$ – 0.999 x 10$^9$/L* | 500 – 749/mm$^3$ *0.500 x 10$^9$ – 0.749 x 10$^9$/L* | < 500/mm$^3$ *< 0.500 x 10$^9$/L* |
| **Infant*†, 2 – ≤ 7 days** | 1,250 – 1,500/mm$^3$ *1.250 x 10$^9$ – 1.500 x 10$^9$/L* | 1,000 – 1,249/mm$^3$ *1.000 x 10$^9$ – 1.249 x 10$^9$/L* | 750 – 999/mm$^3$ *0.750 x 10$^9$ – 0.999 x 10$^9$/L* | < 750/mm$^3$ *< 0.750 x 10$^9$/L* |
| **Infant*†, ≤1 day** | 4,000 – 5,000/mm$^3$ *4.000 x 10$^9$ – 5.000 x 10$^9$/L* | 3,000 – 3,999/mm$^3$ *3.000 x 10$^9$ – 3.999 x10$^9$/L* | 1,500 – 2,999/mm$^3$ *1.500 x 10$^9$ – 2.999 x 10$^9$/L* | < 1,500/mm$^3$ *< 1.500 x 10$^9$/L* |
| **Comment:** Parameter changed from "Infant, < 1 day" to "Infant, ≤1 day" | | | | |
| Fibrinogen, decreased | 100 – 200 mg/dL *1.00 – 2.00 g/L* OR 0.75 – 0.99 x LLN | 75 – 99 mg/dL *0.75 – 0.99 g/L* OR 0.50 – 0.74 x LLN | 50 – 74 mg/dL *0.50 – 0.74 g/L* OR 0.25 – 0.49 x LLN | < 50 mg/dL *< 0.50 g/L* OR < 0.25 x LLN OR Associated with gross bleeding |

* Values are for term infants. Preterm infants should be assessed using local normal ranges.

† Use age and sex appropriate values (e.g., bilirubin).

**DIVISION OF AIDS TABLE FOR GRADING THE SEVERITY OF ADULT AND PEDIATRIC ADVERSE EVENTS**
**VERSION 1.0, DECEMBER, 2004; CLARIFICATION AUGUST 2009**

| LABORATORY | | | | |
|---|---|---|---|---|
| **PARAMETER** | **GRADE 1 MILD** | **GRADE 2 MODERATE** | **GRADE 3 SEVERE** | **GRADE 4 POTENTIALLY LIFE-THREATENING** |
| Hemoglobin (Hgb) | | | | |
| **Comment:** The Hgb values in mmol/L have changed because the conversion factor used to convert g/dL to mmol/L has been changed from 0.155 to 0.6206 (the most commonly used conversion factor). For grading Hgb results obtained by an analytic method with a conversion factor other than 0.6206, the result must be converted to g/dL using the appropriate conversion factor for that lab. | | | | |
| **Adult and Pediatric ≥ 57 days** (HIV POSITIVE ONLY) | 8.5 – 10.0 g/dL<br>*5.24 – 6.23 mmol/L* | 7.5 – 8.4 g/dL<br>*4.62–5.23 mmol/L* | 6.50 – 7.4 g/dL<br>*4.03–4.61 mmol/L* | < 6.5 g/dL<br>< *4.03 mmol/L* |
| **Adult and Pediatric ≥ 57 days** (HIV NEGATIVE ONLY) | 10.0 – 10.9 g/dL<br>*6.18 – 6.79 mmol/L*<br>OR<br>Any decrease<br>2.5 – 3.4 g/dL<br>*1.58 – 2.13 mmol/L* | 9.0 – 9.9 g/dL<br>*5.55 - 6.17 mmol/L*<br>OR<br>Any decrease<br>3.5 – 4.4 g/dL<br>*2.14 – 2.78 mmol/L* | 7.0 – 8.9 g/dL<br>*4.34 - 5.54 mmol/L*<br>OR<br>Any decrease<br>≥ 4.5 g/dL<br>> *2.79 mmol/L* | < 7.0 g/dL<br>< *4.34 mmol/L* |
| **Comment:** The decrease is a decrease from baseline | | | | |
| **Infant*[†], 36 – 56 days** (HIV POSITIVE OR NEGATIVE) | 8.5 – 9.4 g/dL<br>*5.24 – 5.86 mmol/L* | 7.0 – 8.4 g/dL<br>*4.31 – 5.23 mmol/L* | 6.0 – 6.9 g/dL<br>*3.72 – 4.30 mmol/L* | < 6.00 g/dL<br>< *3.72 mmol/L* |
| **Infant*[†], 22 – 35 days** (HIV POSITIVE OR NEGATIVE) | 9.5 – 10.5 g/dL<br>*5.87 - 6.54 mmol/L* | 8.0 – 9.4 g/dL<br>*4.93 – 5.86 mmol/L* | 7.0 – 7.9 g/dL<br>*4.34 – 4.92 mmol/L* | < 7.00 g/dL<br>< *4.34 mmol/L* |
| **Infant*[†], ≤ 21 days** (HIV POSITIVE OR NEGATIVE) | 12.0 – 13.0 g/dL<br>*7.42 – 8.09 mmol/L* | 10.0 – 11.9 g/dL<br>*6.18 – 7.41 mmol/L* | 9.0 – 9.9 g/dL<br>*5.59- 6.17 mmol/L* | < 9.0 g/dL<br>< *5.59 mmol/L* |
| **Correction:** Parameter changed from "Infant < 21 days" to "Infant ≤ 21 days" | | | | |
| International Normalized Ratio of prothrombin time (INR) | 1.1 – 1.5 x ULN | 1.6 – 2.0 x ULN | 2.1 – 3.0 x ULN | > 3.0 x ULN |
| Methemoglobin | 5.0 – 10.0% | 10.1 – 15.0% | 15.1 – 20.0% | > 20.0% |
| Prothrombin Time (PT) | 1.1 – 1.25 x ULN | 1.26 – 1.50 x ULN | 1.51 – 3.00 x ULN | > 3.00 x ULN |
| Partial Thromboplastin Time (PTT) | 1.1 – 1.66 x ULN | 1.67 – 2.33 x ULN | 2.34 – 3.00 x ULN | > 3.00 x ULN |
| Platelets, decreased | 100,000 – 124,999/$mm^3$<br>*100.000 x $10^9$ – 124.999 x $10^9$/L* | 50,000 – 99,999/$mm^3$<br>*50.000 x $10^9$ – 99.999 x $10^9$/L* | 25,000 – 49,999/$mm^3$<br>*25.000 x $10^9$ – 49.999 x $10^9$/L* | < 25,000/$mm^3$<br>*< 25.000 x $10^9$/L* |
| WBC, decreased | 2,000 – 2,500/$mm^3$<br>*2.000 x $10^9$ – 2.500 x $10^9$/L* | 1,500 – 1,999/$mm^3$<br>*1.500 x $10^9$ – 1.999 x $10^9$/L* | 1,000 – 1,499/$mm^3$<br>*1.000 x $10^9$ – 1.499 x $10^9$/L* | < 1,000/$mm^3$<br>*< 1.000 x $10^9$/L* |

* Values are for term infants. Preterm infants should be assessed using local normal ranges.

† Use age and sex appropriate values (e.g., bilirubin).

## DIVISION OF AIDS TABLE FOR GRADING THE SEVERITY OF ADULT AND PEDIATRIC ADVERSE EVENTS
## VERSION 1.0, DECEMBER, 2004; CLARIFICATION AUGUST 2009

| LABORATORY | | | | |
|---|---|---|---|---|
| **PARAMETER** | **GRADE 1**<br>**MILD** | **GRADE 2**<br>**MODERATE** | **GRADE 3**<br>**SEVERE** | **GRADE 4**<br>**POTENTIALLY LIFE-THREATENING** |
| **CHEMISTRIES** | ***Standard International Units are listed in italics*** | | | |
| Acidosis | NA | pH < normal, but ≥ 7.3 | pH < 7.3 without life-threatening consequences | pH < 7.3 with life-threatening consequences |
| Albumin, serum, low | 3.0 g/dL – < LLN<br>*30 g/L – < LLN* | 2.0 – 2.9 g/dL<br>*20 – 29 g/L* | < 2.0 g/dL<br>*< 20 g/L* | NA |
| Alkaline Phosphatase | 1.25 – 2.5 x ULN† | 2.6 – 5.0 x ULN† | 5.1 – 10.0 x ULN† | > 10.0 x ULN† |
| Alkalosis | NA | pH > normal, but ≤ 7.5 | pH > 7.5 without life-threatening consequences | pH > 7.5 with life-threatening consequences |
| ALT (SGPT) | 1.25 – 2.5 x ULN | 2.6 – 5.0 x ULN | 5.1 – 10.0 x ULN | > 10.0 x ULN |
| AST (SGOT) | 1.25 – 2.5 x ULN | 2.6 – 5.0 x ULN | 5.1 – 10.0 x ULN | > 10.0 x ULN |
| Bicarbonate, serum, low | 16.0 mEq/L – < LLN<br>*16.0 mmol/L – < LLN* | 11.0 – 15.9 mEq/L<br>*11.0 – 15.9 mmol/L* | 8.0 – 10.9 mEq/L<br>*8.0 – 10.9 mmol/L* | < 8.0 mEq/L<br>*< 8.0 mmol/L* |
| **Comment:** Some laboratories will report this value as Bicarbonate ($HCO_3$) and others as Total Carbon Dioxide ($CO_2$). These are the same tests; values should be graded according to the ranges for Bicarbonate as listed above. | | | | |
| Bilirubin (Total) | | | | |
| **Adult and Pediatric > 14 days** | 1.1 – 1.5 x ULN | 1.6 – 2.5 x ULN | 2.6 – 5.0 x ULN | > 5.0 x ULN |
| **Infant*†, ≤ 14 days** (non-hemolytic) | NA | 20.0 – 25.0 mg/dL<br>*342 – 428 µmol/L* | 25.1 – 30.0 mg/dL<br>*429 – 513 µmol/L* | > 30.0 mg/dL<br>*> 513.0 µmol/L* |
| **Infant*†, ≤ 14 days** (hemolytic) | NA | NA | 20.0 – 25.0 mg/dL<br>*342 – 428 µmol/L* | > 25.0 mg/dL<br>*> 428 µmol/L* |
| Calcium, serum, high | | | | |
| **Adult and Pediatric ≥ 7 days** | 10.6 – 11.5 mg/dL<br>*2.65 – 2.88 mmol/L* | 11.6 – 12.5 mg/dL<br>*2.89 – 3.13 mmol/L* | 12.6 – 13.5 mg/dL<br>*3.14 – 3.38 mmol/L* | > 13.5 mg/dL<br>*> 3.38 mmol/L* |
| **Infant*†, < 7 days** | 11.5 – 12.4 mg/dL<br>*2.88 – 3.10 mmol/L* | 12.5 – 12.9 mg/dL<br>*3.11 – 3.23 mmol/L* | 13.0 – 13.5 mg/dL<br>*3.245 – 3.38 mmol/L* | > 13.5 mg/dL<br>*> 3.38 mmol/L* |
| Calcium, serum, low | | | | |
| **Adult and Pediatric ≥ 7 days** | 7.8 – 8.4 mg/dL<br>*1.95 – 2.10 mmol/L* | 7.0 – 7.7 mg/dL<br>*1.75 – 1.94 mmol/L* | 6.1 – 6.9 mg/dL<br>*1.53 – 1.74 mmol/L* | < 6.1 mg/dL<br>*< 1.53 mmol/L* |
| **Infant*†, < 7 days** | 6.5 – 7.5 mg/dL<br>*1.63 – 1.88 mmol/L* | 6.0 – 6.4 mg/dL<br>*1.50 – 1.62 mmol/L* | 5.50 – 5.90 mg/dL<br>*1.38 – 1.51 mmol/L* | < 5.50 mg/dL<br>*< 1.38 mmol/L* |
| **Comment:** Do not adjust Calcium, serum, low or Calcium, serum, high for albumin | | | | |

* Values are for term infants. Preterm infants should be assessed using local normal ranges.

† Use age and sex appropriate values (e.g., bilirubin).

**DIVISION OF AIDS TABLE FOR GRADING THE SEVERITY OF ADULT AND PEDIATRIC ADVERSE EVENTS**
**VERSION 1.0, DECEMBER, 2004; CLARIFICATION AUGUST 2009**

| LABORATORY | | | | | |
|---|---|---|---|---|---|
| **PARAMETER** | | **GRADE 1 MILD** | **GRADE 2 MODERATE** | **GRADE 3 SEVERE** | **GRADE 4 POTENTIALLY LIFE-THREATENING** |
| Cardiac troponin I (cTnI) | | NA | NA | NA | Levels consistent with myocardial infarction or unstable angina as defined by the manufacturer |
| Cardiac troponin T (cTnT) | | NA | NA | NA | ≥ 0.20 ng/mL OR Levels consistent with myocardial infarction or unstable angina as defined by the manufacturer |
| Cholesterol (fasting) | | | | | |
| | **Adult ≥ 18 years** | 200 – 239 mg/dL *5.18 – 6.19 mmol/L* | 240 – 300 mg/dL *6.20 – 7.77 mmol/L* | > 300 mg/dL *> 7.77 mmol/L* | NA |
| | **Pediatric < 18 years** | 170 – 199 mg/dL *4.40 – 5.15 mmol/L* | 200 – 300 mg/dL *5.16 – 7.77 mmol/L* | > 300 mg/dL *> 7.77 mmol/L* | NA |
| Creatine Kinase | | 3.0 – 5.9 x ULN† | 6.0 – 9.9 x ULN† | 10.0 – 19.9 x ULN† | ≥ 20.0 x ULN† |
| Creatinine | | 1.1 – 1.3 x ULN† | 1.4 – 1.8 x ULN† | 1.9 – 3.4 x ULN† | ≥ 3.5 x ULN† |

| LABORATORY | | | | | |
|---|---|---|---|---|---|
| **PARAMETER** | | **GRADE 1 MILD** | **GRADE 2 MODERATE** | **GRADE 3 SEVERE** | **GRADE 4 POTENTIALLY LIFE-THREATENING** |
| Glucose, serum, high | | | | | |
| | Nonfasting | 116 – 160 mg/dL *6.44 – 8.88 mmol/L* | 161 – 250 mg/dL *8.89 – 13.88 mmol/L* | 251 – 500 mg/dL *13.89 – 27.75 mmol/L* | > 500 mg/dL *> 27.75 mmol/L* |
| | Fasting | 110 – 125 mg/dL *6.11 – 6.94 mmol/L* | 126 – 250 mg/dL *6.95 – 13.88 mmol/L* | 251 – 500 mg/dL *13.89 – 27.75 mmol/L* | > 500 mg/dL *> 27.75 mmol/L* |
| Glucose, serum, low | | | | | |
| | **Adult and Pediatric ≥ 1 month** | 55 – 64 mg/dL *3.05 – 3.55 mmol/L* | 40 – 54 mg/dL *2.22 – 3.06 mmol/L* | 30 – 39 mg/dL *1.67 – 2.23 mmol/L* | < 30 mg/dL *< 1.67 mmol/L* |
| | **Infant*†, < 1 month** | 50 – 54 mg/dL *2.78 – 3.00 mmol/L* | 40 – 49 mg/dL *2.22 – 2.77 mmol/L* | 30 – 39 mg/dL *1.67 – 2.21 mmol/L* | < 30 mg/dL *< 1.67 mmol/L* |
| Lactate | | ULN - < 2.0 x ULN without acidosis | ≥ 2.0 x ULN without acidosis | Increased lactate with pH < 7.3 without life-threatening consequences | Increased lactate with pH < 7.3 with life-threatening consequences |

* Values are for term infants. Preterm infants should be assessed using local normal ranges.

† Use age and sex appropriate values (e.g., bilirubin).

## DIVISION OF AIDS TABLE FOR GRADING THE SEVERITY OF ADULT AND PEDIATRIC ADVERSE EVENTS
## VERSION 1.0, DECEMBER, 2004; CLARIFICATION AUGUST 2009

| | | | | |
|---|---|---|---|---|
| **Comment:** Added ULN to Grade 1 parameter | | | | |
| LDL cholesterol (fasting) | | | | |
| **Adult ≥ 18 years** | 130 – 159 mg/dL<br>*3.37 – 4.12 mmol/L* | 160 – 190 mg/dL<br>*4.13 – 4.90 mmol/L* | ≥ 190 mg/dL<br>*≥ 4.91 mmol/L* | NA |
| **Pediatric > 2 - < 18 years** | 110 – 129 mg/dL<br>*2.85 – 3.34 mmol/L* | 130 – 189 mg/dL<br>*3.35 – 4.90 mmol/L* | ≥ 190 mg/dL<br>*≥ 4.91 mmol/L* | NA |
| Lipase | 1.1 – 1.5 x ULN | 1.6 – 3.0 x ULN | 3.1 – 5.0 x ULN | > 5.0 x ULN |
| Magnesium, serum, low | 1.2 – 1.4 mEq/L<br>*0.60 – 0.70 mmol/L* | 0.9 – 1.1 mEq/L<br>*0.45 – 0.59 mmol/L* | 0.6 – 0.8 mEq/L<br>*0.30 – 0.44 mmol/L* | < 0.60 mEq/L<br>*< 0.30 mmol/L* |
| Pancreatic amylase | 1.1 – 1.5 x ULN | 1.6 – 2.0 x ULN | 2.1 – 5.0 x ULN | > 5.0 x ULN |
| Phosphate, serum, low | | | | |
| **Adult and Pediatric > 14 years** | 2.5 mg/dL – < LLN<br>*0.81 mmol/L – < LLN* | 2.0 – 2.4 mg/dL<br>*0.65 – 0.80 mmol/L* | 1.0 – 1.9 mg/dL<br>*0.32 – 0.64 mmol/L* | < 1.00 mg/dL<br>*< 0.32 mmol/L* |
| **Pediatric 1 year – 14 years** | 3.0 – 3.5 mg/dL<br>*0.97 – 1.13 mmol/L* | 2.5 – 2.9 mg/dL<br>*0.81 – 0.96 mmol/L* | 1.5 – 2.4 mg/dL<br>*0.48 – 0.80 mmol/L* | < 1.50 mg/dL<br>*< 0.48 mmol/L* |
| **Pediatric < 1 year** | 3.5 – 4.5 mg/dL<br>*1.13 – 1.45 mmol/L* | 2.5 – 3.4 mg/dL<br>*0.81 – 1.12 mmol/L* | 1.5 – 2.4 mg/dL<br>*0.48 – 0.80 mmol/L* | < 1.50 mg/dL<br>*< 0.48 mmol/L* |
| Potassium, serum, high | 5.6 – 6.0 mEq/L<br>*5.6 – 6.0 mmol/L* | 6.1 – 6.5 mEq/L<br>*6.1 – 6.5 mmol/L* | 6.6 – 7.0 mEq/L<br>*6.6 – 7.0 mmol/L* | > 7.0 mEq/L<br>*> 7.0 mmol/L* |
| Potassium, serum, low | 3.0 – 3.4 mEq/L<br>*3.0 – 3.4 mmol/L* | 2.5 – 2.9 mEq/L<br>*2.5 – 2.9 mmol/L* | 2.0 – 2.4 mEq/L<br>*2.0 – 2.4 mmol/L* | < 2.0 mEq/L<br>*< 2.0 mmol/L* |
| Sodium, serum, high | 146 – 150 mEq/L<br>*146 – 150 mmol/L* | 151 – 154 mEq/L<br>*151 – 154 mmol/L* | 155 – 159 mEq/L<br>*155 – 159 mmol/L* | ≥ 160 mEq/L<br>*≥ 160 mmol/L* |
| Sodium, serum, low | 130 – 135 mEq/L<br>*130 – 135 mmol/L* | 125 – 129 mEq/L<br>*125 – 129 mmol/L* | 121 – 124 mEq/L<br>*121 – 124 mmol/L* | ≤ 120 mEq/L<br>*≤ 120 mmol/L* |
| Triglycerides (fasting) | NA | 500 – 750 mg/dL<br>*5.65 – 8.48 mmol/L* | 751 – 1,200 mg/dL<br>*8.49 – 13.56 mmol/L* | > 1,200 mg/dL<br>*> 13.56 mmol/L* |

* Values are for term infants. Preterm infants should be assessed using local normal ranges.

† Use age and sex appropriate values (e.g., bilirubin).

**DIVISION OF AIDS TABLE FOR GRADING THE SEVERITY OF ADULT AND PEDIATRIC ADVERSE EVENTS**
**VERSION 1.0, DECEMBER, 2004; CLARIFICATION AUGUST 2009**

| LABORATORY | | | | |
|---|---|---|---|---|
| **PARAMETER** | **GRADE 1 MILD** | **GRADE 2 MODERATE** | **GRADE 3 SEVERE** | **GRADE 4 POTENTIALLY LIFE-THREATENING** |
| Uric acid | 7.5 – 10.0 mg/dL<br>*0.45 – 0.59 mmol/L* | 10.1 – 12.0 mg/dL<br>*0.60 – 0.71 mmol/L* | 12.1 – 15.0 mg/dL<br>*0.72 – 0.89 mmol/L* | > 15.0 mg/dL<br>*> 0.89 mmol/L* |
| **URINALYSIS** | ***Standard International Units are listed in italics*** | | | |
| Hematuria (microscopic) | 6 – 10 RBC/HPF | > 10 RBC/HPF | Gross, with or without clots OR with RBC casts | Transfusion indicated |
| Proteinuria, random collection | 1 + | 2 – 3 + | 4 + | NA |
| Proteinuria, 24 hour collection | | | | |
| **Adult and Pediatric ≥ 10 years** | 200 – 999 mg/24 h<br>*0.200 – 0.999 g/d* | 1,000 – 1,999 mg/24 h<br>*1.000 – 1.999 g/d* | 2,000 – 3,500 mg/24 h<br>*2.000 – 3.500 g/d* | > 3,500 mg/24 h<br>*> 3.500 g/d* |
| **Pediatric > 3 mo - < 10 years** | 201 – 499 mg/m$^2$/24 h<br>*0.201 – 0.499 g/d* | 500 – 799 mg/m$^2$/24 h<br>*0.500 – 0.799 g/d* | 800 – 1,000 mg/m$^2$/24 h<br>*0.800 – 1.000 g/d* | > 1,000 mg/ m$^2$/24 h<br>*> 1.000 g/d* |

* Values are for term infants. Preterm infants should be assessed using local normal ranges.

† Use age and sex appropriate values (e.g., bilirubin).

## 15.2 AIDS-Defining Illnesses

- Candidiasis of bronchi, trachea, or lungs
- Candidiasis, esophageal
- Cervical cancer, invasive
- Coccidioidomycosis, disseminated, or extra pulmonary
- Cryptococcosis, extra pulmonary
- Cryptosporidiosis, chronic intestinal (>1 month's duration)
- Cytomegalovirus disease (other than liver, spleen or nodes)
- Cytomegalovirus retinitis (with loss of vision)
- Encephalopathy, HIV-related
- Herpes simplex: chronic ulcer(s) (>1 month's duration); or bronchitis, pneumonitis, or esophagitis
- Histoplasmosis, disseminated, or extra pulmonary
- Isosporiasis, chronic intestinal (>1 month's duration)
- Kaposi's sarcoma
- Lymphoma, Burkitt's (or equivalent term)
- Lymphoma, immunoblastic (or equivalent term)
- Lymphoma, primary, of brain
- *Mycobacterium avium* complex or M. kansasii, disseminated, or extra pulmonary
- Mycobacterium tuberculosis, any site (pulmonary or extra pulmonary)
- Mycobacterium, other species or unidentified species disseminated or extra pulmonary
- *Pneumocystis carinii* pneumonia
- Pneumonia, recurrent
- Progressive multifocal leukoencephalopathy
- Salmonella septicemia, recurrent
- Toxoplasmosis of brain
- Wasting syndrome due to HIV

For more information refer to:

Castro KG, Ward JW, Slutsker L, Buehler JW, Berkelman RL. 1992. 1993 Revised Classification System for HIV Infection and Expanded Surveillance Case Definition for AIDS Among Adolescents and Adults. MMWR 1992; 41:1-19.

Schneider E, Whitmore S, Glynn MK, Dominguez K, Mitsch A, McKenna MT. Revised surveillance case definitions for HIV Infection among adults, adolescents and children aged < 18 months and HIV infection and AIDS among children aged 18 months to < 13 years – United States, 2008. MMWR 2008; 57: RR-10.
